# Inequalities in the burden of non-communicable diseases across European countries: a systematic analysis of the Global Burden of Disease 2019 study

**DOI:** 10.1186/s12939-023-01958-8

**Published:** 2023-07-28

**Authors:** Carlos Alexandre Soares Andrade, Nour Mahrouseh, Jonila Gabrani, Periklis Charalampous, Sarah Cuschieri, Diana Alecsandra Grad, Brigid Unim, Enkeleint A. Mechili, José Chen-Xu, Brecht Devleesschauwer, Gaetano Isola, Elena von der Lippe, Carl Michael Baravelli, Florian Fischer, Nanna Weye, Mirza Balaj, Romana Haneef, Mary Economou, Juanita A. Haagsma, Orsolya Varga

**Affiliations:** 1 Department of Public Health and Epidemiology, Faculty of Medicine, University of Debrecen, 26 Kassai Street, 4028 Debrecen, Hungary; 2 Faculty of Medicine, University of Basel, Basel, Switzerland; 3 Department of Public Health, Erasmus MC University Medical Center, Rotterdam, The Netherlands; 4 Faculty of Medicine and Surgery, University of Malta, Msida, Malta; 5 Department of Public Health, Babes-Bolyai University, Cluj-Napoca-Napoca, Romania; 6 RoNeuro Institute for Neurological Research and Diagnostic, Cluj-Napoca-Napoca, Romania; 7 Department of Cardiovascular, Endocrine-Metabolic Diseases and Aging, Istituto Superiore Di Sanità, Rome, Italy; 8 Department of Healthcare, Faculty of Health, University of Vlora, Vlora, Albania; 9 Clinic of Social and Family Medicine, School of Medicine, University of Crete, Crete, Greece; 10 Public Health Unit, Primary Healthcare Cluster Baixo Mondego, Coimbra, Portugal; 11 National School of Public Health, NOVA University of Lisbon, Lisbon, Portugal; 12 Department of Epidemiology and Public Health, Sciensano, Brussels, Belgium; 13 Department of Translational Physiology, Infectiology and Public Health, Ghent University, Merelbeke, Belgium; 14 Department of General Surgery and Surgical Medical Specialties, University of Catania, Catania, Italy; 15 Department of Epidemiology and Health Monitoring, Robert Koch Institute, Berlin, Germany; 16 Department of Disease Burden, Norwegian Institute of Public Health, Bergen, Norway; 17 Institute of Public Health, Charité - Universitätsmedizin Berlin, Berlin, Germany; 18 Department of Clinical Epidemiology, Aarhus University and Aarhus University Hospital, Aarhus, Denmark; 19 Department of Sociology and Political Science, Centre for Global Health Inequalities Research (CHAIN), Norwegian University of Science and Technology (NTNU), Trondheim, Norway; 20 Department of Non-Communicable Diseases and Injuries, Santé Publique France, Saint-Maurice, France; 21 Department of Nursing, School of Health Sciences, Cyprus University of Technology, Limassol, Cyprus

**Keywords:** Health inequality, European Union, European Economic Area, Non-communicable diseases, DALY rate, Global Burden of Disease

## Abstract

**Background:**

Although overall health status in the last decades improved, health inequalities due to non-communicable diseases (NCDs) persist between and within European countries. There is a lack of studies giving insights into health inequalities related to NCDs in the European Economic Area (EEA) countries. Therefore, the aim of the present study was to quantify health inequalities in age-standardized disability adjusted life years (DALY) rates for NCDs overall and 12 specific NCDs across 30 EEA countries between 1990 and 2019. Also, this study aimed to determine trends in health inequalities and to identify those NCDs where the inequalities were the highest.

**Methods:**

DALY rate ratios were calculated to determine and compare inequalities between the 30 EEA countries, by sex, and across time. Annual rate of change was used to determine the differences in DALY rate between 1990 and 2019 for males and females. The Gini Coefficient (GC) was used to measure the DALY rate inequalities across countries, and the Slope Index of Inequality (SII) to estimate the average absolute difference in DALY rate across countries.

**Results:**

Between 1990 and 2019, there was an overall declining trend in DALY rate, with larger declines among females compared to males. Among EEA countries, in 2019 the highest NCD DALY rate for both sexes were observed for Bulgaria. For the whole period, the highest DALY rate ratios were identified for digestive diseases, diabetes and kidney diseases, substance use disorders, cardiovascular diseases (CVD), and chronic respiratory diseases – representing the highest inequality between countries. In 2019, the highest DALY rate ratio was found between Bulgaria and Iceland for males. GC and SII indicated that the highest inequalities were due to CVD for most of the study period – however, overall levels of inequality were low.

**Conclusions:**

The inequality in level 1 NCDs DALYs rate is relatively low among all the countries. CVDs, digestive diseases, diabetes and kidney diseases, substance use disorders, and chronic respiratory diseases are the NCDs that exhibit higher levels of inequality across countries in the EEA. This might be mitigated by applying tailored preventive measures and enabling healthcare access.

**Supplementary Information:**

The online version contains supplementary material available at 10.1186/s12939-023-01958-8.

## Background

Non-communicable diseases (NCDs) comprise many chronic diseases such as cardiovascular diseases (CVDs), cancers, diabetes mellitus (DM), and respiratory diseases. The global number of deaths attributable to NCDs is 41 million people per year, which represents 74% of all deaths in 2019 [1, 2]. Despite higher mortality and fatality in the older age group, 42% of NCD-related mortality occurs in people under 70 years old [1, 2]. Most NCDs are linked to four specific health behavioral risk factors: smoking, harmful alcohol consumption, unhealthy diet, and physical inactivity which result in four specific metabolic-physiological abnormalities: elevated blood pressure, overweight/obesity, elevated blood sugar and elevated cholesterol [3, 4].

Beyond these behavioral risk factors and medical conditions, the prevalence of NCDs is associated with low-socioeconomic position (SEP) [5]. Several risk factors of NCDs, such as smoking and physical inactivity, are also related to SEP [6]. The correlation between NCDs’ prevalence and SEP also holds at the population level, where occurrence of CVDs, such as stroke and coronary heart disease, are strongly associated with lower GDP per capita and health expenditure per capita [7]. In the past decade, the prevalence of type 2 DM has increased, partly because of the population’s poor working conditions, low income and educational level [6]. However, the prevalence rate of type 2 DM in high-income countries is higher compared to low- and middle-income countries [8].

Despite improvements in the overall level of health in many European countries, there are significant inequalities in health due to NCDs [9]. The inequalities in NCD prevalence rates within European have a social gradient and socioeconomic gaps, with lower classes experiencing higher NCD rates for most diseases [10]. Reducing these disparities has long been recognized as a major public health challenge [11].

Health inequalities refer to unjust and avoidable differences in people's health status, both within and between population groups [12]. The impact of health inequalities on society is well illustrated by the magnitude of the economic costs of socioeconomic inequalities in health. A study in 2011 showed that inequalities in health cause more than 700,000 deaths and 33 million cases of illness across the entire EU, annually [13]. They are responsible for 20% of the total healthcare expenditure and 15% of social security expenditure [13]. Inequality-related health losses diminish labor productivity and cut GDP by 1.4% a year, and the monetary value of inequality-related welfare losses is estimated by the study at €980 billion a year, or 9.4% of GDP in the EU [13].

Health inequalities in Europe have been a subject of extensive research, mostly focused on prevalence and mortality inequalities between eastern, western, and central regions [14–17]. Most studies show significant inequalities in health, especially between eastern and western countries, with almost all health indicators being worse in eastern countries than in Western Europe [18, 19]. The prevalence rates of diabetes mellitus, high blood pressure, obesity, and tobacco use are higher in Eastern Europe compared to Western Europe [20]. Many factors contribute to these inequalities, including differences in health literacy, access to healthcare services, economic situation in a country, and actual national health policies [21–23]. Health inequalities between eastern and Western Europe are still high, as post-socialist countries now exhibit greater disparities than western countries [20]. The inequalities appear to be higher in Eastern European regions than in Western Europe, most specifically for mental disorders and cancers. A study reported lower health inequalities in mortality in certain southern European countries and significant disparities prevalent in the eastern and Baltic regions [16]. Geographical health inequalities often mirror underlying disparities in socioeconomic levels, where wealthier countries tend to exhibit better health outcomes [22]. Efforts to mitigate these disparities could focus on enhancing educational opportunities, income distribution, health-related behaviors, and access to healthcare [24].

It is important to take differences between males and females into account when investigating health inequalities [25]. In Europe, even though NCDs are accountable for the highest burden of disease both for males and females, there are differences in their exposure to risk factors, social determinants of health, and access to health care services [25]. Men smoke and consume alcohol more than women. Furthermore, women are more prone to engage in preventive behavior, and have a higher intake of fruits and vegetables [26–28]. A number of EU level and national policies are being developed to address sex inequalities, optimizing equality in public services and even tackling women's unpaid care work [29–31].

In the EU, which consists of high-income countries (except Bulgaria, which is upper middle income) [32], the high disease burden of NCDs has been on the political agenda for more than 30 years [33]. The EU has considerable competence in the field of health; however, the EU has no legislative power over member states’ healthcare systems [34, 35]. Disease prevention and early detection are also essentially a competency of national authorities. Nevertheless, there are several EU initiatives, such as the “Healthier together – EU non-communicable diseases initiative” aiming to identify and implement effective policies in order to tackle NCDs [31]. The European Commission is determined to support EU Member States in their efforts to achieve the target of reducing NCD mortality under the Sustainable Development Goal (SDG) 3.4: “By 2030, reduce by one third premature mortality from non-communicable diseases through prevention and treatment and promote mental health and well-being” [36]. However, in fact, according to the 2030 Agenda for Sustainable Development adopted by the UN in 2015, only a slight reduction in NCD mortality was achieved by 2020 and efforts to tackle NCDs must be redoubled [37]. The slow progress was painfully highlighted by the exceptionally high COVID-19 mortality rate among individuals living with certain types of NCDs [38, 39].

The Global Burden of Disease (GBD) study provides a tool for quantifying health losses from hundreds of diseases, injuries and risk factors, under the leadership of the Institute for Health Metrics and Evaluation (IHME). Estimates of the GBD help policy makers understand the nature of their country's health challenges as well as the extent of health inequalities, especially in countries where subnational GBD estimates are available [40, 41]. The disability-adjusted life year (DALY) metric was created and first published in the GBD 1990 study [42] in order to quantify health effects while integrating information on mortality, morbidity, and disability [43]. Previous GBD studies reported that NCDs were accountable for 87% of disease burden in member states of the EU. The high disease burden underlines the immense rise of years lived with conditions such as ischemic heart disease, stroke, and depressive disorder [31, 44]. Furthermore, the top four risk factors attributable to DALY of NCDs are high systolic blood pressure (14.57%), smoking (11.54%), high fasting plasma glucose (10.4%), and high body-mass index (9.91%) [45]. In the EU, the change of age-standardized DALY rate between 2007 and 2017 for the top four risk factors were respectively: -22.6, -18.3, -5.7, and -9.7 [46].

Given the lack of studies giving insights into health inequalities related to NCDs in the countries of the European Economic Area (EEA), the overall goal of the present systematic analysis of GBD DALY estimates was to determine health inequalities in age-standardized DALY rate for NCDs overall and 12 specific NCDs across 30 EEA countries between 1990 and 2019. Accordingly, the objectives were to 1) provide a description of age-standardized NCDs DALY rate by country and sex for 2019; 2) present an age-standardized NCDs DALY rate for each country between 1990 and 2019 by sex, 3) determine age-standardized NCDs DALY rate ratios by country and sex between 1990 and 2019, and 4) assess health inequalities of NCDs by calculating Gini coefficient (GC) and Slope Index of Inequality (SII) between 1990 and 2019.

## Material and methods

### Study design and data source

This study is a secondary analysis of age-standardized DALY rate per 100,000 population of NCDs over a 30-year follow-up period—from 1990 to 2019 – of the GBD 2019 study [47]. One DALY should be interpreted as one lost year of healthy life. DALYs are calculated by adding Years of Life Lost (YLL), a measure of healthy time lost due to premature mortality, and Years Lived with Disability (YLDs), a measure of healthy time lost due to living with disease or injury. We retrieved age-standardized NCD DALY rate by sex, country, and year, using GBD 2019 interactive data visualization tool ‘GBD Compare’ [48] and ‘GBD Results’ [49]. The GBD 2019 study offers an extensive global, regional, and national data source for 204 countries, including 30 Member States of EEA (in 2019). A wide-ranging source of estimates is available for 369 diseases and injuries, 286 causes of death, 3484 sequelae, 87 risk factors, 23 age groups, both sexes, for a time-range from 1990 to 2019 [18, 50–52]. A detailed description of the GBD methods to calculate DALYs is given by prior publication [47].

Since the prevalence and incidence of NCDs vary by age, we have chosen to perform our analysis by using global age-standardized rates provided by the GBD tool. Age-standardized rates allow comparing health outcomes across countries and time, are consequently often used for benchmarking disease burden studies [18]. Specific data were analyzed separately for males and females. Age-standardized DALYs per 100,000 population was used to assess the total amount of healthy life lost due to NCDs level 1 and 2.

### Categorization of non-communicable diseases

The GBD database organizes the included conditions in 4 different hierarchical category levels. The first level is divided into Group I: communicable, maternal, neonatal, and nutritional diseases; Group II: NCDs; and Group III: injuries. Level 2 diseases are the subdivisions of level 1 groups, there are 22 disease and injury aggregate groupings. Level 3 and 4 include specific causes. For certain diseases, level 3 causes are the most detailed classification, while for others a more detailed categorization is defined at level 4. Our analysis was limited to NCDs at level 1 (Group I and III were excluded) and level 2: cardiovascular diseases (CVDs), chronic respiratory diseases, diabetes and kidney diseases, digestive diseases, mental disorders, musculoskeletal disorders, neoplasms, neurological disorders, sense organ diseases, skin and subcutaneous diseases, substance use disorders, and other NCDs (such as congenital birth defects, gynecological diseases, oral disorders, endocrine, metabolic, blood, and immune disorders).

### Target countries

The following 30 EEA Member States were included in our study: Austria, Belgium, Bulgaria, Croatia, Czechia, Denmark, Estonia, Finland, France, Germany, Greece, Hungary, Iceland, Ireland, Italy, Latvia, Lithuania, Luxembourg, Malta, the Netherlands, Norway, Poland, Portugal, Republic of Cyprus, Romania, Slovakia, Slovenia, Spain, Sweden, and the United Kingdom. The EEA was founded by the Agreement on the European Economic Area, an international agreement allowing the extension of the EU's single market to the member countries of the European Free Trade Association (Iceland, Liechtenstein, and Norway). The United Kingdom (UK) was part of the EEA in 2019 – for this reason, we included the UK in the analysis. Since the GBD 2019 database does not report data for Liechtenstein, it was excluded from our study.

### Statistical analysis

We used DALY rate ratios to compare inequalities between the 30 countries, by sex, and across time – a similar methodology was used elsewhere [18]. This ratio is calculated by dividing the age-standardized DALY rate of two countries, in which the higher-ranking is the numerator and the lower-ranking is the denominator:

$$\frac{Higher\;ranking\;age\;standardized\;DALY\;rate}{Lower\;ranking\;age\;standardized\;DALY\;rate}=Ratio$$

In order to compare the inequality between country-pairs in 2019, the DALY rate ratios are calculated for each country-pairs (higher-ranking/lower-ranking), by sex. This calculation yields 29 different DALY rate ratios for each of the countries presented. Values closer to “1” indicate equality between the two countries being compared, and values above “1” indicate inequality [18].

To determine the differences in DALY rate between 1990 and 2019 for males and females, we used annual rate of change, which is calculated using linear regression of the natural log of the mortality rate by year of death and expressed as a percentage by calculating the exponential of the β-coefficient minus one:

$$\left(exponential\left(\beta\;coef\;ficient\left(LN\left[X\right]\right)\right)-1\right)=Annual\;rate\;of\;change$$

The lower the annual rate of change, the greater the decline in the DALY rate between 1990 and 2019. Furthermore, the annual rate of change can be negative, indicating a decrease DALY rate, or positive, indicating an increase in DALY rate [47]. Maps were plotted to show changes in age-standardized level 1 NCD DALYs (annual rate changes) stratified by EEA member states and sex between 1990 and 2019.

We also calculated the DALY rate ratio for each year and each level 2 NCDs by dividing the highest-ranking country DALY rate by the lowest one of each year from 1990 to 2019. Rates closest to "1" indicate equality in diseases between countries, and rates above "1" indicate greater inequality between countries.

The GC, from the Lorenz curve family, was used to measure the DALY rate inequalities across countries. GC is used to analyze the extent in inequality between values, and how far these values are from equal distribution, in this case DALY rate. GC is usually defined based on the Lorenz curve, which plots the cumulative proportion of the DALY rate of the countries by the cumulative proportion of population. The line drawn at 45 degrees thus represents perfect equality. The GC is twice the ratio of the area that lies between the line of equality and the Lorenz curve. GC ranges from 0 to 1, in which 0 represents perfect equality and 1 means total inequality [53]. GC was calculated by Stata using ineqdeco (Stata module to calculate inequality indices with decomposition by subgroup) with bootstrap resampling to calculate 95% confidence intervals [54]. The SII was used as an additional measure of health inequality, that was used to estimate the average absolute difference in DALY rate across countries. This measure is based on the beta coefficient (slope) of the linear regression of Pen’s Parade, which ranked all countries by their DALY rate from lowest to highest, with the share to the total population of the included countries. Both measures are used to estimate inequality in DALY rate across countries [53, 55].

The GBD database provides data with 95% uncertainty intervals (95% UI) which reflect the variability and potential error in the modeling process providing a range of plausible values within the true DALY rate is expected to lie [47]. The DALY rate estimates are calculated by sampling 1000 draws of the posterior distribution. The DALY rate was reported in the present study as the mean value of the 1000 draws estimate. The 95% UI is represented by the 2.5th and 97.5th percentiles of the corresponding distribution. The 95% UI was interpreted as indicating a statistical difference if they did not overlap: if two or more countries had DALYs within the same 95% UI, they were considered to have no statistical difference.

The data was downloaded directly from GBD Results Tool and organized in Microsoft Excel [56]. All the age standardized DALY rate ratio and GC were calculated by using ineqdeco module in STATA [57]. Tables and graphs were generated via Excel [56] and the maps were designed with MapChart [58].

## Results

### Age-standardized NCDs DALY rate in 2019

#### Age-standardized DALY rates for NCDs level 1 and 2 by countries, 2019

Table 1 provides level 1 and 2 NCDs age-standardized DALY rates per 100,000 population and 95% UI for the 30 Member States of EEA. The NCDs DALY rate ranged from a high of 24,342 (95% UI: 20,406 to 28,775) in Bulgaria to a low of 14,845 (95% UI: 12,379 to 17,682) in Iceland. CVD contributed most to the NCDs DALY rate in Bulgaria with 9,570 (95% UI: 7,964 to 11,490) representing 39.3% of NCDs, followed by Romania with 6,644 (95% UI: 5,673 to 7,840) representing 32.2%, and Latvia with 6,603 (95% UI: 5,695 to 7,727) representing 32.1%. The lowest DALY rate due to CVD was observed in Iceland with 1,853 (95% UI: 1,669 to 2,032) being 12.5% of total NCDs, Spain with 1,834 (95% UI: 1,699 to 1,958) being 11.9%, and France with 1,628 (95% UI: 1,489 to 1,742) representing 10.5%. NCDs with lowest DALY rate and percentage were sense organ diseases in Sweden with 340 (95% UI: 227 to 487) and 2.2%, substance use disorders in Italy with 344 (95% UI: 255 to 445) also with 2.2%, and chronic respiratory diseases from Estonia with 354 (95% UI: 294 to 426) and 1.9%.

**Table 1:** Age-standardized DALY rate per 100,000 population by NCDs and EEA Member States, 2019

**Cause**	**Austria**			**Belgium**			**Bulgaria**			**Croatia**			**Cyprus**		
	**DALY rate**	**95%UI**	**(%)**	**DALY rate**	**95%UI**	**(%)**	**DALY rate**	**95%UI**	**(%)**	**DALY rate**	**95%UI**	**(%)**	**DALY rate**	**95%UI**	**(%)**
**NCDs**	**16,239**	**(13,738–19,152)**	**(100)**	**16,513**	**(13,920–19,413)**	**(100)**	**24,342**	**(20,406–28,775)**	**(100)**	**17,974**	**(14,981–21,351)**	**(100)**	**16,599**	**(14,057–19,448)**	**(100)**
**CVDs**	2,408	(2,225–2,552)	(14.8)	2,021	(1,879–2,144)	(12.2)	9,570	(7,964–11,490)	(39.3)	4,267	(3,532–5,131)	(23.7)	2,939	(2,629–3,303)	(17.7)
**Chronic respiratory diseases**	651	(544–777)	(4.0)	879	(763–1,012)	(5.3)	693	(576–819)	(2.8)	632	(524–748)	(3.5)	864	(707–1,028)	(5.2)
**Diabetes and kidney diseases**	790	(654–953)	(4.9)	694	(548–873)	(4.2)	1,265	(1,039–1,516)	(5.2)	981	(775–1,229)	(5.5)	1,165	(972–1,388)	(7.0)
**Digestive diseases**	674	(595–768)	(4.1)	641	(580–714)	(3.9)	1,282	(1,045–1,554)	(5.3)	898	(731–1,083)	(5.0)	483	(423–555)	(2.9)
**Mental disorders**	1,905	(1,388–2,518)	(11.7)	1,875	(1,369–2,467)	(11.4)	1,349	(991–1,785)	(5.5)	1,451	(1,059–1,919)	(8.1)	1,915	(1,390–2,541)	(11.5)
**Musculoskeletal disorders**	1,971	(1,413–2,650)	(12.1)	2,042	(1,461–2,729)	(12.4)	1,544	(1,096–2,061)	(6.3)	1,600	(1,141–2,117)	(8.9)	2,225	(1,597–2,963)	(13.4)
**Neoplasms**	2,820	(2,682–2,968)	(17.4)	3,256	(3,096–3,421)	(19.7)	4,368	(3,485–5,458)	(17.9)	3,770	(2,989–4,719)	(21.0)	2,648	(2,366–2,944)	(16.0)
**Neurological disorders**	1,326	(734–2,203)	(8.2)	1,579	(775–2,769)	(9.6)	1,313	(784–2,066)	(5.4)	1,289	(732–2,047)	(7.2)	1,330	(690–2,273)	(8.0)
**Sense organ diseases**	392	(263–558)	(2.4)	409	(279–580)	(2.5)	618	(412–888)	(2.5)	606	(407–879)	(3.4)	397	(268–569)	(2.4)
**Skin and subcutaneous diseases**	711	(482–1,013)	(4.4)	730	(504–1,024)	(4.4)	454	(300–669)	(1.9)	444	(293–654)	(2.5)	719	(500–1,021)	(4.3)
**Substance use disorders**	659	(516–816)	(4.1)	601	(476–750)	(3.6)	384	(291–497)	(1.6)	523	(406–660)	(2.9)	358	(267–461)	(2.2)
**Other NCDs**	1,932	(1,486–2,487)	(11.9)	1,788	(1,348–2,322)	(10.8)	1,503	(1,189–1,904)	(6.2)	1,513	(1,157–1,950)	(8.4)	1,556	(1,184–2,026)	(9.4)
**Cause**	**Czechia**			**Denmark**			**Estonia**			**Finland**			**France**		
	**DALY rate**	**95%UI**	**(%)**	**DALY rate**	**95%UI**	**(%)**	**DALY rate**	**95%UI**	**(%)**	**DALY rate**	**95%UI**	**(%)**	**DALY rate**	**95%UI**	**(%)**
**NCDs**	**17,125**	**(14,441–20,203)**	**(100)**	**17,166**	**(14,672–19,955)**	**(100)**	**18,874**	**(15,777–22,305)**	**(100)**	**16,428**	**(13,963–19,205)**	**(100)**	**15,461**	**(13,059–18,230)**	**(100)**
**CVDs**	3,909	(3,314–4,605)	(22.8)	1,968	(1,833–2,091)	(11.5)	4,651	(3,813–5,715)	(24.6)	2,864	(2,653–3,031)	(17.4)	1,628	(1,489–1,742)	(10.5)
**Chronic respiratory diseases**	628	(529–728)	(3.7)	1030	(867–1,167)	(6.0)	354	(294–426)	(1.9)	618	(512–756)	(3.8)	554	(445–692)	(3.6)
**Diabetes and kidney diseases**	1,251	(960–1,590)	(7.3)	678	(576–798)	(4.0)	752	(600–928)	(4.0)	685	(512–889)	(4.2)	463	(383–558)	(3.0)
**Digestive diseases**	896	(748–1,062)	(5.2)	686	(616–768)	(4.0)	987	(805–1,203)	(5.2)	788	(717–873)	(4.8)	565	(512–629)	(3.7)
**Mental disorders**	1,385	(1,014–1,825)	(8.1)	1,794	(1,303–2,376)	(10.5)	1,573	(1,145–2,081)	(8.3)	1,887	(1,387–2,497)	(11.5)	2,045	(1,489–2,717)	(13.2)
**Musculoskeletal disorders**	1,548	(1,110–2,065)	(9.0)	2,485	(1,781–3,292)	(14.5)	1,510	(1,080–2,011)	(8.0)	2,037	(1,467–2,717)	(12.4)	2,055	(1,474–2,732)	(13.3)
**Neoplasms**	3,405	(2,813–4,137)	(19.9)	3,494	(3,304–3,675)	(20.4)	3,522	(2,774–4,417)	(18.7)	2,606	(2,442–2,778)	(15.9)	3,311	(3,133–3,473)	(21.4)
**Neurological disorders**	1,269	(725–2,038)	(7.4)	1,282	(712–2,110)	(7.5)	1,337	(810–2,094)	(7.1)	1,396	(763–2,301)	(8.5)	1,426	(791–2,290)	(9.2)
**Sense organ diseases**	595	(398–861)	(3.5)	374	(250–537)	(2.2)	642	(427–932)	(3.4)	388	(262–553)	(2.4)	402	(273–572)	(2.6)
**Skin and subcutaneous diseases**	454	(300–669)	(2.7)	776	(529–1,106)	(4.5)	553	(357–805)	(2.9)	748	(515–1,058)	(4.6)	843	(578–1,180)	(5.5)
**Substance use disorders**	515	(398–648)	(3.0)	890	(743–1,052)	(5.2)	1,646	(1,350–1,989)	(8.7)	925	(785–1,079)	(5.6)	567	(453–697)	(3.7)
**Other NCDs**	1,271	(947–1,677)	(7.4)	1,709	(1,290–2,218)	(10.0)	1,345	(1,009–1,758)	(7.1)	1,484	(1,114–1,945)	(9.0)	1,602	(1,228–2,056)	(10.4)
**Cause**	**Germany**			**Greece**			**Hungary**			**Iceland**			**Ireland**		
	**DALY rate**	**95%UI**	**(%)**	**DALY rate**	**95%UI**	**(%)**	**DALY rate**	**95%UI**	**(%)**	**DALY rate**	**95%UI**	**(%)**	**DALY rate**	**95%UI**	**(%)**
**NCDs**	**17,277**	**(14,710–20,233)**	**(100)**	**17,222**	**(14,700–20,068)**	**(100)**	**20,458**	**(17,350–23,915)**	**(100)**	**14,845**	**(12,379–17,682)**	**(100)**	**16,792**	**(14,166–19,735)**	**(100)**
**CVDs**	2,601	(2,422–2,741)	**(100)**	3,198	(3,001–3,351)	(18.6)	5,420	(4,612–6,359)	(26.5)	1,853	(1,669–2,032)	(12.5)	2,193	(1,990–2,338)	(13.1)
**Chronic respiratory diseases**	749	(651–857)	(15.1)	724	(611–843)	(4.2)	947	(816–1,099)	(4.6)	768	(635–936)	(5.2)	969	(830–1,128)	(5.8)
**Diabetes and kidney diseases**	894	(723–1,099)	(4.3)	790	(638–967)	(4.6)	1,020	(815–1,254)	(5.0)	533	(408–683)	(3.6)	622	(491–770)	(3.7)
**Digestive diseases**	742	(677–816)	(5.2)	470	(412–548)	(2.7)	1,269	(1,063–1,489)	(6.2)	355	(305–411)	(2.4)	465	(413–533)	(2.8)
**Mental disorders**	1,899	(1,368–2,515)	(4.3)	2,260	(1,664–2,968)	(13.1)	1,394	(1,014–1,851)	(6.8)	1,761	(1,283–2,342)	(11.9)	2,202	(1,612–2,884)	(13.1)
**Musculoskeletal disorders**	2,211	(1,583–2,927)	(11.0)	2,031	(1,463–2,694)	(11.8)	1,605	(1,149–2,135)	(7.8)	2,253	(1,593–3,017)	(15.2)	2,273	(1,644–3,028)	(13.5)
**Neoplasms**	3,221	(3,063–3,368)	(12.8)	3,300	(3,134–3,471)	(19.2)	4,551	(3,772–5,494)	(22.2)	2,690	(2,449–2,963)	(18.1)	3,049	(2,859–3,244)	(18.2)
**Neurological disorders**	1,539	(811–2,545)	(18.6)	1,330	(662–2,277)	(7.7)	1,260	(729–2,007)	(6.2)	1,385	(749–2,286)	(9.3)	1,404	(766–2,318)	(8.4)
**Sense organ diseases**	393	(267–559)	(8.9)	419	(285–594)	(2.4)	614	(412–885)	(3.0)	399	(269–570)	(2.7)	397	(269–569)	(2.4)
**Skin and subcutaneous diseases**	717	(485–1,039)	(2.3)	677	(465–970)	(3.9)	489	(324–716)	(2.4)	753	(515–1,064)	(5.1)	728	(500–1,031)	(4.3)
**Substance use disorders**	618	(501–751)	(4.1)	432	(333–540)	(2.5)	490	(378–619)	(2.4)	672	(544–822)	(4.5)	824	(654–1,007)	(4.9)
**Other NCDs**	1,692	(1,287–2,189)	(3.6)	1,591	(1,221–2,077)	(9.2)	1,399	(1,084–1,781)	(6.8)	1,423	(1,052–1,872)	(9.6)	1,666	(1,283–2,137)	(9.9)
**Cause**	**Italy**			**Latvia**			**Lithuania**			**Luxembourg**			**Malta**		
	**DALY rate**	**95%UI**	**(%)**	**DALY rate**	**95%UI**	**(%)**	**DALY rate**	**95%UI**	**(%)**	**DALY rate**	**95%UI**	**(%)**	**DALY rate**	**95%UI**	**(%)**
**NCDs**	**15,753**	**(13,159–18,646)**	**(100)**	**20,566**	**(17,662–23,817)**	**(100)**	**20,070**	**(17,138–23,241)**	**(100)**	**15,740**	**(13,161–18,606)**	**(100)**	**15,953**	**(13,411–18,839)**	**(100)**
**CVDs**	2,032	(1,854–2,156)	(12.9)	6,603	(5,695–7,727)	(32.1)	5,824	(4,926–6,939)	(29.0)	1,981	(1,766–2,213)	(12.6)	2,511	(2,256–2,770)	(15.7)
**Chronic respiratory diseases**	539	(454–635)	(3.4)	391	(320–484)	(1.9)	424	(353–510)	(2.1)	787	(653–953)	(5.0)	674	(547–828)	(4.2)
**Diabetes and kidney diseases**	814	(658–992)	(5.2)	746	(600–923)	(3.6)	547	(434–683)	(2.7)	804	(618–1,020)	(5.1)	919	(744–1,129)	(5.8)
**Digestive diseases**	644	(553–752)	(4.1)	1,069	(903–1,256)	(5.2)	1,447	(1,215–1,729)	(7.2)	610	(531–700)	(3.9)	429	(371–495)	(2.7)
**Mental disorders**	1,954	(1,434–2,586)	(12.4)	1,614	(1,184–2,122)	(7.8)	1,716	(1,256–2,262)	(8.6)	1,850	(1,352–2,442)	(11.8)	1,903	(1,387–2,522)	(11.9)
**Musculoskeletal disorders**	2,184	(1,562–2,889)	(13.9)	1,531	(1,097–2,054)	(7.4)	1,496	(1,065–1,988)	(7.5)	2,141	(1,538–2,848)	(13.6)	2,224	(1,600–2,953)	(13.9)
**Neoplasms**	2,976	(2,823–3,081)	(18.9)	3,631	(3,056–4,324)	(17.7)	3,573	(2,929–4,312)	(17.8)	2,933	(2,618–3,306)	(18.6)	2,604	(2,358–2,889)	(16.3)
**Neurological disorders**	1,472	(740–2,567)	(9.3)	1,260	(758–1,994)	(6.1)	1,242	(748–1,967)	(6.2)	1,366	(753–2,213)	(8.7)	1,329	(711–2,222)	(8.3)
**Sense organ diseases**	530	(367–733)	(3.4)	691	(465–989)	(3.4)	688	(463–989)	(3.4)	380	(253–547)	(2.4)	405	(274–576)	(2.5)
**Skin and subcutaneous diseases**	733	(497–1,050)	(4.7)	419	(286–615)	(2.0)	441	(296–648)	(2.2)	728	(501–1,039)	(4.6)	748	(528–1,028)	(4.7)
**Substance use disorders**	344	(255–445)	(2.2)	1,056	(864–1,267)	(5.1)	1,053	(879–1,242)	(5.2)	664	(530–820)	(4.2)	466	(360–591)	(2.9)
**Other NCDs**	1,532	(1,156–1,999)	(9.7)	1,554	(1,192–1,986)	(7.6)	1,619	(1,242–2,073)	(8.1)	1,496	(1,108–1,976)	(9.5)	1,740	(1,350–2,224)	(10.9)
**Cause**	**Netherlands**			**Norway**			**Poland**			**Portugal**			**Romania**		
	**DALY rate**	**95%UI**	**(%)**	**DALY rate**	**95%UI**	**(%)**	**DALY rate**	**95%UI**	**(%)**	**DALY rate**	**95%UI**	**(%)**	**DALY rate**	**95%UI**	**(%)**
**NCDs**	**16,215**	**(13,797–19,002)**	**(100)**	**15,642**	**(13,123–18,527)**	**(100)**	**18,313**	**(15,640–21,393)**	**(100)**	**16,664**	**(14,085–19,599)**	**(100)**	**20,643**	**(17,686–23,857)**	**(100)**
**CVDs**	1,883	(1,737–2,009)	(11.6)	1,901	(1,747–2,027)	(12.2)	4,183	(3,595–4,832)	(22.8)	2,150	(1,997–2,282)	**(100)**	6,644	(5,673–7,840)	(32.2)
**Chronic respiratory diseases**	977	(816–1,136)	(6.0)	889	(719–1,049)	(5.7)	651	(541–783)	(3.6)	861	(706–1,064)	(12.9)	714	(602–851)	(3.5)
**Diabetes and kidney diseases**	637	(519–775)	(3.9)	650	(516–807)	(4.2)	951	(752–1,160)	(5.2)	980	(798–1,199)	(5.2)	781	(629–961)	(3.8)
**Digestive diseases**	438	(394–491)	(2.7)	510	(431–606)	(3.3)	1,033	(874–1,211)	(5.6)	624	(571–690)	(5.9)	1,501	(1,269–1,782)	(7.3)
**Mental disorders**	2,069	(1,519–2,734)	(12.8)	1,945	(1,427–2,580)	(12.4)	1,259	(930–1,651)	(6.9)	2,317	(1,672–3,082)	(3.7)	1,368	(1,007–1,805)	(6.6)
**Musculoskeletal disorders**	2,001	(1,435–2,655)	(12.3)	1,879	(1,333–2,519)	(12.0)	1,629	(1,167–2,169)	(8.9)	2,256	(1,628–2,974)	(13.9)	1,579	(1,122–2,113)	(7.6)
**Neoplasms**	3,614	(3,411–3,796)	(22.3)	2,844	(2,705–2,968)	(18.2)	4,192	(3,527–4,913)	(22.9)	3,135	(2,980–3,302)	(13.5)	3,998	(3,275–4,799)	(19.4)
**Neurological disorders**	1,414	(787–2,320)	(8.7)	1,474	(794–2,439)	(9.4)	1,337	(800–2,121)	(7.3)	1,291	(668–2,200)	(18.8)	1,249	(703–2,034)	(6.1)
**Sense organ diseases**	371	(249–538)	(2.3)	470	(317–664)	(3.0)	637	(431–912)	(3.5)	413	(283–589)	(7.7)	623	(416–896)	(3.0)
**Skin and subcutaneous diseases**	721	(497–1,019)	(4.4)	716	(484–1,022)	(4.6)	460	(307–676)	(2.5)	733	(504–1,038)	(2.5)	421	(281–624)	(2.0)
**Substance use disorders**	428	(325–538)	(2.6)	668	(555–790)	(4.3)	765	(609–934)	(4.2)	477	(344–634)	(4.4)	361	(274–463)	(1.7)
**Other NCDs**	1,663	(1,263–2,132)	(10.3)	1,697	(1,271–2,213)	(10.9)	1,216	(941–1,582)	(6.6)	1,428	(1,086–1,855)	(2.9)	1,405	(1,087–1,811)	(6.8)
**Cause**	**Slovakia**			**Slovenia**			**Spain**			**Sweden**			**United Kingdom**		
	**DALY rate**	**95%UI**	**(%)**	**DALY rate**	**95%UI**	**(%)**	**DALY rate**	**95%UI**	**(%)**	**DALY rate**	**95%UI**	**(%)**	**DALY rate**	**95%UI**	**(%)**
**NCDs**	**18,755**	**(15,624–22,338)**	**(100)**	**15,164**	**(12,511–18,272)**	**(100)**	**15,454**	**(13,046–18,341)**	**(100)**	**15,351**	**(12,978–18,096)**	**(100)**	**18,001**	**(15,317–21,079)**	**(100)**
**CVDs**	5,134	(4,207–6,179)	(27.4)	2,546	(2,068–3,143)	(16.8)	1,834	(1,699–1,958)	(11.9)	2,329	(2,149–2,483)	15.2)	2,362	(2,216–2,477)	(13.1)
**Chronic respiratory diseases**	476	(395–569)	(2.5)	505	(407–613)	(3.3)	752	(649–863)	(4.9)	786	(634–963)	(5.1)	1,187	(1,004–1,392)	(6.6)
**Diabetes and kidney diseases**	848	(666–1,049)	(4.5)	697	(516–888)	(4.6)	789	(608–999)	(5.1)	615	(502–746)	(4.0)	780	(592–993)	(4.3)
**Digestive diseases**	1,242	(996–1,555)	(6.6)	864	(697–1,078)	(5.7)	551	(500–614)	(3.6)	466	(410–535)	(3.0)	889	(794–1,008)	(4.9)
**Mental disorders**	1,373	(1,015–1,817)	(7.3)	1,462	(1,071–1,929)	(9.6)	2,192	(1,614–2,905)	(14.2)	2,017	(1,474–2,653)	(13.1)	1,959	(1,437–2,590)	(10.9)
**Musculoskeletal disorders**	1,541	(1,099–2,049)	(8.2)	1,521	(1,081–2,033)	(10.0)	1,831	(1,298–2,442)	(11.8)	2,036	(1,472–2,692)	(13.3)	2,314	(1,671–3,050)	(12.9)
**Neoplasms**	3,762	(2,974–4,712)	(20.1)	3,341	(2,615–4,278)	(22.0)	2,977	(2,820–3,120)	(19.3)	2,672	(2,550–2,783)	(17.4)	3,302	(3,164–3,403)	(18.3)
**Neurological disorders**	1,281	(739–2,049)	(6.8)	1,249	(727–2,025)	(8.2)	1,350	(700–2,272)	(8.7)	1,321	(692–2,210)	(8.6)	1,434	(826–2,318)	(8.0)
**Sense organ diseases**	606	(400–878)	(3.2)	568	(377–825)	(3.7)	538	(370–749)	(3.5)	340	(227–487)	(2.2)	503	(342–711)	(2.8)
**Skin and subcutaneous diseases**	451	(296–662)	(2.4)	451	(297–665)	(3.0)	698	(481–986)	(4.5)	692	(469–985)	(4.5)	736	(505–1,043)	(4.1)
**Substance use disorders**	477	(356–616)	(2.5)	621	(490–775)	(4.1)	469	(354–605)	(3.0)	692	(569–818)	(4.5)	924	(730–1,136)	(5.1)
**Other NCDs**	1,564	(1,204–2,023)	(8.3)	1,337	(997–1,772)	(8.8)	1,472	(1,129–1,900)	(9.5)	1,386	(1,048–1,799)	(9.0)	1,609	(1,254–2,037)	(8.9)

*Legend*: *%* percentage, *UI* Uncertainty Interval, *NCDs* noncommunicable diseases, and *CVDs* cardiovascular diseases

#### Age-standardized level 1 NCDs DALY rate ratios by countries and sex, 2019

**Fig. 1:**
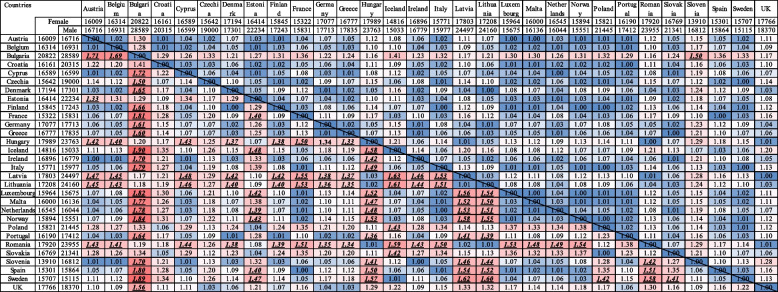
Ratio of age-standardized level 1 NCDs DALY rates for EEA Member States by sex, 2019. Legend: blue color represents ratios closer to 1 (equality), and red color represents ratios further from 1 (inequality). Ratios in bold and underlined represent statistical difference between DALY rate of compared countries (95% UI not overlapping). Ratios for females are above the diagonal line, and for males are under the line

The ratio of age-standardized level 1 NCDs DALY rate across countries in 2019 was close to 1.00, suggesting DALY rate across countries are close to equality (Fig. 1). It reached a maximum of 1.90 (statistically significant difference) between Bulgaria and Iceland for males. In other words, the NCDs DALY rate for males in Bulgaria was, on average, 1.9 times higher than the rate in Iceland. Overall, in males the ratio was higher in comparison to females, demonstrating greater cross-country inequalities in DALY rate for males. Compared with most other countries, the following eastern european countries had high NCD DALY rate ratios for males: Bulgaria, Estonia, Hungary, Latvia, Lithuania, Poland, Romania and Slovakia. A few ratios reached a ratio of 1.00, suggesting total equality in DALY rates between two countries: Denmark/Finland, Ireland/Austria, Spain/France, Ireland/Slovenia, Italy/Netherlands, and Poland/Slovakia.

The NCD DALY rate ratios in females reached a maximum of 1.50 between Slovenia and Bulgaria, which was the only statistically significant difference for females. In fact, Bulgaria – even in females – demonstrated the highest inequality in comparison to almost all the other EEA countries. Many comparisons between countries were reached a 1.00 ratio, such as: Italy/Finland, Lithuania/Denmark, Austria/Luxembourg, Austria/Malta, Malta/Luxembourg, UK/Latvia, Spain/France and others.

### Changes in NCDs DALY rate between 1990 and 2019

#### Age-standardized level 1 DALY rates by countries and sex between 1990 and 2019

**Fig. 2:**
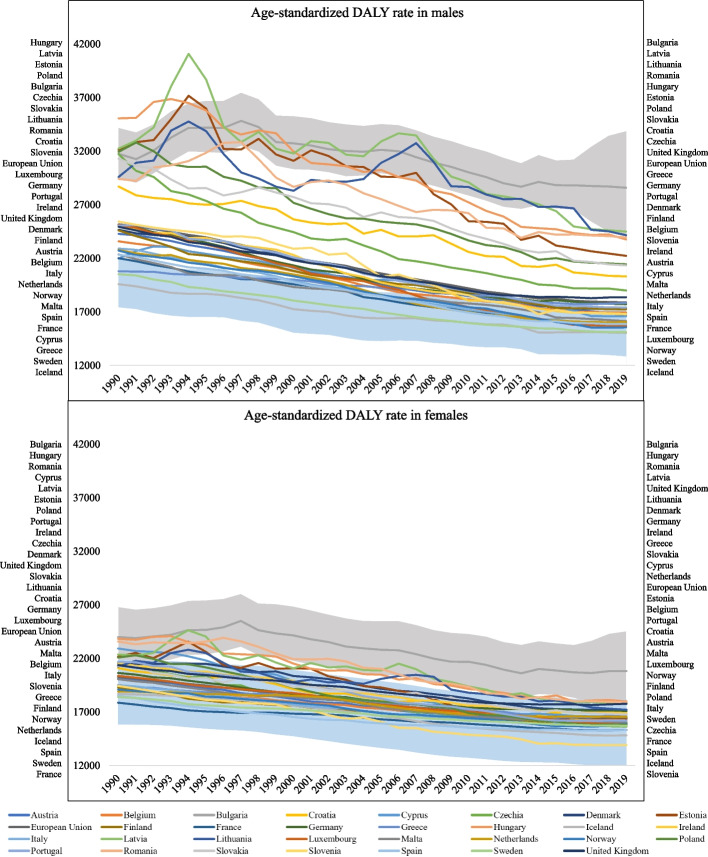
Age-standardized level 1 NCDs DALY rates by EEA Member States and sex, 1990–2019. Legend: UI 95% for the overall highest (Bulgaria) and lowest (Iceland) DALY rate are shown in grey and blue shaded band, respectively. The order of the country lines within the graphs corresponds to the order of the country labels next to each graph

Figure 2 shows NCD DALY rate per 100,000 population progression for each included country over time for males and females, from 1990 to 2019. There was an overall declining trend—the NCDs DALY rate gradually decreased in all countries over the 30-year follow-up period.

For the male population, a distinct differentiation between countries exhibiting elevated DALY rates and those presenting lower values in 1990 can be observed. The countries with a high DALY rate were Hungary with 35,066 (95% UI: 33,073 to 37,161) and Bulgaria with 31,747 (95% UI: 29,430 to 34,157). Countries with a low DALY rate ranged from 25,431 (95% UI: 21,152 to 30,611) in Slovenia to 19,589 (95% UI: 17,405 to 21,931) in Iceland. Comparison of DALY rates among the female population shows a more homogenous pattern across the member states of EU, confirmed by the overlapping 95% UI. In 1990, the highest DALY rate was between Bulgaria with 23,997 (95% UI: 21,538 to 26,836) to Romania with 23,579 (95% UI: 21,253 to 26,158). The lowest DALY rate was observed for France with 17,868 (95% UI: 14,979 to 21,155).

In 1994, the highest DALY rate for both males and females were found in Latvia, Estonia and Lithuania. From 1990 to 1997, NCDs DALY rate decreased in most countries, except for Bulgaria. In 2007, for males similarly to 1994, new peaks were observed in Latvia, Estonia and Lithuania. For the females in 2007, the peak values were in Latvia, Lithuania and in Hungary.

At the end of the study period, in 2019, most countries were in the range of 28,589/15,033 DALYs for males (Bulgaria/Iceland) and 20,822/13,910 DALYs for females (Bulgaria/Slovenia) – both with statistical difference confirmed by the not overlapped 95% UI. Bulgaria consistently maintained the highest DALY rate ratios for both sexes, surpassing other countries by a significant margin.

YLL rates also decreased in all countries and showed a high degree of similarity with the DALYs, except for the 95% UI, which was narrower. On the contrary, YLD values in both country groups were close to the plateau, for both sexes. The 95% UI for YLD was very wide for both sexes, indicating that there is little statistical difference in YLD rates between countries (Additional file 1).

#### Annual rate of change in age-standardized NCDs DALY rates

**Fig. 3:**
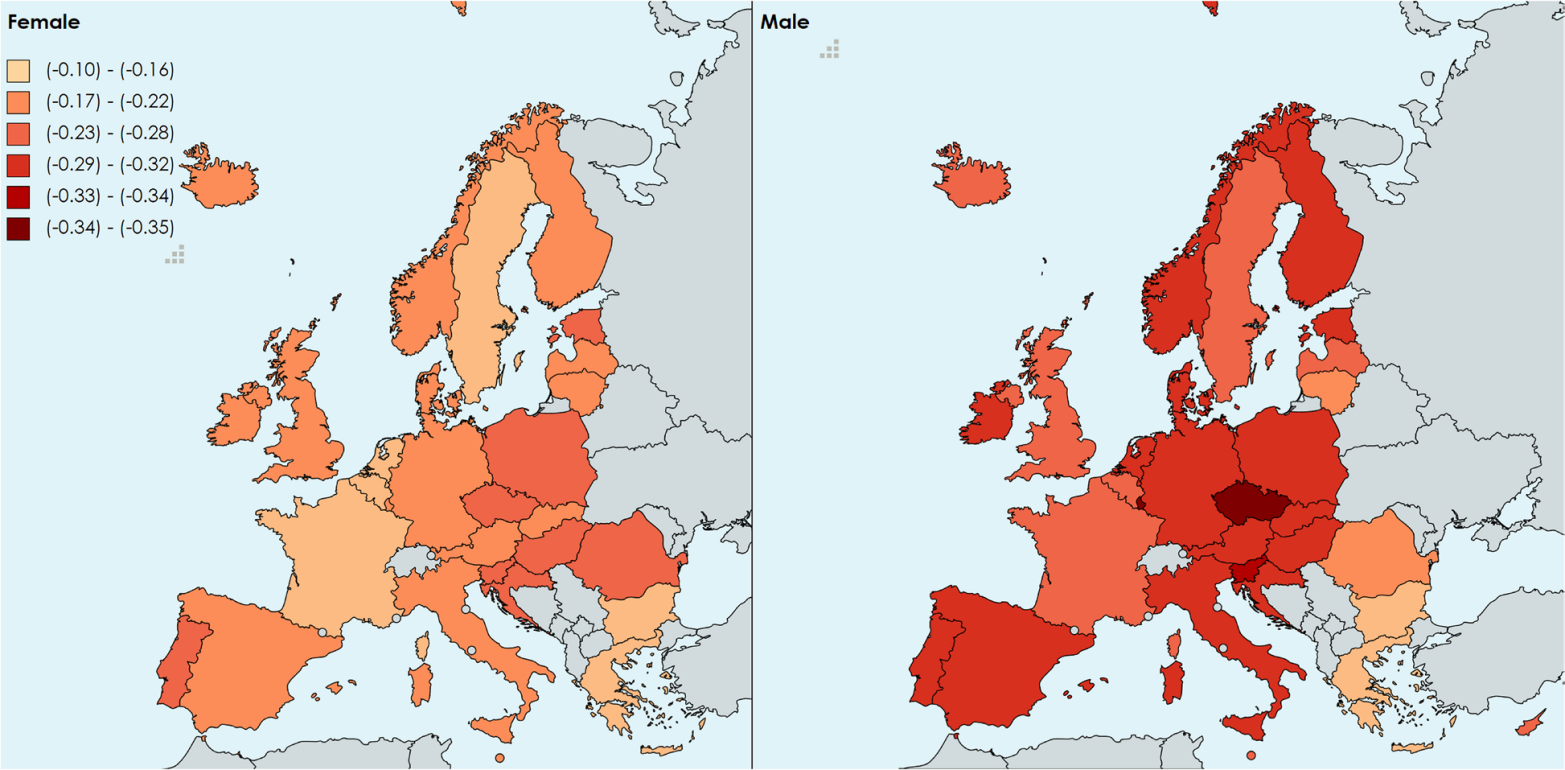
Age-standardized level 1 NCDs DALY annual rate of change, 1990–2019

Figure 3 shows that the level of change in females ranged from -0.12 (95% UI: -0.10 to -0.15) in the Netherlands to -0.28 (95% UI: -0.17 to -0.40) in Slovenia and (95% UI: -0.21 to -0.35) Poland. The change was larger in males, ranging from -0.10 (95% UI: 0.06 to -0.23) in Bulgaria to -0.40 (95% UI: -0.32 to -0.47) in Czechia.

For females, countries with the lowest rates ranged from -0.27 and -0.28 in (Poland, Slovakia, Cyprus and Czechia). Countries with the highest rates of change varied from -0.15 to -0.12 (Sweden, France, Greece, Bulgaria and the Netherlands).

Overall, males had larger reductions in the NCD DALY rates, since 18 countries ranged between -0.29 to -0.40, compared to the lowest value for females of -0.28. The lowest rates of change for males were observed in Czechia, Luxembourg, Slovenia, from -0.34 to -0.40. Whereas the countries with the smaller average reduction in DALY rates (between -0.10 and -0.18) were Bulgaria, Greece and Lithuania. The level 2 NCDs annual rate of change of DALY rates by country and sex are presented in the Additional file 2, which focuses on 5 diseases with the highest DALY rate ratio: CVD, chronic respiratory diseases, diabetes and kidney diseases, digestive diseases and substance use disorders.

### NCDs DALYs rate ratios by level 2 NCD cause of disease

**Fig. 4:**
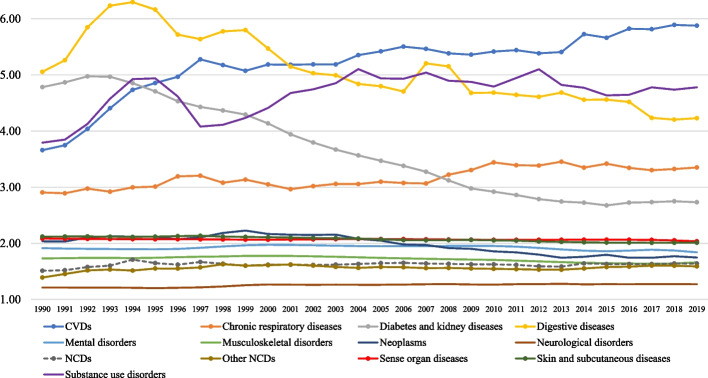
Age-standardized DALY rate ratio by level 2 cause of disease between 1990 and 2019. Legend: Ratio is calculated for each NCD at level 2 by dividing the highest-ranking DALY rate by the lowest-ranking per year of the study period. NCDs: noncommunicable diseases and CVDs: cardiovascular diseases

The age-standardized level 2 NCD DALY rate ratio by year between 1990 and 2019 is presented in Fig. 4.

From 1990 through 2019, five NCDs had consistent high DALY rate ratios of 2.68 or higher, namely digestive diseases, diabetes and kidney diseases, substance use disorders, CVDs, and chronic respiratory diseases. For CVDs, the DALY ratio increased from 3.66 in 1990 to 5.88 in 2019, whereas for digestive diseases and diabetes and kidney diseases a decrease in DALY rate ratio was observed between 1990 and 2019.

For NCDs, musculoskeletal disorders, mental disorders, neoplasms, and sense organ diseases the DALY rate ratio was consistently lower than 2.23 between 1990 and 2019, with slight changes in ranking of the diseases according to DALY rate ratio over time.

### Assessing health inequalities in NCDs by using Gini coefficient and Slope Index of Inequality

**Fig. 5:**
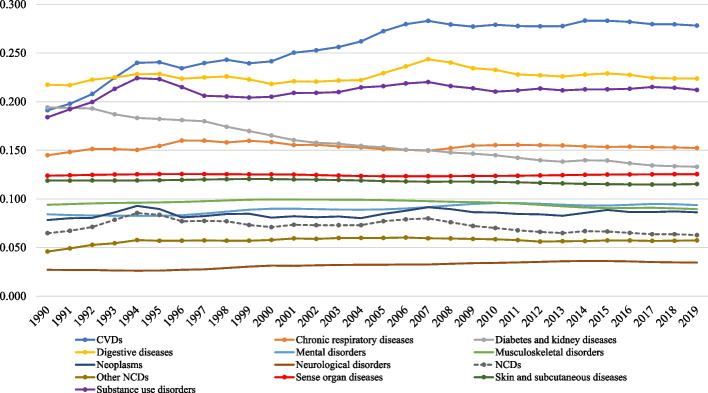
Gini coefficient of age-standardized NCDs DALY rate in EEA Member States, 1990–2019. Legend: NCDs: non-communicable diseases and CVDs: cardiovascular diseases

For level 1 NCDs, low inequalities according to the GC between countries were found. While the lowest GC were found between 2017 and 2019, from 0.064 (95% CI: 0.044 to 0.083) to 0.063 (95% CI: 0.040 0.086), the highest GC was observed in 1994, 1995, and 2007, which were 0.085 (95% CI: 0.065 to 0.106), 0.084 (95% CI: 0.067 to 0.101), and 0.080 (95% CI: 0.052 to 0.108), respectively; as shown in Fig. 5.

The highest GCs were observed for CVD, chronic respiratory diseases, diabetes and kidney diseases, digestive diseases, and substance use disorders. CVD showed an increasing GC value, from 0.191 in 1990 to 0.278 (95% CI: 0.214 to 0.342) in 2019. A similar pattern was found for substance use disorders, although it was lower for the same period: 0.184 (95% CI: 0.140 to 0.228) and 0.212 (95% CI: 0.161 to 0.263). A more stable, but still high, GC was found for digestive diseases, in which it ranged from 0.217 (95% CI: 0.162 to 0.273) to 0.224 (95% CI: 0.185 to 0.262). Even though diabetes and kidney diseases were the second highest level 2 NCDs in 1990, it progressively decreased to the fifth position in 2019, going from 0.194 (95% CI: 0.137 to 0.251) to 0.133 (95% CI: 0.106 to 0.160). Chronic respiratory diseases were in a range between 1990 with 0.145 and 2019 with 0.152 (95% CI: 0.113 to 0.191). Mental disorders, musculoskeletal disorders, neoplasms, sense organ diseases, skin and subcutaneous diseases, neurological disorders and other NCDs, had lower values, between 0.026 (95% CI: 0.020 to 0.032) to 0.125 (95% CI: 0.112 to 0.139) throughout the 30 years’ follow-up period.

**Fig. 6:**
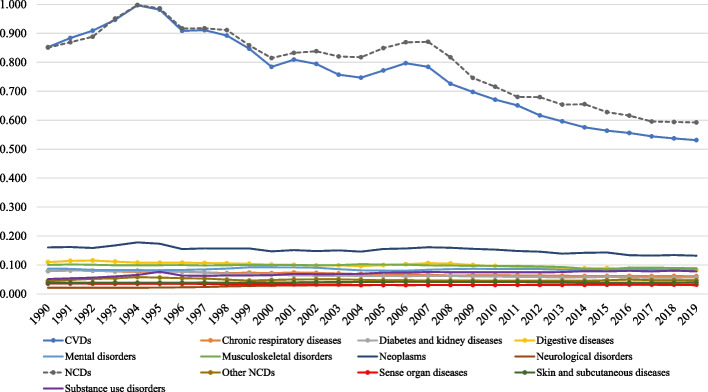
Slope Index of Inequality of age-standardized NCDs DALY rate in EEA Member States, 1990–2019. Legend: NCDs: non-communicable diseases and CVDs: cardiovascular diseases

Figure 6 and Additional file 3 show that the SII was highest for level 1 NCDs, which was 0.851 (95% CI: 0.730–0.972) in 1990 and 0.592 (95% CI: 0.470–0.715) in 2019, with two peaks in 1994 and 2007, 0.951 (95% CI: 0.781–1.121) and 0.871 (95% CI: 0.679–1.063), respectively.

CVDs followed the level 1 NCDs pattern closely in 1990, 1994, 2007, and 2019: 0.852 (0.708 to 0.997), 0.997 (0.776 to 1.245), 0.784 (0.569 to 1.000), and 0.531 (0.381 to 0.681), respectively. Another level 2 NCD with the elevated SII was neoplasms, however, it showed a much lower and steady inequality trend across the years, from 0.161 (0.136 to 0.185) in 1990 to 0.132 (0.111 to 0.153) in 2019. For a group of level 2 diseases, the SII was very close to zero over the follow-up years, ranging from 0.021 (neurological disorders in 1990) to 0.115 (digestive diseases in 1992) – these were chronic respiratory diseases, diabetes and kidney diseases, digestive diseases, mental disorders, musculoskeletal disorders, neurological disorders, substance use disorders, other NCDs, sense organ diseases, and skin and subcutaneous diseases.

## Discussion

This study describes DALY rates of NCDs as a snapshot in 2019, and their trends over three decades; and also presents DALY rate ratios, GC and SII to express health inequalities due to NCDs in EEA countries.

A progressive decrease in the age-standardized NCDs DALY rate for 30 EEA countries from 1990 to 2019 for both males and females was observed. Most countries remained similarly ranked compared to other countries over the years, showing a proportional decrease in DALYs in 2019 compared to 1990. Furthermore, the pace of decrease in DALY rate was similar across countries, the ratio of DALY rate across country-pairs remained similar, and the overall NCDs DALY rate decreased in the EEA region. Thus, despite a general improvement in the burden of disease across all countries from 1990 to 2019, and despite a narrowing of income inequalities between countries in the same period [59], the inequalities in the disease burden between the EEA countries has remained.

The progressive decrease of NCDs DALY rate in western european countries, such as Austria, Belgium, Denmark, and Iceland, were far more steady and lower during the follow up period. Whereas, for the EU-11 countries, which include Bulgaria, Croatia, Czechia, Estonia, Hungary, Latvia, Lithuania, Poland, Romania, Slovenia and Slovakia, a different trend was observed. Bulgaria, Latvia, Lithuania, and Estonia showed a fluctuating DALY rate, with peaks in 1994 and 2007. The significant upsurge in the DALY rate within those countries, notably Bulgaria, during the period from 1990 to approximately 1997, is noteworthy, particularly with regard to the male population. Subsequently, these countries show a comparable level of reduction in the DALY rate as observed in other countries. However, they initiated their decrease trajectory from a markedly higher rate, which may indicate that those countries experienced events leading to the decrease of DALY rate for NCDs. The peak in 1994 may be explained by the socio-economic crisis following the dissolution of the Soviet Union and the collapse of the communist governments, as well as the transformation of the health systems, which was reflected in greater inequalities and an increase in the mortality rate from NCDs in the countries of the region [60]. In three Baltic countries—Latvia, Lithuania, and Estonia – the NCDs DALY rate increased dramatically in 1991, peaked in 1994 and then returned to a lower level in 1996. The increase in NCD DALY rates in Bulgaria, and others, for the period 1990 to 1997 reflects the economic crisis observed in the period, peaking with hyperinflation at the end of 1996 and the beginning of 1997. These factors suggest that post-socialist countries might have suffered an economic and political crisis, in which the impacts on health appeared at different times.

Another peak in NCDs DALY rate increase started in 2004, when Latvia, Lithuania, and Estonia joined the EU, which might be explained by cross-border migration of youth [61]. This trend peaked in 2007 and then started to fall back to a downward trend for NCDs—showing that the health status deteriorated after the enlargement of 2004 and 2007 for some new countries [62]. However, other studies have found no convincing evidence that EU accession has affected the process of mortality convergence between the pre-2004 and post-2004 Member States [61].

The Great Recession in 2008 had a significant impact on the healthcare system of many countries in the EEA [63]. Governments have responded to the economic crisis by implementing financial austerity measures such as curbing healthcare spending and access to services. Widening health inequalities in the EU-27 were a major consequence of the Great Recession [64]. The economic crisis has also led to a decline in the quality of life and an increase in unemployment, as well as an increase in poverty, anxiety, suicide, alcoholism and malnutrition [63, 65]. All these adverse changes in socio-economic factors might had influenced the epidemiological and economic burden of NCDs [66].

However, establishing a direct temporal link between adverse socioeconomic changes as determinants and changes in DALYs in a given year can be very inaccurate, as most NCDs have a long latency period [67]. Moreover, differences between countries in physical inactivity and obesity may have led to greater inequalities in NCDs in Europe, as these two risk factors appear to have increased between 2004 and 2015, but some of these risk factors may take decades to have an impact on the YLL rates for most NCDs [68, 69]. Health inequalities between countries are influenced by many factors, beyond those listed above, such as differences between countries in the number of doctors and nurses per 100,000 people, in health expenditure, in national health promotion measures [70–72].

For some NCDs at level 2, the NCD DALY rate ratios were extremely high when a country with the highest DALY rate was compared with the lowest ranking country. The ratios for digestive diseases, diabetes and kidney diseases, substance use disorders, CVDs, and chronic respiratory diseases ranged from 2.73 (diabetes and kidney diseases in 2019) to 6.29 (digestive diseases in 1996). The GC coefficient confirms that the inequalities between all included countries are higher for these diseases in comparison to the others. This may draw attention to the need for targeted, disease-specific prevention programs in the EU. Most of these diseases are associated with an unhealthy lifestyle, distinguished by poor dietary patterns, harmful alcohol intake, and tobacco use. Nonetheless, these risk factors can be modified by means of lifestyle adjustments. Consequently, it is crucial for countries that demonstrate elevated DALY rates to intensify their preventive measures against these risk factors. For instance, Japan has one of the lowest NCD DALY rate in the world, and the country has very stablished and comprehensive public health policies, including active lifestyle promotion, healthy eating initiatives, and strict tobacco control measures [73, 74]. It is important to note that according to the SII analysis, the significant enhancement in the health-related to NCDs in EEA countries during the last three decades, is primarily attributed to the contribution of CVDs in absolute terms.

The observed decrease in health inequalities for diabetes and kidney disease is present in both analyses: pairs of countries (ratio) and all countries together (GC). In contrast, CVD exhibits an increase in both health inequalities analysis, which can be attributed to some countries having achieved significant improvements in CVD-related DALY values due to successful prevention and treatment measures, while others have experienced limited success, leading to a modest decline or stabilization in their DALY trends. The reduction in mortality from CVD can be attributed to advancements in prevention and treatment approaches, as well as favorable changes in risk factors such as smoking, blood pressure, and cholesterol levels [75]. The contrasting trends between diabetes, kidney disease and CVD may also be due to differences in the weight of risk factors associated with each NCD. The major risk factors for diabetes and kidney disease include physical inactivity and obesity. The efforts made in these areas in Europe over the past three decades have disappointingly failed [76], and on a global scale, there is not much success to report. Although the burden of CVD has declined more than that of diabetes in Europe during this period [77], both diseases persist as significant public health challenges necessitating effective prevention and treatment strategies [76, 78].

According to WHO Global NCD Action Plan 2013–2030, the recommended interventions are to reduce the risk factors for NCDs (tobacco use, harmful use of alcohol, unhealthy diets, physical inactivity) and to enable health systems to respond to the health needs of people living with or at risk of the major NCDs (cardiovascular diseases, cancers, diabetes, chronic respiratory diseases). Among prevention policies, only tobacco control has seen systematic international action. In addition to the WHO Framework Convention on Tobacco Control (FCTC), which entered into force in 2005, the EU legislative framework for tobacco control has been developed. The instruments of the legal framework include marketing rules, educational campaigns, pharmacotherapy and tobacco taxation policy. Smokers are more prevalent in countries with low levels of tobacco control enforcement [79]. In the EU-27, Member States that implement more measures to reduce smoking prevalence and encourage smoking cessation report more people quitting and lower rates of smoking [80].

However, there are no enforceable international/EU treaties or other legal instruments for the control of other behavioral risk factors. For example, international efforts to regulate alcohol consumption have been less successful than in the case of tobacco [81]. Moreover, the effectiveness of policies to prevent or reduce harmful alcohol consumption has not been adequately assessed yet [82, 83]. In the EU, some fragmented interventions and policies are mainly based on implementing control over alcohol availability, pricing policies, educational interventions, screening risk drinkers, and brief intervention [84–86].

Policies can contribute to reducing health inequalities and creating the conditions for a healthy life for all. The WHO Health Equity Policy Tool (2019) connects five essential conditions for a healthy life (health services, income security and social protection, living conditions, social and human capital, and employment and working conditions) to policy areas for which evidence for action is strong. To reduce inequalities in NCDs, a network of policies is needed, including environmental measures (housing, transport) and measures to address commercial determinants of health (marketing, taxing unhealthy products, and promoting fruit and vegetable production, removal of unhealthy products rich in sugar, salt and fat from automatic vending machines, especially in school and work premises). Policies should be focused on detecting, monitoring, and preventing physiological and behavioral factors for NCDs, with a specific focus in lower socioeconomic classes [87].

In European countries, sex inequalities in health are apparent. Disparities in DALY were greater for males than for females, in all the absolute and relative analyses we conducted. However, as depicted in the maps, the annual rate of change between 1990 and 2019 was much lower for males, implying that the DALY rate for NCDs have declined substantially over the 30-year period.

The high equality level in the DALY rate ratio of the female population can be explained by both countries having similar DALY rate. However, this does not necessarily reflect low DALY rate, since the compared countries can present similarly high rates and thus the ratio will be close to 1, demonstrating that there is a ceiling effect in the ratio calculation that may mask variation. Also, NCDs YLL rates for males are much higher than the YLL for females. In opposite, YLD rates for females was higher than YLD for males. This inverse association of higher YLL rates for males and higher YLD rates for female is probably related to differences in lifestyle choices, risk behavior and access to healthcare [88]. In Europe, males are more prone to adopt unhealthy behavior (excessive alcohol and tobacco consumption) and hazardous jobs (exposure of harmful substances and dangerous workspaces) [89, 90]. In addition, as females are more aware of their health status and have access to preventive health services, they attend more screening programs and seek health care when they have symptoms of NCDs, leading to higher life expectancy [88]. It could also be assumed that the observed trends in DALY rate from 1990 to 2019 for all NCDs were primarily determined by YLL rates and that YLD rates contributed less.

As compared to previous studies on health inequalities in Europe, the main strengths of this study are longer follow-up period, better data availability, use of age-standardization measures, and inclusion of all EEA countries [50, 88, 91]. The use of age-standardization of DALY rate elicited from the GBD study allows a harmonized and validated measure of both NCD mortality and disability. Additionally, the use of age-standardized rates allowed us to compare data between many countries, across 30 years of follow up, with different economic backgrounds and age profiles. We also aimed to diminish bias by calculating inequality not only using ratios, but also the SII and GC, which provide both an absolute and relative depiction of inequality. Also, analyzing inequalities at level 2 NCDs provided unique results regarding the differences in inequalities in each disease group. As this is an important variable in terms of political and economic resources, it was also appropriate to compare sex inequalities. The objective underlying the provision of detailed tables and graphs in this study was to facilitate the analysis and comprehension of health inequalities between countries and within the EEA. Through the inclusion of highly comprehensive tables and graphs, we achieved our aim of providing a broad understanding of the ratios, not solely pertaining to the comparison between the country exhibiting the highest and lowest DALY rates, but also encompassing any pair of countries examined in our sample. Moreover, the meticulous information concerning the analysis of international disparities includes not only comparisons between individual country pairs but also encompasses the entire sample of countries included in the research.

The present study has some limitations, many of them are intrinsic limitations of the GBD study—these can be found elsewhere [47]. The uncertainty of estimates due to limited data, possibility of inaccurate determination and classification of non-fatal conditions, and lack of primary data (particularly for morbidity data). However, a few limitations are unique to our study. One of them is the determination of inequality according to DALY rate over a 30-year period, which included the ratio only between extremes: highest ranking country/lowest ranking country. However, the GC, SII, and the contingency table were included to mitigate this limitation by including all the countries and/or all the years in the analysis. The method of employing statistical significance analysis as the overlapping 95% UI demonstrates effectiveness in detecting statistical significance whereby non-overlapping intervals indicate significance. However, this approach may not consistently provide reliable outcomes in the opposite direction, when slightly overlapping 95% UI may still yield statistical significance. Despite this limitation, the employment of the 95% UI remains preferable over the 95% CI since it incorporates model uncertainty, thus rendering it a more meaningful measure. Moreover, relying on Poisson regression to produce p-values and 95% CIs would indicate statistical significance for almost all ratios, given its exclusive focus on DALY rates. In contrast, the adoption of the 95% UI overlapping rule is a more significant measure, particularly as the GBD 2019 study incorporates various other epidemiological metrics into its calculation of UI. The GBD database does not present estimates for microstate Liechtenstein, which was the only country excluded from our analysis of EEA member states. An additional limitation is the assumption of linear change in the annual change estimates, since our analysis showed that, for some former Soviet countries, this assumption might be incorrect. However, this was mitigated by depicting the DALY rate of change over each year for each country visually. The data quality is, furthermore, diverse. Depending on the country, the GBD uses Bayesian methods to try and overcome this. Non-fatal data can differ dramatically between countries; for this reason, the Bayesian models may lead to incorrect estimates based on the surrounding countries. Also, inequalities exist both within and between countries, but the present study only compared inequalities between countries.

## Conclusions

In conclusion, our study shows that the NCDs with higher level of inequality across countries of EEA are digestive diseases, diabetes and kidney diseases, substance use disorders, CVDs, and chronic respiratory diseases. However, the GC analysis showed that the level 1 NCDs DALYs inequality within all included countries is narrow. This study also highlighted that the DALY rate from NCDs decreased between 1990 and 2019 in all the 30 EEA member states. The rate of change, however, varied between males and females and across regions and was larger for males and in Central European countries. Underlying social inequalities could be reduced through the right selection of policies. In addition to policies that target modifiable risk factors, emphasis should also be placed on health inequalities between EEA Member States that may also be due to the heterogeneity of social factors.

## Supplementary Information

**Additional file 1:** Figure: Age-standardized NCDs YLLs and YLDs rate for EEA Member States by sex, 1990–2019. Legend: UI 95% for the overall highest (Bulgaria) and lowest (Iceland) DALYs rate are shown in grey and blue shaded band, respectively.

**Additional file 2:** For females, the UK had the highest annual rate of change, followed by Estonia and Finland. The countries with the lowest annual rates of change for females were Cyprus, Romania and Italy. In the EEA, the lowest annual rate of change for females was reported for CVDs at -0.54, followed by DDs at -0.26 and CRDs at -0.16. For males, the highest annual rates of change in DALYs were in Estonia, followed by the UK, and the lowest in Italy, Spain and Portugal. For males in the EEA, the lowest annual disease-specific rate of change was -0.55 for CVDs, followed by CRDs -0.39 and then DDs -0.32. Figure: Age-standardized level 2 NCDs DALY annual rate of change by EEA Member States, 1990–2019. Legend: CVDs: cardiovascular diseases.

**Additional file 3:** Figure: Slope Index of Inequality of age-standardized level 1 and 2 NCDs DALYs rates, 1990–2019. Legend: CVDs: cardiovascular diseases; CI: Confidence Interval; NCDs: non-communicable diseases; Coef.: Coefficient.

## Data Availability

Data are available in a public, open access repository (ghdx.healthdata.org). The data that support the findings of this study are available from the corresponding author upon reasonable request.

## Abbreviations

CVDs: Cardiovascular diseases
DALYs: Disability-adjusted Life Years
DM: Diabetes Mellitus
EEA: European Economic Area
EU: European Union
GBD: Global Burden of Disease
GC: Gini Coefficient
GDP: Gross Domestic Product
NCDs: Non-communicable Diseases
SDG: Sustainable Development Goal
SII: Slope Index of Inequality
UI: Uncertainty Interval
UK: United Kingdom
UN: United Nations
WHO: World Health Organization
YLD: Years Lived with Disability
YLL: Years of Life Lost

## References

[1] Global Burden of Disease Collaborative Network, Global Burden of Disease Study 2019 (GBD 2019) Results (Institute for Health Metrics and Evaluation – IHME) https://vizhub.healthdata.org/gbd-results/.

[2] NCD Data Portal - World Health Organization. https://ncdportal.org/Indicators.

[3] Di Cesare M (2019). Global trends of chronic non-communicable diseases risk factors. Eur J Public Health.

[4] Budreviciute A, Damiati S, Sabir DK, Onder K, Schuller-Goetzburg P, Plakys G, et al. Management and prevention strategies for non-communicable diseases (NCDs) and their risk factors. Front Public Health. 2020;8:788. 10.3389/fpubh.2020.574111 PMC7726193 33324597

[5] Sommer I, Griebler U, Mahlknecht P, Thaler K, Bouskill K, Gartlehner G, Mendis S (2015). Socioeconomic inequalities in non-communicable diseases and their risk factors: an overview of systematic reviews. BMC Public Health.

[6] Lago-Peñas S, Rivera B, Cantarero D, Casal B, Pascual M, Blázquez-Fernández C, Reyes F (2021). The impact of socioeconomic position on non-communicable diseases: what do we know about it?. Perspect Public Health.

[7] Hosseinpoor AR, Bergen N, Mendis S, Harper S, Verdes E, Kunst A, Chatterji S (2012). Socioeconomic inequality in the prevalence of noncommunicable diseases in low-and middle-income countries: results from the World Health Survey. BMC Public Health.

[8] Johansson I, Norhammar A (2021). Diabetes and heart failure notions from epidemiology including patterns in low-, middle-and high-income countries. Diabetes Res Clin Pract.

[9] Communication from the commission to the European parliament, the council, the European economic and social committee and the committee of the regions: solidarity in health: reducing health inequalities in the EU. vol. 567 final: office for official publications of the European communities. European Comission. 2009. https://eur-lex.europa.eu/legal-content/EN/TXT/?uri=celex%3A52009DC0567. Accessed 15 Apr 2023.

[10] McNamara CL, Balaj M, Thomson KH, Eikemo TA, Solheim EF, Bambra C (2017). The socioeconomic distribution of non-communicable diseases in Europe: findings from the European Social Survey (2014) special module on the social determinants of health. Eur J Public Health.

[11] Whitehead M, Dahlgren G (2007). European strategies for tackling social inequities in health: leveling up part 2. Studies on social and economic determinants of population health, N 3.

[12] McCartney G, Popham F, McMaster R, Cumbers A (2019). Defining health and health inequalities. Public Health.

[13] Mackenbach JP, Meerding WJ, Kunst AE (2011). Economic costs of health inequalities in the European Union. J Epidemiol Community Health.

[14] Mackenbach JP, Valverde JR, Artnik B, Bopp M, Brønnum-Hansen H, Deboosere P, Kalediene R, Kovács K, Leinsalu M, Martikainen P (2018). Trends in health inequalities in 27 European countries. Proc Natl Acad Sci.

[15] Jutz R (2020). Health inequalities in Eastern Europe. Does the role of the welfare regime differ from Western Europe?. Soc Sci Med.

[16] Thomson KH, Renneberg A-C, McNamara CL, Akhter N, Reibling N, Bambra C (2017). Regional inequalities in self-reported conditions and non-communicable diseases in European countries: findings from the European Social Survey (2014) special module on the social determinants of health. Eur J Public Health.

[17] Zatonski W (2007). The east-west health gap in Europe—what are the causes?. Eur J Public Health.

[18] Haagsma JA, Charalampous P, Ariani F, Gallay A, MoesgaardIburg K, Nena E, Ngwa CH, Rommel A, Zelviene A, Abegaz KH (2022). The burden of injury in Central, Eastern, and Western European sub-region: a systematic analysis from the Global Burden of Disease 2019 Study. Arch Public Health.

[19] Weziak-Bialowolska D (2014). Health conditions in regions of Eastern and Western Europe. Int J Public Health.

[20] Carriazo S, Ortiz A. European East–West divide in kidney disease: the need to understand the drivers of chronic kidney disease outcomes. Clin Kidney J. 2021;14:1–4. 10.1093/ckj/sfaa217 PMC7857834 33564399

[21] Richardson EA, Pearce J, Mitchell R, Shortt NK, Tunstall H (2014). Have regional inequalities in life expectancy widened within the European Union between 1991 and 2008?. Eur J Public Health.

[22] Niessen LW, Mohan D, Akuoku JK, Mirelman AJ, Ahmed S, Koehlmoos TP, Trujillo A, Khan J, Peters DH (2018). Tackling socioeconomic inequalities and non-communicable diseases in low-income and middle-income countries under the Sustainable Development agenda. Lancet.

[23] Freisling H, Viallon V, Lennon H, Bagnardi V, Ricci C, Butterworth AS, Sweeting M, Muller D, Romieu I, Bazelle P (2020). Lifestyle factors and risk of multimorbidity of cancer and cardiometabolic diseases: a multinational cohort study. BMC Med.

[24] Mackenbach JP, Stirbu I (2008). Roskam A-JR, Schaap MM, Menvielle G, Leinsalu M, Kunst AE: Socioeconomic inequalities in health in 22 European countries. N Engl J Med.

[25] Gender and noncommunicable diseases in Europe: analysis of STEPS data. WHO/EURO:2020-1664-41415-56457. World Health Organization (WHO). Regional Office for Europe 2020.

[26] Eurobarometer S (2017). 458: attitudes of Europeans towards tobacco and electronic cigarettes.

[27] Global status report on alcohol and health 2018. Geneva: World Health Organization (WHO). 2019. https://www.who.int/publications/i/item/9789241565639. Accessed 10 Apr 2023.

[28] Papadaki A, Hondros G, Scott JA, Kapsokefalou M (2007). Eating habits of university students living at, or away from home in Greece. Appetite.

[29] Ferrant G, Pesando LM, Nowacka K (2014). Unpaid Care Work: The missing link in the analysis of gender gaps in labour outcomes.

[30] Communication from the commission to the European parliament, the council, the European economic and social committee and the committee of the regions: a union of equality: gender equality strategy 2020-2025. In towards a gender equal Europe Brussels: European union, vol. 152 final: office for official publications of the European communities. European comission. 2020. https://eur-lex.europa.eu/legal-content/EN/TXT/?uri=CELEX%3A52020DC0152. Accessed 15 Apr 2023.

[31] Healthier together. EU non-communicable diseases initiative. 1st ed. Luxembourg: Publications office of the European union. European Comission; 2022. p. 161.

[32] Fantom NJ, Serajuddin U. The world bank's classification of countries by income. Policy research working paper, no. WPS 7528 Washington, D.C. : world bank group. 2016. http://documents.worldbank.org/curated/en/408581467988942234/The-World-Banks-classification-of-countries-by-income. Accessed 09 Apr 2023.

[33] Prevention and control of noncommunicable diseases in the European Region: a progress report. WHO/EURO:2014-3441-43200-60519. World Health Organization (WHO). Regional Office for Europe. 2014.

[34] Hitiris T (1997). Health care expenditure and integration in the countries of the European Union. Appl Econ.

[35] Busse R, Wismar M, Berman PC. The European union and health services: the impact of the single European market on member states. Amsterdam: IOS Press; 2002.

[36] Nugent R, Bertram MY, Jan S, Niessen LW, Sassi F, Jamison DT, Pier EG, Beaglehole R (2018). Investing in non-communicable disease prevention and management to advance the Sustainable Development Goals. Lancet.

[37] Action plan for the prevention and control of noncommunicable diseases in the WHO European Region. WHO/EURO:2016-2582-42338-58618. World Health Organization (WHO). Regional Office for Europe; 2016.

[38] Singh Thakur J, Nangia R, Singh S (2021). Progress and challenges in achieving noncommunicable diseases targets for the sustainable development goals. FASEB Bioadv.

[39] Palmer K, Monaco A, Kivipelto M, Onder G, Maggi S, Michel J-P, Prieto R, Sykara G, Donde S (2020). The potential long-term impact of the COVID-19 outbreak on patients with non-communicable diseases in Europe: consequences for healthy ageing. Aging Clin Exp Res.

[40] Murray CJ, Lopez AD, Jamison DT (1994). The global burden of disease in 1990: summary results, sensitivity analysis and future directions. Bull World Health Organ.

[41] Lundkvist A, El-Khatib Z, Kalra N, Pantoja T, Leach-Kemon K, Gapp C, Kuchenmüller T (2021). Policy-makers' views on translating burden of disease estimates in health policies: bridging the gap through data visualization. Arch Public Health.

[42] Murray CJ, Lopez AD, Jamison DT. The global burden of disease in 1990: summary results, sensitivity analysis and future directions. Bull World Health Organ (WHO). 1994;72:495. PMC2486716 8062404

[43] Homedes N (1996). The disability-adjusted life year (DALY) definition, measurement and potential use.

[44] Martinez R, Lloyd-Sherlock P, Soliz P, Ebrahim S, Vega E, Ordunez P, McKee M (2020). Trends in premature avertable mortality from non-communicable diseases for 195 countries and territories, 1990–2017: a population-based study. Lancet Glob Health.

[45] Non-communicable diseases—level 1 cause. Global health metrics: the lancet. 2020. https://www.thelancet.com/gbd/summaries. Accessed 10 Apr 2023.

[46] Santos JV, Gorasso V, Souza J, Wyper GMA, Grant I, Pinheiro V, Viana J, Ricciardi W, Haagsma JA, Devleesschauwer B (2021). Risk factors and their contribution to population health in the European Union (EU-28) countries in 2007 and 2017. Eur J Pub Health.

[47] Vos T, Lim SS, Abbafati C, Abbas KM, Abbasi M, Abbasifard M, Abbasi-Kangevari M, Abbastabar H, Abd-Allah F, Abdelalim A (2020). Global burden of 369 diseases and injuries in 204 countries and territories, 1990–2019: a systematic analysis for the Global Burden of Disease Study 2019. The Lancet.

[48] Global Burden of Disease (GBD) Compare. https://vizhub.healthdata.org/gbd-compare/.

[49] Global Burden of Disease (GBD) Results. https://vizhub.healthdata.org/gbd-results/.

[50] Armocida B, Monasta L, Sawyer S, Bustreo F, Segafredo G, Castelpietra G, Ronfani L, Pasovic M, Hay S, Perel P, Beran D (2022). Burden of non-communicable diseases among adolescents aged 10–24 years in the EU, 1990–2019: a systematic analysis of the Global Burden of Diseases Study 2019. Lancet Child Adolesc Health.

[51] Cieza A, Causey K, Kamenov K, Hanson SW, Chatterji S, Vos T (2021). Global estimates of the need for rehabilitation based on the Global Burden of Disease study 2019: a systematic analysis for the Global Burden of Disease Study 2019. Lancet.

[52] GBD 2016 Parkinson's disease collaborators. Global, regional, and national burden of Parkinson's disease, 1990-2016: a systematic analysis for the global burden of dsease study 2016. Lancet Neurol. 2018;17:939–53. 10.1016/S1474-4422(18)30295-3 PMC6191528 30287051

[53] Schneider MC, Castillo-Salgado C, Bacallao J, Loyola E, Mujica OJ, Vidaurre M, Roca A (2002). Methods for measuring inequalities in health. Rev Panamericana de Salud Publica Pan Am J Public Health.

[54] Jenkins SP, INEQDECO (1999). Stata module to calculate inequality indices with decomposition by subgroup," Statistical Software Components S366002, Boston College Department of Economics, revised 15 Feb 2021.

[55] Steinbeis F, Gotham D, von Philipsborn P, Stratil JM (2019). Quantifying changes in global health inequality: the Gini and Slope Inequality Indices applied to the Global Burden of Disease data, 1990–2017. BMJ Glob Health.

[56] Microsoft Corporation, 2018. Microsoft Excel. 2018. Available at: https://office.microsoft.com/excel.

[57] StataCorp.  (2015). Stata Statistical Software: Release 14.

[58] https://www.mapchart.net/.

[59] Filauro GFaS (2021). Income inequality in the EU: General trends and policy implications.

[60] Peasey A, Bobak M, Kubinova R, Malyutina S, Pajak A, Tamosiunas A, Pikhart H, Nicholson A, Marmot M (2006). Determinants of cardiovascular disease and other non-communicable diseases in Central and Eastern Europe: rationale and design of the HAPIEE study. BMC Public Health.

[61] Hrzic R, Vogt T, Brand H, Janssen F (2021). The short-term effects of European integration on mortality convergence: a case study of European Union's 2004 Enlargement. Eur J Popul.

[62] Santos JV, Lobo M, Neiva RM, Viana J, Souza J, Dias CC, Cylus J, Ricciardi W, Freitas A (2020). European Union state of health from 1990 to 2017: time trends and its enlargements' effects. Int J Public Health.

[63] Baumbach A, Gulis G (2014). Impact of financial crisis on selected health outcomes in Europe. Eur J Public Health.

[64] Maynou L, Saez M (2016). Economic crisis and health inequalities: evidence from the European Union. Int J Equity Health.

[65] Margerison-Zilko C, Goldman-Mellor S, Falconi A, Downing J (2016). Health impacts of the great recession: a critical review. Curr Epidemiol Rep.

[66] Karanikolos M, Heino P, McKee M, Stuckler D, Legido-Quigley H (2016). Effects of the Global Financial Crisis on Health in High-Income Oecd Countries: A Narrative Review. Int J Health Serv.

[67] Barouki R, Gluckman PD, Grandjean P, Hanson M, Heindel JJ (2012). Developmental origins of non-communicable disease: implications for research and public health. Environ Health.

[68] Ekelund U, Ward HA, Norat T, Luan JA, May AM, Weiderpass E, Sharp SJ, Overvad K, Ostergaard JN, Tjonneland A (2015). Physical activity and all-cause mortality across levels of overall and abdominal adiposity in European men and women. Am J Clin Nutr.

[69] Beenackers MA, Kamphuis C, Giskes K, Brug J, Kunst AE, Burdorf A, Van Lenthe FJ (2012). Socioeconomic inequalities in occupational, leisure-time, and transport related physical activity among European adults: a systematic review. Int J Behav Nutr Phys Act.

[70] Dimova A, Rohova M, Koeva S, Atanasova E, Koeva-Dimitrova L, Kostadinova T, Spranger A, Organization WH (2018). Bulgaria: Health system review.

[71] Maresso A, Sagan A, Hernandez-Quevedo C, Williams G, Richardson E (2018). Organization and financing of public health services in Europe: country reports.

[72] Bulc M, Švab I, Yaphe J (2001). The countrywide integrated non-communicable disease intervention programme (CINDI) and the effects of healthcare system reform in Slovenia. Eur J Gen Pract.

[73] Wu F, Narimatsu H, Li X, Nakamura S, Sho R, Zhao G, Nakata Y, Xu W (2017). Non-communicable diseases control in China and Japan. Glob Health.

[74] Nomura S, Sakamoto H, Ghaznavi C, Inoue M (2022). Toward a third term of Health Japan 21–implications from the rise in non-communicable disease burden and highly preventable risk factors. Lancet Reg Health-West Pac.

[75] Townsend N, Kazakiewicz D, Lucy Wright F, Timmis A, Huculeci R, Torbica A, Gale CP, Achenbach S, Weidinger F, Vardas P (2022). Epidemiology of cardiovascular disease in Europe. Nat Rev Cardiol.

[76] Mahrouseh N, Lovas S, Njuguna DW, Nellamkuzhi NJ, Soares Andrade CA, Sackey WE, Irawan AS, Varga O (2022). How the European Union legislations are tackling the burden of diabetes mellitus: a legal surveillance study. Front Public Health.

[77] Lin X, Xu Y, Pan X, Xu J, Ding Y, Sun X, Song X, Ren Y, Shan P-F (2020). Global, regional, and national burden and trend of diabetes in 195 countries and territories: an analysis from 1990 to 2025. Sci Rep.

[78] Liu J, Ren Z-H, Qiang H, Wu J, Shen M, Zhang L, Lyu J (2020). Trends in the incidence of diabetes mellitus: results from the Global Burden of Disease Study 2017 and implications for diabetes mellitus prevention. BMC Public Health.

[79] Lugo A, La Vecchia C, Boccia S, Murisic B, Gallus S (2013). Patterns of smoking prevalence among the elderly in Europe. Int J Environ Res Public Health.

[80] Feliu A, Filippidis FT, Joossens L, Fong GT, Vardavas CI, Baena A, Castellano Y, Martínez C, Fernández E (2019). Impact of tobacco control policies on smoking prevalence and quit ratios in 27 European Union countries from 2006 to 2014. Tob Control.

[81] Gneiting U, Schmitz HP (2016). Comparing global alcohol and tobacco control efforts: network formation and evolution in international health governance. Health Policy Plan.

[82] Wood S, Bellis M (2017). Socio-economic inequalities in alcohol consumption and harm: Evidence for effective interventions and policy across EU countries.

[83] Siegfried N, Parry C (2019). Do alcohol control policies work? An umbrella review and quality assessment of systematic reviews of alcohol control interventions (2006–2017). PLoS One.

[84] Holmes J, Meng Y, Meier PS, Brennan A, Angus C, Campbell-Burton A, Guo Y, Hill-McManus D, Purshouse RC (2014). Effects of minimum unit pricing for alcohol on different income and socioeconomic groups: a modelling study. Lancet.

[85] Meier PS, Holmes J, Angus C, Ally AK, Meng Y, Brennan A (2016). Estimated effects of different alcohol taxation and price policies on health inequalities: a mathematical modelling study. PLoS Med.

[86] Hermansson U, Helander A, Brandt L, Huss A, Rönnberg S (2010). Screening and brief intervention for risky alcohol consumption in the workplace: results of a 1-year randomized controlled study. Alcohol Alcohol.

[87] Bono F, Matranga D (2019). Socioeconomic inequality in non-communicable diseases in Europe between 2004 and 2015: evidence from the SHARE survey. Eur J Public Health.

[88] Gańczak M, Miazgowski T, Kożybska M, Kotwas A, Korzeń M, Rudnicki B, Nogal T, Andrei CL, Ausloos M, Banach M (2020). Changes in disease burden in Poland between 1990–2017 in comparison with other Central European countries: a systematic analysis for the Global Burden of Disease Study 2017. PLoS ONE.

[89] Lorber J, Moore LJ. Gender and the social construction of illness. 2nd ed. Plymouth: Altamira Press; 2002.

[90] Courtenay WH (2000). Constructions of masculinity and their influence on men's well-being: a theory of gender and health. Soc Sci Med.

[91] Kocarnik JM, Compton K, Dean FE, Fu W, Gaw BL, Harvey JD, Henrikson HJ, Lu D, Pennini A, Xu R (2022). Cancer Incidence, mortality, years of life lost, years lived with disability, and disability-adjusted life years for 29 cancer groups from 2010 to 2019: a systematic analysis for the global burden of disease study 2019. JAMA Oncol.

