# Supplementary material for: Inequalities in the burden of non-communicable diseases across European countries: a systematic analysis of the Global Burden of Disease 2019 study
Source: Int J Equity Health. 2023 Jul 28;22:140. doi: 10.1186/s12939-023-01958-8 (PMC10375608; doi:10.1186/s12939-023-01958-8)
Supplement: Supplementary file 1 — Additional file 1: Figure: Age-standardized NCDs YLLs and YLDs rate for EEA Member States by sex, 1990–2019. Legend: UI 95% for the overall highest (Bulgaria) and lowest (Iceland) DALYs rate are shown in grey and blue shaded band, respectively. [file 12939_2023_1958_MOESM1_ESM.pptx]

## Slide 1
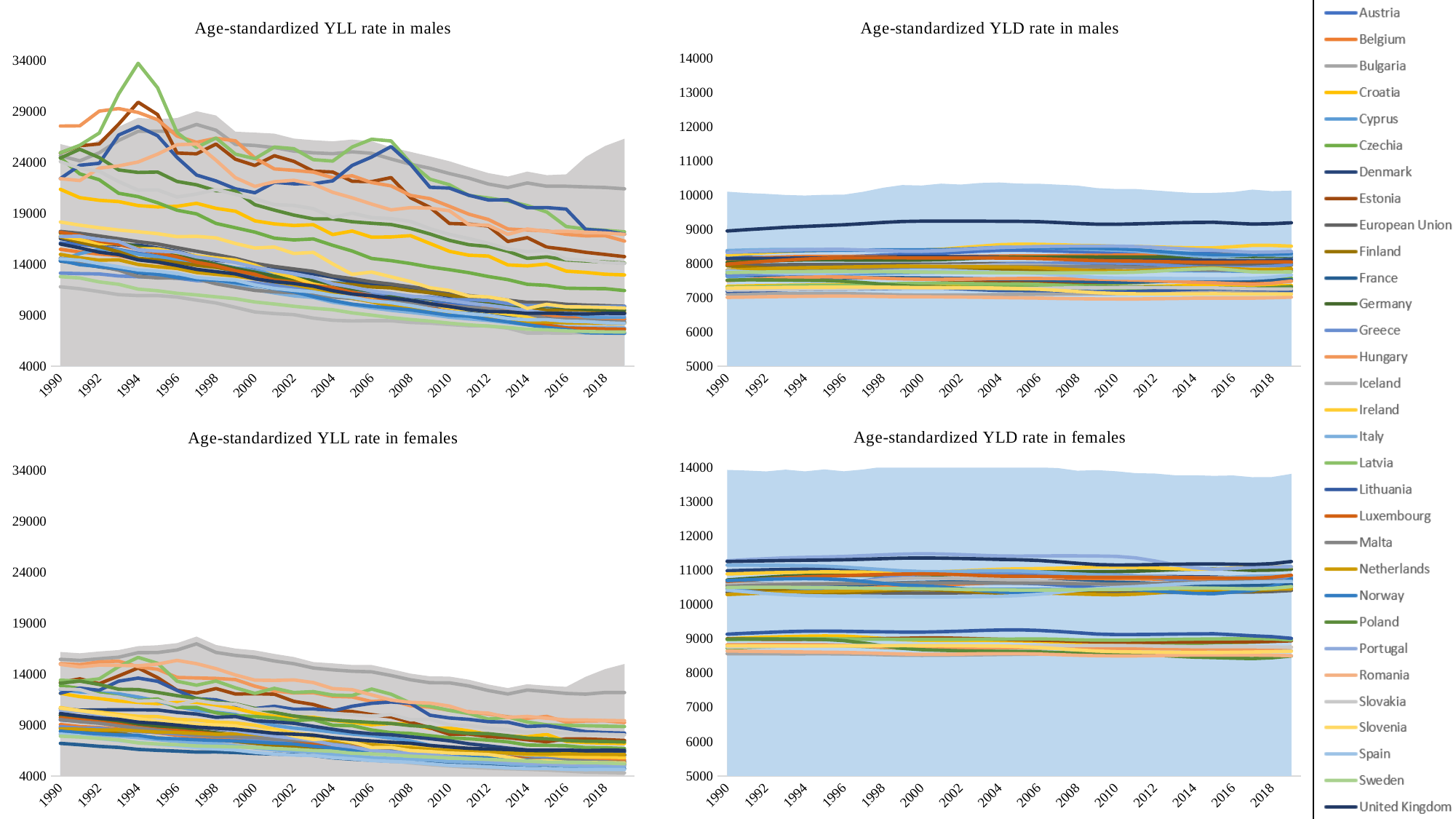

### Chart: Age-standardized YLL rate in males
| Category | z Bulg up | z Bulg low | z Ice up | z Ice low | Austria | Belgium | Bulgaria | Croatia | Cyprus | Czechia | Denmark | Estonia | European Union | Finland | France | Germany | Greece | Hungary | Iceland | Ireland | Italy | Latvia | Lithuania | Luxembourg | Malta | Netherlands | Norway | Poland | Portugal | Romania | Slovakia | Slovenia | Spain | Sweden | United Kingdom |
|---|---|---|---|---|---|---|---|---|---|---|---|---|---|---|---|---|---|---|---|---|---|---|---|---|---|---|---|---|---|---|---|---|---|---|---|
| 1990 | 25797.0511695302 | 23448.0885530969 | 12341.0977504047 | 11287.8629624237 | 16090.5562128029 | 15469.6555637594 | 24646.4537947771 | 21362.232746273 | 14358.7661607303 | 24438.8967769679 | 16521.3250633126 | 24929.3375652769 | 17225.5837795125 | 16669.7764268815 | 14317.9869767746 | 17060.901980791 | 13134.5517896968 | 27566.381704887 | 11793.7970672134 | 16775.1561005111 | 14533.5923568934 | 24941.035098107 | 22414.3975034425 | 17134.8992475606 | 14612.3289951431 | 14971.1983739904 | 14389.9191289529 | 24432.2869537978 | 16732.2929778379 | 22402.0276143567 | 24075.3461534443 | 18149.0999500569 | 14538.3591396178 | 12786.1380458787 | 15989.1825759258 |
| 1991 | 25333.8547915172 | 23004.170560611 | 12109.2770910768 | 11108.9049936034 | 15969.1908425325 | 15208.4844565302 | 24152.8520229129 | 20539.6281017785 | 15049.6218441421 | 22878.1043121755 | 16192.5805153578 | 25636.0077095728 | 17041.4671322785 | 16169.9484366794 | 14063.168435182 | 16812.5480933224 | 13088.2144439733 | 27588.8315926801 | 11600.9514569965 | 16425.3554763763 | 14395.2201427404 | 25679.3363746573 | 23721.5557675864 | 16833.9143052379 | 14167.279642658 | 14640.8647378107 | 13958.9630619157 | 25288.2569147098 | 16754.9869380035 | 22216.7730893302 | 23418.0068084427 | 17840.905882065 | 14404.2858336863 | 12655.7769454433 | 15614.954688706 |
| 1992 | 26187.0228927187 | 23753.6905772301 | 11820.0972977601 | 10831.9915000646 | 15733.8212427074 | 15005.4795973425 | 24944.1141788614 | 20281.6115549015 | 15462.8031838681 | 22297.0955586611 | 16032.7109089624 | 25812.0606085928 | 16776.8674306422 | 15738.9816421248 | 13747.3104883886 | 16364.7060592951 | 13053.9212593644 | 29031.939808424 | 11322.1341771771 | 16030.7315868905 | 14036.0540798688 | 26884.7830715352 | 23913.2696548207 | 16269.1467772189 | 13799.5343203202 | 14406.0535436139 | 13787.5143937894 | 24473.918921178 | 16423.2879991387 | 23440.8400406871 | 23130.8426981632 | 17581.6358932808 | 14066.9810098127 | 12272.0106498948 | 15188.8823526448 |
| 1993 | 27491.5077784638 | 24858.0376762425 | 11504.4308715647 | 10545.5756536093 | 15450.2662435228 | 14852.864615072 | 26159.4339069269 | 20156.5874839365 | 15401.5312420797 | 20971.2061467405 | 15893.6748052438 | 27754.855174791 | 16508.1330210414 | 15226.409655292 | 13496.3127102242 | 16110.4857861743 | 12856.5531982263 | 29281.7743606864 | 11024.1430709179 | 16007.7547428035 | 13671.6051006661 | 30719.3677011495 | 26687.110077492 | 15959.5040836931 | 13425.0327274295 | 14435.0783016219 | 13574.782645478 | 23261.0919400519 | 16222.9807422103 | 23654.7762993219 | 22118.9336629549 | 17359.4380066809 | 13756.0015685876 | 12034.889912664 | 14954.5764697995 |
| 1994 | 28370.4690844808 | 25781.5207879469 | 11420.194254267 | 10465.159948625 | 15078.5139384715 | 14460.1175140003 | 27053.5024529099 | 19759.3907430506 | 14986.3300888832 | 20639.3473310053 | 15884.7073798294 | 29906.4060276845 | 16218.9340545745 | 14571.6038587757 | 13164.2563913293 | 15740.462042061 | 12776.433046563 | 28898.472919868 | 10925.7694259573 | 15533.0056698283 | 13425.8671075103 | 33726.0806423299 | 27526.1501391261 | 15493.9553321726 | 12800.1220441673 | 13979.0925235773 | 13220.3264639591 | 23013.9115205619 | 15430.1236197397 | 24039.9815450776 | 21277.1252997001 | 17195.0800411215 | 13455.4068319971 | 11563.6612532927 | 14431.05719287 |
| 1995 | 28235.935060354 | 25805.1297620964 | 11415.8426383048 | 10440.2982461089 | 14806.4486195168 | 14188.0291614399 | 27066.4471337881 | 19628.1317620815 | 14741.7538449038 | 20038.7875372399 | 15595.203635352 | 28692.057508079 | 15992.9030683713 | 14427.7375057924 | 12953.4637270735 | 15358.5055927951 | 12750.0060521167 | 28190.7199930991 | 10932.7517299942 | 15512.0500389873 | 13101.7967308561 | 31319.4747389213 | 26618.0041818436 | 15128.2479112501 | 12666.433657901 | 13760.0957935701 | 13016.9703077732 | 23052.44613756 | 15288.2028341301 | 24813.5804966789 | 21304.877454134 | 17005.9462630907 | 13323.9785082141 | 11402.1204253742 | 14219.7967333284 |
| 1996 | 28355.0971325102 | 25830.7419695313 | 11264.5405133204 | 10284.0812266899 | 14509.9812035414 | 13808.8388596616 | 27055.1293644171 | 19689.2724002414 | 14479.7989333193 | 19305.2451003626 | 15263.4546702287 | 24919.6137383117 | 15629.5206893354 | 14169.2001610223 | 12770.85941446 | 14980.7658550752 | 12632.3099826573 | 26581.7466895475 | 10785.2992601577 | 15155.4966837907 | 12755.7603358166 | 26924.8171360217 | 24483.2664952228 | 14734.2545618221 | 12647.9448729762 | 13592.2633178445 | 12711.319431317 | 22128.7416182896 | 15204.9509541577 | 25734.2951248566 | 20609.1133888556 | 16709.0883285382 | 13121.8155251514 | 11156.3316854847 | 13884.2402576878 |
| 1997 | 29022.6301732285 | 26415.4619085531 | 10958.978950029 | 10011.9381397989 | 14124.9324324392 | 13480.0719169582 | 27720.3351822274 | 19987.7989347766 | 14279.4759085349 | 18945.7149226843 | 14793.2197281158 | 24842.9602602211 | 15281.461858879 | 13906.2640891885 | 12475.5562968515 | 14491.021798271 | 12399.7439480608 | 25971.2694362971 | 10483.6376756563 | 14823.5886359673 | 12490.6060029253 | 25469.8705953788 | 22760.5085267658 | 14198.7536381317 | 12583.4805218194 | 13192.3043083111 | 12520.2565176485 | 21802.2312148197 | 14721.7616167719 | 25801.4835616851 | 20897.7638906467 | 16745.6935395095 | 12706.564722993 | 10996.5576428868 | 13503.1616196286 |
| 1998 | 28602.2775471265 | 25763.4405161213 | 10657.5519370292 | 9737.17623066433 | 13762.2350203705 | 13156.9316426162 | 27156.0909188649 | 19489.3008301576 | 14038.0715076517 | 18008.9417223961 | 14393.827875754 | 25798.7671873151 | 14945.9637645016 | 13696.1875754304 | 12427.6798241539 | 13990.6502145628 | 12321.4238165381 | 26367.9901589447 | 10206.0212060057 | 14627.6278664719 | 12301.5000904219 | 26392.021458964 | 22179.3813047126 | 13862.8805820352 | 12081.8341541007 | 13016.5549859886 | 12447.2104918619 | 21222.8086860434 | 14478.6166470488 | 24213.1279347463 | 21340.3481547492 | 16588.6948993784 | 12559.7995022505 | 10806.2722150319 | 13274.9453855584 |
| 1999 | 27012.8320895499 | 24501.6835531933 | 10219.2516890126 | 9327.37349355075 | 13282.9153872244 | 12922.0565687919 | 25767.20188468 | 19205.3486812153 | 13666.0824299882 | 17581.5461447899 | 14239.6778954345 | 24311.5782279412 | 14586.097859227 | 13387.0137927556 | 12238.1674144942 | 13586.8607761189 | 12288.1414563182 | 26118.9373624238 | 9772.41956360577 | 14419.6796024726 | 11956.0495022196 | 24811.3792569747 | 21407.8919062442 | 13374.3540904416 | 11754.6550838254 | 12842.268992006 | 12202.9711399728 | 21180.9602188924 | 14162.5387130255 | 22500.3952257525 | 20858.5975804439 | 16037.4775023331 | 12444.3156153557 | 10618.7252261001 | 13051.3559642399 |
| 2000 | 26925.4240919347 | 24445.2054606259 | 9765.51029869337 | 8865.4443806656 | 12834.5817743237 | 12594.458748001 | 25660.3239655888 | 18261.7845595229 | 13446.1254461188 | 17158.8401433733 | 13691.6361164083 | 23699.615743951 | 14091.6304369916 | 12984.6340035949 | 11998.142909221 | 13232.0031021221 | 12170.7731094918 | 24427.7119849229 | 9319.00184778931 | 13919.3489290905 | 11517.1774666138 | 24366.2924686488 | 21024.6529717472 | 13029.0705086302 | 11437.1962211984 | 12572.3351475018 | 11924.229298638 | 19849.1007602738 | 13718.7068699808 | 21645.0912666268 | 20431.9933082198 | 15595.1804158606 | 11968.4338664859 | 10302.5139395302 | 12578.6289299763 |
| 2001 | 26825.0269466752 | 24251.2598796562 | 9628.36582896019 | 8717.32858494354 | 12356.3847086018 | 12247.3731936501 | 25452.548268262 | 17960.6063618061 | 13088.5387454494 | 16562.6608712364 | 13484.1124126185 | 24655.215649157 | 13787.3472201516 | 12626.3196082299 | 11837.2415726447 | 12840.0103644748 | 11961.5307195007 | 23360.5657343032 | 9164.94552897505 | 13206.7307494292 | 11190.1443462951 | 25508.0585867917 | 22044.3145077282 | 12411.4513716236 | 11296.720682435 | 12293.5689391082 | 11606.8750518518 | 19317.2242102987 | 13371.9370763842 | 22098.1886397303 | 19858.6089373053 | 15694.1379327884 | 11637.063098462 | 10106.9352412741 | 12299.7783644017 |
| 2002 | 26338.1517969932 | 23953.1724931808 | 9515.13378319029 | 8627.0811559852 | 12121.6446853156 | 12018.5206580577 | 25109.0650398307 | 17805.4578889972 | 12787.9940770164 | 16390.7916212959 | 13329.1096038011 | 24104.6979083401 | 13546.4955479783 | 12403.0491355733 | 11628.5302085938 | 12636.6937537776 | 11669.2280880673 | 23223.2336821361 | 9057.10243322875 | 12704.4448295734 | 10904.6233029234 | 25348.6923682189 | 21903.9539555066 | 12182.775107478 | 11209.8827866313 | 11990.1879315345 | 11308.5661694598 | 18828.0795135866 | 13084.906851663 | 22242.5309146597 | 19772.6174696177 | 15059.7703368201 | 11440.6164259222 | 9880.02066594898 | 12115.7740126992 |
| 2003 | 26180.0479821903 | 23794.6727688603 | 9152.1281907289 | 8298.22032257074 | 11904.6406526484 | 11691.8568365874 | 24939.0495645777 | 17880.5105897091 | 12327.0294646853 | 16486.038990423 | 13059.1173533344 | 23140.3423449111 | 13306.0994406915 | 12285.2080494647 | 11387.131540546 | 12428.5291224841 | 11524.3711177507 | 23050.5937734664 | 8720.52581566939 | 12063.2320216669 | 10740.318831934 | 24266.4617697926 | 21912.8727874061 | 11894.1295205645 | 11002.019010811 | 11627.2240741893 | 10830.7772198618 | 18457.8845234735 | 12719.5574822593 | 21876.3455822541 | 19454.3338151869 | 15166.6126671597 | 11290.7257159441 | 9691.14389258732 | 11853.5960938503 |
| 2004 | 26070.1673179049 | 23702.8804638004 | 8943.86255532384 | 8090.65645949091 | 11558.5268684861 | 11247.6524315452 | 24855.5472528536 | 16914.7487612516 | 12212.2249540068 | 15862.4501193127 | 12730.4287456196 | 23059.1209593566 | 12860.9321544959 | 12157.252914378 | 10845.9293951924 | 11885.4358264016 | 11421.4554765032 | 22502.9640468215 | 8507.03903889977 | 11703.5549831438 | 10278.1836819789 | 24129.046811343 | 22188.922841814 | 11709.9750725225 | 10770.2357785193 | 11131.4306635978 | 10435.4442931264 | 18442.2795133905 | 12086.7807384658 | 21068.8538155768 | 18593.0733887156 | 14039.7077505569 | 10955.0917223157 | 9544.23863321855 | 11414.2180571451 |
| 2005 | 26240.4894473522 | 23842.9207046928 | 8869.55974858054 | 8035.93433168997 | 11213.4556090342 | 10982.6762529683 | 25034.0960334693 | 17262.7379943701 | 12233.7746625395 | 15301.6245890507 | 12270.6715442694 | 22151.4524866158 | 12591.474139882 | 12000.2836623883 | 10657.6087158443 | 11522.8748569819 | 11249.5491393897 | 22691.4039760159 | 8441.07644398555 | 11173.5297423501 | 10014.0833707328 | 25507.4059747298 | 23704.7682905147 | 11249.4914076999 | 10674.5345532403 | 10685.7733547121 | 10090.5149992835 | 18171.8360151393 | 11757.4658993861 | 20526.6267638732 | 19018.1666582635 | 13015.9864379764 | 10748.8580186302 | 9236.45428082915 | 11084.8451390585 |
| 2006 | 26082.4197531066 | 23641.3304728228 | 8912.88688851629 | 8065.29470083877 | 10841.3419706542 | 10615.4444310162 | 24892.5361277335 | 16660.7310238127 | 12164.5567997605 | 14572.1609426604 | 12098.6239688028 | 22112.5889904401 | 12287.5755759166 | 11780.7475108389 | 10375.537415396 | 11200.773706887 | 11042.5321969931 | 22023.8514428514 | 8472.99121590632 | 10836.6577370057 | 9726.59024869293 | 26273.5393285441 | 24527.3228170855 | 10923.1416399777 | 10378.7443100743 | 10315.681610004 | 9883.63496095728 | 18027.5792758396 | 11392.4189282075 | 19909.7808050075 | 18581.7710054095 | 13239.8574681876 | 10405.1330085394 | 9012.38782169532 | 10906.01677518 |
| 2007 | 25482.9543366209 | 23162.9149051698 | 8907.3193748731 | 8036.14230854317 | 10633.9417163626 | 10425.6983192577 | 24335.6515098912 | 16682.9204320943 | 12076.3650883497 | 14354.8505480086 | 11851.5433336448 | 22508.0550131308 | 12056.4062784356 | 11687.430771719 | 10140.2709330337 | 10979.9072028058 | 11153.2048825072 | 21700.1635446919 | 8458.49395748069 | 10575.0551662408 | 9486.33435530876 | 26108.3309862332 | 25552.4037848755 | 10389.5562579945 | 10321.436679754 | 9907.52857885829 | 9703.43307541208 | 17897.8160348772 | 11148.8434164496 | 19336.6500023945 | 18481.9294638761 | 12770.763824354 | 10230.8709051558 | 8773.76976339862 | 10675.1855585163 |
| 2008 | 25032.9364667778 | 22706.8156552138 | 8733.17490302289 | 7879.64387803852 | 10399.9025838841 | 10289.0442084286 | 23825.7871797158 | 16808.685707856 | 11587.3208554813 | 14073.517530338 | 11497.3230055032 | 20529.188923872 | 11801.6948349218 | 11418.3929543926 | 9955.16316975721 | 10794.0263976097 | 10816.449965546 | 20762.6335285236 | 8289.70249685156 | 10345.90337416 | 9287.99691923021 | 23946.0381153245 | 23815.7591811785 | 9934.68323302827 | 10221.2193638855 | 9654.47530531944 | 9521.38592565157 | 17516.6498997596 | 10891.1690013174 | 19560.2843881146 | 18201.0745542901 | 12305.2069433646 | 9945.5317904413 | 8581.56142557046 | 10468.2723906947 |
| 2009 | 24583.4095076694 | 22331.9098991168 | 8656.40316245957 | 7828.9148982682 | 10374.4598019444 | 10106.1824049656 | 23425.5970635442 | 16040.4278723832 | 11416.003587201 | 13729.4659681969 | 11210.9569861842 | 19581.0694951986 | 11534.4333570728 | 11215.6695630022 | 9830.3224183548 | 10634.0537092753 | 10789.7654009145 | 20441.6161840255 | 8226.12813804333 | 10201.1250284696 | 9075.66747170602 | 22348.1327476361 | 21557.5173991686 | 9692.9967117153 | 9988.46623589757 | 9415.77571967816 | 9267.86969140897 | 16972.6113741292 | 10679.395568722 | 19515.5303744475 | 17488.6434482513 | 11698.3757189658 | 9619.39237788821 | 8425.36310467689 | 10141.7389670711 |
| 2010 | 24111.1137445594 | 21801.6909885641 | 8490.40935234024 | 7703.7202431038 | 10149.8478135599 | 9916.29458782104 | 22906.3282653037 | 15273.4252664419 | 11117.1910361437 | 13470.7825610213 | 10866.0479258822 | 18005.6379399 | 11237.8914135086 | 10961.014749836 | 9657.04799995003 | 10435.637889426 | 10540.4003593371 | 19686.9675705635 | 8089.62296854017 | 9557.21006407142 | 8808.75747725975 | 21807.8717563162 | 21482.1801802817 | 9370.25524919423 | 9885.00128244869 | 9208.21328399199 | 8994.33711800041 | 16349.0501623628 | 10434.465181626 | 19248.0011757235 | 16935.5835908093 | 11435.1365182018 | 9353.88157925416 | 8225.83526704732 | 9849.29036207771 |
| 2011 | 23511.5997198186 | 21406.2040375026 | 8387.07771374872 | 7597.09884416241 | 9884.6842693496 | 9720.99468978353 | 22459.711970258 | 14886.2019867208 | 10642.5905492148 | 13174.8425657115 | 10392.9452199958 | 17944.3751850168 | 10930.9857015771 | 10629.8944629386 | 9432.17687091972 | 10221.1884014516 | 10517.6781792891 | 18915.325528297 | 7967.44144027523 | 9547.9995676566 | 8645.25347430067 | 20752.9172201682 | 20775.4284822546 | 9115.11079142721 | 9774.15727611884 | 8938.27327474596 | 8908.49442514341 | 15920.2217914137 | 10181.6976966365 | 17894.9709061726 | 16522.643336311 | 10889.3391969093 | 9147.47098484716 | 8061.37625668182 | 9561.92778970957 |
| 2012 | 22951.9305722231 | 20798.4862017066 | 8343.49799140811 | 7576.01439912572 | 9698.21781688947 | 9664.88568438912 | 21875.0713034987 | 14805.7374909171 | 10343.3719491207 | 12801.0711172196 | 10054.5964874276 | 17752.1535605955 | 10742.466625448 | 10349.0075141503 | 9217.30908702841 | 10068.5925126703 | 10453.0549402767 | 18398.7584577612 | 7952.1818203992 | 9333.58828531762 | 8488.90782681155 | 20515.96855443 | 20312.027098068 | 8904.07498710942 | 9617.13070857912 | 8826.94544308855 | 8589.52318222236 | 15743.1147169514 | 9978.99814328004 | 17824.353039367 | 16049.0797571869 | 10796.1774000965 | 8985.83795092264 | 7934.25965534312 | 9372.32702846907 |
| 2013 | 22611.536762402 | 20532.832218278 | 8154.79337071284 | 7372.56612474069 | 9490.09731446771 | 9415.54525626079 | 21543.1372720687 | 13936.9306897748 | 10094.2624800105 | 12496.8173968371 | 9809.87256448507 | 16235.1920926615 | 10486.1389754335 | 10072.8483679249 | 8978.54053881743 | 10004.2314664213 | 10151.4777151816 | 17465.3081137597 | 7733.77656611231 | 9110.08486638448 | 8268.46444254204 | 20214.4810191817 | 20329.2322194282 | 8600.20206247538 | 9394.13870211887 | 8628.8506369446 | 8364.803452249 | 15280.5053993159 | 9736.25672309551 | 16933.4689050794 | 15496.3687029099 | 10489.7366574455 | 8685.20180591227 | 7801.38221288712 | 9328.24609322065 |
| 2014 | 23098.8284240208 | 20940.8462097142 | 7685.82870736761 | 6861.94360094921 | 9236.23092892288 | 9085.29635987525 | 21983.2903813645 | 13845.0242457416 | 9883.94410919029 | 12037.3370432544 | 9535.31186907365 | 16612.6974457105 | 10268.1450944619 | 9800.43708430223 | 8698.78561359029 | 9782.10405400474 | 9956.36476148571 | 17362.0025462225 | 7249.57153008392 | 8844.44388044587 | 8078.57720169984 | 19760.0297990429 | 19544.568816318 | 8340.39552681554 | 9154.18608849116 | 8349.02914514453 | 8067.51681971941 | 14581.3235581979 | 9463.1094544932 | 17463.5666869831 | 15252.3938248765 | 9673.96079892549 | 8559.27504024454 | 7630.79672678818 | 9187.87285988544 |
| 2015 | 22749.7251735991 | 20576.7955274416 | 7722.61646396203 | 6848.09458132268 | 9188.58118204972 | 8935.21451565067 | 21662.6537907364 | 14029.6314062482 | 9751.18945450899 | 11931.3652620629 | 9284.15073435537 | 15685.7936799593 | 10258.9973517547 | 9491.10285377769 | 8715.17393437569 | 9873.93058591933 | 9949.66073298717 | 17276.2585797181 | 7262.91346718555 | 8616.98292899378 | 8097.017017964 | 19129.8697490438 | 19566.4111679719 | 8041.11678841192 | 8667.87668424564 | 8327.93636229849 | 7797.67224634757 | 14729.2850871649 | 9207.10533409882 | 17221.6432884567 | 15378.0775349228 | 10073.4454661289 | 8567.47871968089 | 7575.11092535818 | 9202.67176080451 |
| 2016 | 22831.8728870489 | 20526.9625955094 | 7752.30143018548 | 6793.81975520776 | 8947.2999804086 | 8739.48681935047 | 21661.680103002 | 13325.7516739468 | 9312.11115006705 | 11657.1023797673 | 9255.46896362647 | 15457.12082328 | 10075.9706078212 | 9462.84846199339 | 8439.09227900616 | 9737.94481967691 | 9852.47902502578 | 16943.8736599395 | 7251.38845900798 | 8423.9372012152 | 7841.56341496136 | 17706.6945110936 | 19414.2027865295 | 7750.55938168594 | 8609.42392893454 | 8257.02870504179 | 7596.67003177012 | 14426.5928846696 | 9215.37604349802 | 17241.984459479 | 14508.6961907408 | 9833.30033594579 | 8416.9822406236 | 7466.12474806876 | 9174.0921117493 |
| 2017 | 24533.0261138602 | 18940.983025614 | 7843.58913457372 | 6784.46079797948 | 8747.7124086592 | 8719.74006633475 | 21595.9782849503 | 13206.9726926542 | 8852.97988008387 | 11625.2826056561 | 9221.07248936854 | 15173.6762522506 | 9998.36107345121 | 9448.7561718919 | 8423.97756857028 | 9607.18565068902 | 9834.18943014887 | 16791.6987071603 | 7283.2795925719 | 8368.99969271223 | 7803.22960674957 | 17460.3854714086 | 17427.2080408797 | 7699.70177264135 | 8513.29328050448 | 8232.46755974086 | 7284.16792001164 | 14320.8291290155 | 9160.97668557578 | 17146.6702582464 | 14401.3005527147 | 9812.42139937631 | 8373.83830334297 | 7408.91918895424 | 9121.7818922325 |
| 2018 | 25613.6212317965 | 17832.9165309417 | 7875.07317501729 | 6700.77154824053 | 8603.19105794802 | 8660.41424142593 | 21539.2839244106 | 13023.3831106662 | 8816.25955184681 | 11609.6601634132 | 9205.46461287872 | 14964.4443096341 | 9944.19686369521 | 9356.51424537252 | 8319.73259510373 | 9547.96394348175 | 9846.07389507363 | 16805.6406043304 | 7236.85362250225 | 8301.27423559598 | 7781.33025227423 | 17335.4519780836 | 17290.7765043238 | 7656.76059451901 | 8426.5542500807 | 8206.69621126613 | 7259.83529600857 | 14231.6115315545 | 9084.34776949219 | 17074.3423502699 | 14179.4865819662 | 9764.56913390192 | 8263.56049253264 | 7371.37449807483 | 9188.63919423141 |
| 2019 | 26325.018322595 | 17320.7527612431 | 7913.22059125371 | 6588.70097155288 | 8551.73888170552 | 8661.62995380427 | 21409.0152679108 | 12956.0081453785 | 8816.7100098625 | 11422.6293052168 | 9188.52714468759 | 14747.995018857 | 9887.49647957275 | 9296.18958823099 | 8306.46610516404 | 9496.75257731612 | 9871.43574325644 | 16276.5428429238 | 7201.25743143217 | 8272.19469945271 | 7745.85406005479 | 17185.90602518 | 16946.6322934656 | 7629.77152579523 | 8337.03846612124 | 8195.42398522746 | 7260.02070698676 | 14119.8470960908 | 9058.48222679423 | 16938.821943974 | 14084.2615651681 | 9705.16484796373 | 8200.30563032697 | 7360.10066660131 | 9179.63927369123 |
### Chart: Age-standardized YLD rate in males
| Category | z Ice up | z Bulg up | z Ice low | z Bulg low | Austria | Belgium | Bulgaria | Croatia | Cyprus | Czechia | Denmark | Estonia | European Union | Finland | France | Germany | Greece | Hungary | Iceland | Ireland | Italy | Latvia | Lithuania | Luxembourg | Malta | Netherlands | Norway | Poland | Portugal | Romania | Slovakia | Slovenia | Spain | Sweden | United Kingdom |
|---|---|---|---|---|---|---|---|---|---|---|---|---|---|---|---|---|---|---|---|---|---|---|---|---|---|---|---|---|---|---|---|---|---|---|---|
| 1990 | 10101.4780403083 | 9113.78152397532 | 5714.77575233713 | 5297.84092032127 | 8190.61999533569 | 8118.97285240675 | 7100.46499005607 | 7332.14792909883 | 7621.17736477577 | 7282.22658282209 | 8146.23854069311 | 7199.88221780184 | 7944.86308554005 | 7935.11967890445 | 7693.78498150233 | 8035.09497806428 | 7658.76579032053 | 7499.94406008878 | 7795.50048333537 | 8246.71224610292 | 8379.36549019637 | 7303.82494803711 | 7184.45239946551 | 7981.888315053 | 7752.46645961249 | 7806.75536076605 | 8332.70878913627 | 7512.31184888677 | 8324.08812474195 | 7010.40542779212 | 7222.5993761833 | 7281.44806217872 | 7790.26898701575 | 7731.68892396221 | 8950.27762529591 |
| 1991 | 10060.4899954612 | 9123.02720940903 | 5728.78235484332 | 5296.96799163548 | 8181.3232107693 | 8148.12442975249 | 7108.46923404775 | 7343.97032932729 | 7633.91033529449 | 7289.49989938026 | 8146.71591394937 | 7211.78893703008 | 7952.49615764266 | 7897.9108554509 | 7664.47612969615 | 8056.46410375282 | 7671.87977425356 | 7529.5207436838 | 7781.24403230192 | 8282.29010130518 | 8395.34554159588 | 7315.31693322171 | 7199.91537484445 | 8026.01174345452 | 7756.3936274028 | 7828.35626591072 | 8342.94651022228 | 7522.18994210716 | 8350.30082106043 | 7017.75338109735 | 7228.48426750603 | 7290.69204863538 | 7756.92457811587 | 7745.29977490305 | 8988.58418552986 |
| 1992 | 10037.8355141989 | 9114.78446509347 | 5721.17477122637 | 5309.33425287796 | 8170.62392832452 | 8173.35484536098 | 7114.81177602384 | 7354.11835555332 | 7646.17507707101 | 7298.35557239657 | 8149.93658529565 | 7224.88840275987 | 7958.71151427958 | 7870.1420158885 | 7638.85471213272 | 8074.48854646778 | 7683.08497174327 | 7560.65877972899 | 7768.29517388556 | 8315.50607212889 | 8405.63070098802 | 7328.10823974996 | 7212.67695909373 | 8064.58667218764 | 7763.20820155502 | 7847.14878759198 | 8356.49128757813 | 7521.3837073137 | 8371.99449957511 | 7028.7927222963 | 7235.10299006922 | 7295.7825678897 | 7729.41396026721 | 7752.42266517919 | 9023.58387784675 |
| 1993 | 10004.934484914 | 9116.626737573 | 5728.00271316037 | 5313.00932096671 | 8167.68232877123 | 8193.91286244766 | 7117.90314862353 | 7363.49719828363 | 7660.03812239984 | 7305.12227573666 | 8154.77187265721 | 7246.97444234137 | 7964.72987572247 | 7847.64500734171 | 7618.39952778228 | 8092.05084786426 | 7690.5858867385 | 7582.9999778839 | 7759.09066849994 | 8347.51196313634 | 8410.56898906317 | 7346.56170719573 | 7233.69340959411 | 8099.80163988264 | 7769.08519619023 | 7868.52645855607 | 8366.56420934651 | 7514.48593007667 | 8391.07600176757 | 7034.49060508597 | 7241.49798719641 | 7301.0252896716 | 7711.94401827252 | 7760.04925960759 | 9057.73338197125 |
| 1994 | 9988.13098893742 | 9132.48401204425 | 5705.47636293956 | 5312.68389729573 | 8167.80302242748 | 8207.42998359483 | 7122.92263805321 | 7369.27398753733 | 7671.39281454502 | 7316.96260114177 | 8163.25482089268 | 7269.01638345385 | 7968.7554115308 | 7828.96154647216 | 7602.0069644975 | 8102.95788916637 | 7699.24546852776 | 7599.28213926402 | 7757.50258834967 | 8368.63697730768 | 8411.64048966119 | 7361.89141028394 | 7245.09833469422 | 8130.40628505453 | 7774.22558236746 | 7878.42827443447 | 8374.03664672208 | 7508.23620848988 | 8401.03816628294 | 7040.56386437752 | 7247.09838821 | 7305.18209781797 | 7704.9234105453 | 7762.89924993328 | 9083.35562004905 |
| 1995 | 10009.2713301062 | 9180.77720338432 | 5725.27960610056 | 5289.24088125896 | 8176.40869223553 | 8210.10893145483 | 7124.90772598012 | 7375.58039397711 | 7677.81363964059 | 7322.83017770914 | 8165.81339120948 | 7280.91980273099 | 7971.25525732342 | 7820.69343374704 | 7591.30550189598 | 8104.91023180201 | 7703.28852910238 | 7609.65617352109 | 7757.21883201505 | 8388.2776793269 | 8402.85061411374 | 7363.40385911742 | 7251.63492718937 | 8154.56116487032 | 7781.676162216 | 7885.55960611769 | 8382.18204374506 | 7502.74187419883 | 8412.95152188834 | 7045.3709677271 | 7255.63595596323 | 7307.49077316657 | 7712.77169903764 | 7768.78359579742 | 9107.2738612575 |
| 1996 | 10013.3368713512 | 9103.02490310485 | 5768.31543298752 | 5300.16631024357 | 8194.74615401683 | 8206.32577865514 | 7120.77256031404 | 7382.01054808485 | 7685.50436991382 | 7321.40078255475 | 8174.22174689807 | 7290.0993455723 | 7972.09758611022 | 7818.74247251486 | 7585.01808357602 | 8101.71429168424 | 7718.37797412205 | 7604.42642593338 | 7777.54172031029 | 8393.87770930827 | 8389.5100642758 | 7363.72890979488 | 7255.59870730548 | 8167.46399002893 | 7793.57857305861 | 7897.78952390626 | 8385.58984152669 | 7481.42912525847 | 8412.35904186599 | 7046.67135014916 | 7260.92977041022 | 7310.04114408618 | 7729.85406126417 | 7768.52230366701 | 9133.03396784025 |
| 1997 | 10099.0221913578 | 9129.60415091839 | 5800.38797106817 | 5298.41402367936 | 8218.39830693006 | 8204.95957685831 | 7110.94003442964 | 7384.96245794997 | 7699.74091028175 | 7313.41798176644 | 8182.20420687115 | 7318.84892672604 | 7973.20447767417 | 7827.79065627786 | 7581.99999497152 | 8099.73969753231 | 7751.31098056587 | 7586.09323978414 | 7822.38995535081 | 8392.66730335872 | 8379.1493535837 | 7379.59415484571 | 7261.38152526879 | 8171.17194586975 | 7819.192455064 | 7906.21211995994 | 8392.50704334645 | 7442.63234542626 | 8396.26091899006 | 7042.97617142797 | 7268.39267002422 | 7310.42650859365 | 7749.73196799518 | 7766.49356880928 | 9165.05198557033 |
| 1998 | 10214.3306216083 | 9080.51628481491 | 5847.39676677545 | 5282.1326891792 | 8245.90652029373 | 8200.78462787238 | 7093.9437174222 | 7381.14006395949 | 7716.29298652501 | 7300.69567062159 | 8191.09834479061 | 7355.08420889549 | 7975.43935977986 | 7839.84732320504 | 7584.20483814024 | 8097.43840613353 | 7790.40019527135 | 7567.66937038353 | 7877.09115045143 | 8388.45776430117 | 8371.58010912259 | 7402.26373197218 | 7270.6951098758 | 8171.25934022423 | 7847.50133476231 | 7918.38340189392 | 8400.55368949457 | 7395.34823026688 | 8377.96466909645 | 7034.8606697554 | 7277.69954847527 | 7307.85156433621 | 7773.46102054614 | 7762.5917197996 | 9200.20474125994 |
| 1999 | 10295.0338546671 | 9081.74353775954 | 5867.74462829214 | 5233.24788268424 | 8268.0507581321 | 8201.31073510183 | 7079.8011932573 | 7378.19308298308 | 7730.92676388712 | 7291.15423796632 | 8202.88547794776 | 7380.82012722583 | 7977.09949822233 | 7850.14341255244 | 7582.91802137263 | 8099.36112113056 | 7828.97464029954 | 7549.12107343162 | 7920.09673505006 | 8387.6548025675 | 8363.45839829519 | 7415.33744082429 | 7277.29881971754 | 8166.14331440633 | 7871.4909524029 | 7925.39826564213 | 8401.38176020482 | 7354.89157135915 | 8362.74097848277 | 7026.73626011772 | 7281.89519469205 | 7302.31709315975 | 7792.34050048847 | 7756.46196409714 | 9227.8897782788 |
| 2000 | 10281.1575401575 | 9104.54360093033 | 5871.49499111672 | 5217.27487878736 | 8282.40548197527 | 8201.23979002679 | 7074.58733757999 | 7372.38316771268 | 7741.0806747783 | 7287.42733551538 | 8200.4841970307 | 7404.67453925657 | 7976.26195316489 | 7853.05933163394 | 7576.91203623065 | 8102.44214319284 | 7847.42774274665 | 7538.51479222348 | 7935.47360678411 | 8393.49740674448 | 8356.71995563441 | 7422.52999144841 | 7282.65796644929 | 8164.32604335736 | 7886.32711754865 | 7920.08142689167 | 8401.32672689385 | 7326.09532013899 | 8362.34106512109 | 7024.78396733421 | 7285.21961831364 | 7296.84968606984 | 7799.58942857562 | 7749.24232093892 | 9238.0907304164 |
| 2001 | 10335.7034112993 | 9056.05824931083 | 5881.44706321283 | 5239.54559580987 | 8300.42445821925 | 8201.12753792009 | 7076.17470512881 | 7374.91887751889 | 7747.47625008052 | 7289.16128934073 | 8199.41321372389 | 7428.0242877793 | 7979.45424810665 | 7848.43009685466 | 7571.78524794782 | 8112.0209044918 | 7868.11058287155 | 7539.36850392629 | 7936.52305113251 | 8416.39966974021 | 8371.03105882252 | 7426.00004545665 | 7279.72258716967 | 8163.48836810197 | 7893.43781955013 | 7915.36580372118 | 8403.95518853445 | 7309.9532890786 | 8378.40245300759 | 7023.16753759283 | 7285.17897716142 | 7295.73924465899 | 7795.46249929053 | 7744.42553312563 | 9239.62510491451 |
| 2002 | 10308.3092651185 | 9057.78514030582 | 5869.69603155037 | 5263.39033073146 | 8330.55237658696 | 8207.72626147861 | 7079.58705777895 | 7380.0394804447 | 7750.10640215027 | 7305.85563945297 | 8198.93062753245 | 7442.24430422691 | 7986.72381215834 | 7840.69005031549 | 7562.57127169166 | 8133.99882842416 | 7899.84739708842 | 7545.74028573105 | 7941.4116011102 | 8464.0555206193 | 8405.4678601809 | 7420.32556037448 | 7259.50476781335 | 8163.83969746241 | 7901.17390257998 | 7910.0411300945 | 8421.32817293976 | 7292.4841353789 | 8407.57166213615 | 7018.92148337657 | 7281.94993267804 | 7291.78686427276 | 7780.06531322242 | 7736.22852923098 | 9239.73314780361 |
| 2003 | 10355.2312773899 | 9074.07063962456 | 5864.0038684768 | 5280.21796976109 | 8361.91055229632 | 8213.43558815064 | 7083.20908567486 | 7390.24501339769 | 7751.9188243458 | 7326.90506800564 | 8194.26421915257 | 7453.08706265143 | 7995.57421380815 | 7834.14895875693 | 7551.30146757885 | 8159.60939425039 | 7943.92441378492 | 7552.91461206485 | 7945.08173712501 | 8505.84936637191 | 8448.97160287573 | 7408.49185345818 | 7232.70126586975 | 8162.64156479402 | 7906.48553058208 | 7902.41082821467 | 8440.9335368896 | 7275.12974533281 | 8439.12285314774 | 7013.02536481386 | 7274.48343744133 | 7291.11368331992 | 7761.59645301718 | 7730.57985244661 | 9238.41028169518 |
| 2004 | 10369.5443828824 | 9137.19514744416 | 5845.36680046892 | 5266.97193784543 | 8383.58091767466 | 8217.24582024118 | 7091.45217697316 | 7390.61640571175 | 7756.51935393689 | 7341.97210998667 | 8189.32511097272 | 7464.73115408561 | 7999.9780192547 | 7825.9798172713 | 7535.32859654743 | 8178.99416935175 | 7980.62496558424 | 7556.58543559272 | 7948.59734033265 | 8549.42603673559 | 8475.97243794605 | 7400.76303942407 | 7211.54270692936 | 8159.19324316541 | 7911.32263381573 | 7901.27940519749 | 8457.65187993221 | 7262.39972701805 | 8463.91320323975 | 7004.15575494093 | 7267.6550700297 | 7281.26372837935 | 7742.2745611448 | 7729.24415839006 | 9234.12588684982 |
| 2005 | 10336.2917613597 | 9121.35675056729 | 5865.01205019207 | 5262.82055003075 | 8388.59319504852 | 8224.15309548708 | 7096.663871987 | 7393.11657413931 | 7761.12279787621 | 7351.33838907989 | 8181.96935767607 | 7470.93379215215 | 8001.25652680516 | 7821.56098559152 | 7530.28877382961 | 8186.28285800595 | 7999.90553473224 | 7561.26970327966 | 7951.42998471482 | 8565.22653821233 | 8481.08691892259 | 7401.42450356119 | 7208.57984737418 | 8153.43260175046 | 7913.03431486139 | 7892.00698396664 | 8463.33024926019 | 7254.1518175407 | 8482.56491493456 | 6997.52684516864 | 7270.58152646098 | 7266.02061674525 | 7730.94301410368 | 7725.87610981709 | 9231.93877620542 |
| 2006 | 10333.7835645913 | 9106.82776537596 | 5857.78249430121 | 5279.14310569646 | 8376.14479684165 | 8224.0733107792 | 7102.85571576643 | 7382.04476406837 | 7767.49066386998 | 7358.60778897433 | 8183.00799624665 | 7473.92643399395 | 7995.89541063355 | 7817.78209965685 | 7518.42827143832 | 8188.31510454718 | 8002.90535123939 | 7561.40729053493 | 7946.93459121365 | 8560.42532268412 | 8465.93680435069 | 7392.06368601846 | 7207.15625645685 | 8143.98340907918 | 7913.56700601521 | 7875.85758599591 | 8462.77047383998 | 7258.20702062151 | 8495.96585631793 | 6990.06989598004 | 7274.2908228099 | 7248.36130491185 | 7711.69278624008 | 7726.14825668885 | 9223.02580134983 |
| 2007 | 10306.2359512636 | 9085.4385665126 | 5858.30446887471 | 5285.63164175622 | 8362.34425920858 | 8228.61583826582 | 7108.06530438125 | 7365.78387585974 | 7780.16943886782 | 7373.20236485242 | 8184.99494378072 | 7473.46914834619 | 7985.19353781467 | 7813.99144342514 | 7501.26162560504 | 8188.70109657264 | 8010.75370066759 | 7559.72603006013 | 7933.13717970118 | 8547.89672350656 | 8447.31728558374 | 7365.6044555758 | 7202.7386356712 | 8124.23880514215 | 7912.28712786974 | 7844.17379687739 | 8457.75102714306 | 7272.00109440317 | 8503.77196631529 | 6978.2085732718 | 7281.59370743397 | 7215.66059428632 | 7673.64541453716 | 7727.77365000118 | 9197.52707144447 |
| 2008 | 10279.2376655529 | 9111.85313281914 | 5838.78688134834 | 5284.17674253692 | 8345.0126073097 | 8234.7376794721 | 7112.18268707664 | 7347.35613492944 | 7792.14424699514 | 7389.06906131748 | 8181.08268900072 | 7460.03260819836 | 7973.32950498172 | 7810.43940154418 | 7481.38500893775 | 8189.96912745042 | 8008.92532083264 | 7551.6847953003 | 7914.62529955803 | 8530.25400810547 | 8428.0230468409 | 7327.67718569369 | 7187.81166103003 | 8102.35984794314 | 7909.29932974096 | 7811.67940363253 | 8453.0387431992 | 7289.57853723117 | 8508.19627562806 | 6971.53274349183 | 7292.01182129054 | 7179.4207299188 | 7630.91101383703 | 7727.96265501111 | 9169.84357479372 |
| 2009 | 10205.1831335331 | 9107.99041945929 | 5858.45446077391 | 5291.03426117677 | 8332.98459052502 | 8239.48432617188 | 7114.59283920086 | 7327.8739539148 | 7802.62599577741 | 7405.90390401549 | 8181.30715682602 | 7455.11434946913 | 7963.34012118639 | 7809.30115561752 | 7463.98839534492 | 8189.29325876648 | 8012.46471556381 | 7544.12102469931 | 7900.08826166241 | 8523.02978311518 | 8408.21791389095 | 7294.15517771601 | 7173.18365771952 | 8086.19679064444 | 7905.43693836421 | 7782.86021373847 | 8443.29626102475 | 7306.10948099403 | 8509.25176726774 | 6964.71747692345 | 7297.39557846191 | 7145.61709119038 | 7592.01787626591 | 7730.38818181786 | 9148.50327719598 |
| 2010 | 10177.8976060188 | 9190.54982536786 | 5849.44909221954 | 5286.63517544103 | 8321.90496114035 | 8242.78990968134 | 7118.61703609223 | 7316.37891656777 | 7806.90713765036 | 7424.13943725046 | 8173.45238128105 | 7450.15944711473 | 7960.30096655504 | 7815.92140579501 | 7455.03068214488 | 8191.47707353041 | 8012.01768111898 | 7531.75340956518 | 7887.9726763337 | 8501.27007439767 | 8388.89893321569 | 7276.72978417862 | 7173.2157875604 | 8076.7383146509 | 7901.41814766387 | 7773.55463349323 | 8427.4211043233 | 7317.09776142541 | 8508.43382504018 | 6963.46356036773 | 7300.7741870588 | 7123.15609696959 | 7574.08490543777 | 7732.88513403264 | 9146.1676742707 |
| 2011 | 10178.3575954348 | 9149.13599612513 | 5800.99156231307 | 5267.21629589175 | 8314.29092540206 | 8247.36845870689 | 7124.30566129434 | 7320.27537699349 | 7801.44764821124 | 7446.3288156316 | 8155.41160768854 | 7462.26461511182 | 7961.81695872829 | 7827.8241061714 | 7459.8367106757 | 8191.30821496295 | 8003.2406948164 | 7515.39895533472 | 7875.06972062266 | 8496.60042336242 | 8361.65072593005 | 7268.37111402564 | 7182.13213592859 | 8070.71295454466 | 7888.0626464035 | 7784.21209706053 | 8405.78990525431 | 7317.38264183831 | 8495.95429146877 | 6962.76889597844 | 7301.74460465655 | 7113.91580279122 | 7568.14683774383 | 7746.16002707291 | 9158.54453201136 |
| 2012 | 10137.3793429491 | 9111.02447853345 | 5800.46048013143 | 5275.36332015747 | 8307.10816389851 | 8252.2335881185 | 7131.47959851781 | 7336.94598839058 | 7796.94715876114 | 7471.30098232721 | 8136.65057774075 | 7477.69111532626 | 7964.25883583862 | 7844.89892530097 | 7473.9858766487 | 8193.48473390153 | 7972.48329197652 | 7494.50480503868 | 7858.83299510653 | 8483.66399223146 | 8317.806565746 | 7268.56935487492 | 7202.78080759731 | 8059.70268339699 | 7865.56354299474 | 7812.54115216616 | 8362.44403366823 | 7310.6146731463 | 8471.15389986096 | 6971.44867939501 | 7298.60300378548 | 7113.01285747681 | 7566.05094384547 | 7774.5506831469 | 9173.48785995498 |
| 2013 | 10095.9498404136 | 9191.72967162797 | 5807.51833351726 | 5309.71993854361 | 8301.19455058165 | 8254.66767843915 | 7140.8424226432 | 7347.88085937812 | 7792.12590696205 | 7496.73329632503 | 8119.1740446494 | 7484.98912606346 | 7966.61736500491 | 7862.65409773455 | 7491.27722839171 | 8199.81510322406 | 7930.75658284329 | 7470.62126899011 | 7839.41006538594 | 8469.04130779346 | 8269.09723084215 | 7273.04408527812 | 7229.07447516346 | 8048.36641319748 | 7839.14322181014 | 7841.37699503792 | 8321.5610982874 | 7299.2077559283 | 8442.47332127078 | 6977.45215456363 | 7294.39098611085 | 7114.07756818906 | 7560.82828062178 | 7807.45258728659 | 9190.12873289215 |
| 2014 | 10060.3958462211 | 9149.40739711159 | 5787.08694577219 | 5282.01644145233 | 8296.74786223879 | 8256.30501076052 | 7151.91042511651 | 7363.12736892987 | 7788.35806539106 | 7517.55072863005 | 8103.42936037723 | 7503.19617359711 | 7968.58499713683 | 7878.00514409049 | 7506.94206582672 | 8203.16891955894 | 7899.01844587052 | 7450.72212028957 | 7821.66257161688 | 8460.2956331961 | 8227.42513983632 | 7276.90578885691 | 7248.1995285726 | 8038.72799656148 | 7817.37698658138 | 7862.23520212086 | 8282.63742726361 | 7288.43543787431 | 8413.60567810907 | 6986.77741568323 | 7289.47007185254 | 7109.32651490608 | 7558.33171796312 | 7835.43185254877 | 9199.99754558167 |
| 2015 | 10064.8142025867 | 9205.57752049215 | 5761.61887009157 | 5290.69754234103 | 8296.52820137607 | 8260.00585845886 | 7157.13508798757 | 7370.88212937631 | 7786.93335001556 | 7533.22873185541 | 8091.87920284671 | 7504.32287894277 | 7973.77672507428 | 7886.14921227269 | 7517.63264659081 | 8214.82170485768 | 7885.23437330437 | 7436.9099239323 | 7822.05854535642 | 8460.61311942605 | 8211.43677163282 | 7277.25235827175 | 7259.09206392125 | 8036.64610917795 | 7805.94413963939 | 7871.56428271013 | 8259.35703623166 | 7288.2611114684 | 8389.60569679225 | 6988.90357378798 | 7288.95183184345 | 7115.16918040473 | 7553.80605770788 | 7853.73042431259 | 9204.80369289196 |
| 2016 | 10089.3343729641 | 9186.17600775854 | 5768.9742901852 | 5305.58489811428 | 8301.47229403271 | 8268.32779595134 | 7156.24273654186 | 7351.49301136187 | 7776.9365049706 | 7546.32115472108 | 8085.46308522211 | 7495.45417780118 | 7970.36368587882 | 7877.09700909682 | 7513.26261531494 | 8208.75772699043 | 7885.51903691925 | 7411.75014939287 | 7835.62946758927 | 8491.43045588776 | 8226.48304479829 | 7268.58437159066 | 7252.5834821524 | 8039.44796065442 | 7811.39321092442 | 7847.69377542579 | 8259.64150458062 | 7278.94733670853 | 8354.54802904377 | 6988.41117496949 | 7270.76169224576 | 7107.99199859772 | 7553.73126079722 | 7807.35610696571 | 9179.29284748791 |
| 2017 | 10161.3559975019 | 9154.14154993764 | 5771.37548352576 | 5330.04751611497 | 8305.6196716498 | 8276.65448090934 | 7158.28764011001 | 7335.96038637192 | 7767.47998935943 | 7559.07733284035 | 8081.38539256886 | 7486.83326481993 | 7969.86420504397 | 7872.98386555734 | 7512.79555481714 | 8202.35940204964 | 7890.84438475707 | 7395.40217739234 | 7845.58885357105 | 8530.05194036156 | 8251.17821670497 | 7267.11911671014 | 7234.74315638618 | 8048.57575915651 | 7820.49006431298 | 7821.39504225933 | 8261.45178616164 | 7271.78401485492 | 8328.87120779644 | 6988.54969684896 | 7258.41667747553 | 7105.62820738936 | 7565.37078038829 | 7753.513784369 | 9152.77576050833 |
| 2018 | 10116.0196296314 | 9205.93514204722 | 5762.86159407438 | 5334.73260855339 | 8261.45008134286 | 8277.45673178801 | 7165.89604818287 | 7342.22081588812 | 7772.6139434813 | 7569.36038494436 | 8090.75578964914 | 7484.82335103599 | 7980.37500295283 | 7902.67248146223 | 7516.43016522154 | 8207.17253892477 | 7917.14283559039 | 7424.03637343089 | 7841.54978355103 | 8528.93214228794 | 8248.49687764063 | 7282.41387696909 | 7228.49279185037 | 8050.11635659207 | 7815.31713907719 | 7828.05888102688 | 8270.19799134126 | 7289.89258313694 | 8334.9308318389 | 6998.78627352567 | 7255.1500596854 | 7106.84803940239 | 7598.88325694416 | 7749.1168820579 | 9162.42612646722 |
| 2019 | 10129.7232777449 | 9218.55114787507 | 5759.57329451483 | 5337.24780094262 | 8164.26615158729 | 8268.91596689851 | 7179.89911858845 | 7358.84734110035 | 7782.19305703385 | 7577.02481086081 | 8112.00543399235 | 7486.1599450881 | 7997.65949965855 | 7947.18556762505 | 7524.97536287872 | 8216.63900800816 | 7963.34681619569 | 7486.37617364541 | 7832.13389731067 | 8506.80000657302 | 8231.01496410248 | 7310.9942499839 | 7213.53399014698 | 8045.72387482468 | 7798.75413813922 | 7848.82345929646 | 8290.90614410227 | 7325.45509978837 | 8353.27212350722 | 7016.40675392742 | 7256.45588569561 | 7106.67874230988 | 7663.39689829581 | 7754.82754852835 | 9190.80051263185 |
### Chart: Age-standardized YLD rate in females
| Category | z Ice up | z Bulg up | z Ice low | z Bulg low | Austria | Belgium | Bulgaria | Croatia | Cyprus | Czechia | Denmark | Estonia | European Union | Finland | France | Germany | Greece | Hungary | Iceland | Ireland | Italy | Latvia | Lithuania | Luxembourg | Malta | Netherlands | Norway | Poland | Portugal | Romania | Slovakia | Slovenia | Spain | Sweden | United Kingdom |
|---|---|---|---|---|---|---|---|---|---|---|---|---|---|---|---|---|---|---|---|---|---|---|---|---|---|---|---|---|---|---|---|---|---|---|---|
| 1990 | 13931.8089303926 | 11129.3449049257 | 7801.29949444662 | 6311.54874246113 | 10639.176928491 | 10474.9904237093 | 8564.36292503311 | 9002.41810261376 | 10463.4912027585 | 8722.74175651934 | 10987.9410573467 | 8998.17502609185 | 10356.1911007979 | 10566.5756780989 | 10679.6116384503 | 10718.518327942 | 10606.1107382724 | 8827.21324352888 | 10610.3163133581 | 10894.7259691763 | 11151.0884101452 | 8977.61051864265 | 9134.84081633 | 10676.765430798 | 10535.9913732427 | 10293.8482435156 | 10700.0877767315 | 8991.39431457444 | 11279.1448159199 | 8645.1952910378 | 8760.28410367461 | 8795.95300039084 | 10421.6030903387 | 10498.9055095032 | 11258.9480206634 |
| 1991 | 13912.233169057 | 11147.3069425992 | 7802.51417208367 | 6314.0646730646 | 10627.2838045538 | 10483.2911097859 | 8565.64073327641 | 9027.08485029137 | 10459.7015068834 | 8751.9719388853 | 11003.3131710348 | 8992.09798621774 | 10356.8674209268 | 10501.7343844454 | 10638.5134749217 | 10759.87815559 | 10595.1773985309 | 8828.28577607868 | 10611.3863647697 | 10908.9538605798 | 11150.2184582174 | 8983.99271540907 | 9163.31938957428 | 10713.2017934521 | 10553.5965132094 | 10317.6027081916 | 10723.8766301014 | 8996.62232288165 | 11311.3019973497 | 8637.38066948879 | 8767.50225673608 | 8794.99975583833 | 10371.7639072478 | 10504.7980877584 | 11268.0126697241 |
| 1992 | 13887.7833217995 | 11127.7411432621 | 7787.61229928938 | 6312.59613447384 | 10616.254863391 | 10493.607769283 | 8564.93003569265 | 9047.00082189576 | 10459.066005743 | 8774.42627050918 | 11016.496521657 | 8983.507187484 | 10357.3058108902 | 10447.3310666595 | 10602.6559054967 | 10796.5242184093 | 10591.3103388714 | 8832.4862447555 | 10612.6327215381 | 10928.1167164603 | 11148.4050079087 | 8993.10159927014 | 9185.90516730372 | 10749.6762992468 | 10570.8809583093 | 10338.1691044218 | 10741.7443623569 | 8995.77944076376 | 11339.5829891531 | 8629.03423617088 | 8772.24250125595 | 8792.15397729079 | 10325.305726558 | 10504.6919374216 | 11277.4627012367 |
| 1993 | 13939.5554368334 | 11135.6727201298 | 7806.94585308819 | 6289.0607505764 | 10611.8889837949 | 10498.155120698 | 8559.58457436638 | 9062.57271801328 | 10458.9748304768 | 8794.31270676279 | 11026.8499189369 | 8983.47114703739 | 10358.772546982 | 10402.7900343745 | 10577.3350522513 | 10830.2723103781 | 10588.5159106509 | 8832.9681533733 | 10617.0816324221 | 10939.076905837 | 11144.7255113785 | 9002.83534540262 | 9208.49742084673 | 10783.8274719835 | 10584.1682287954 | 10358.0941390323 | 10751.4625298946 | 8990.92532665936 | 11366.4299476409 | 8623.56490178701 | 8778.40913732832 | 8791.22735690561 | 10288.74713881 | 10504.1308566682 | 11287.5065818483 |
| 1994 | 13888.8709298505 | 11145.8970434343 | 7805.83490112282 | 6288.69830100441 | 10608.4479814716 | 10502.6511894015 | 8560.79977608524 | 9076.75157401021 | 10462.8415134065 | 8806.35536946072 | 11035.4898458737 | 8985.92635778426 | 10357.5079460025 | 10366.5291942859 | 10557.8567746703 | 10850.7015165066 | 10589.6131624042 | 8826.93208664406 | 10619.9621563224 | 10948.0249882175 | 11136.0947635241 | 9008.23899492034 | 9221.50518584579 | 10809.2224961322 | 10595.7465985686 | 10372.3834572912 | 10757.1907837074 | 8985.92666939689 | 11379.0780886523 | 8615.04250536001 | 8783.65029697049 | 8789.55543085325 | 10263.5799861913 | 10498.432236248 | 11293.3958369498 |
| 1995 | 13945.1591051815 | 11148.7917752214 | 7806.24614025648 | 6304.00062743893 | 10609.7248023776 | 10500.9918260503 | 8561.45328923132 | 9084.49521177921 | 10465.1953400624 | 8812.84249134787 | 11036.159454567 | 8984.05902479004 | 10355.5711130197 | 10345.5515278711 | 10549.1250193165 | 10858.952135334 | 10592.5710616429 | 8825.02434689185 | 10622.1817222317 | 10956.3231347072 | 11120.7256761964 | 9005.98729619587 | 9225.58164364091 | 10829.5555618552 | 10603.383220352 | 10381.8690567856 | 10750.1049662189 | 8979.38123487808 | 11391.2962792562 | 8609.33647534432 | 8791.9535859562 | 8790.51068955401 | 10250.890963073 | 10495.6157694106 | 11300.0289022603 |
| 1996 | 13891.7065779656 | 11137.2438325531 | 7814.01861616106 | 6296.76462971346 | 10612.8261588323 | 10504.191382135 | 8558.13711055287 | 9078.61945226933 | 10464.5772133307 | 8797.15387971857 | 11026.8419687472 | 8986.02100810425 | 10351.8080729249 | 10344.1035762943 | 10557.4670301939 | 10858.1054477468 | 10625.1351415087 | 8809.15488434392 | 10640.00871346 | 10953.1717012445 | 11094.5688694395 | 8994.65529559948 | 9221.19982490937 | 10845.5107915861 | 10608.9577160669 | 10398.1596321269 | 10728.0523113243 | 8946.6140994886 | 11405.4325915665 | 8603.53534635541 | 8795.9084355546 | 8791.79277880905 | 10246.6509618568 | 10488.2008686409 | 11308.8425996531 |
| 1997 | 13943.6171545831 | 11160.2953772312 | 7863.32592663755 | 6283.73871011408 | 10607.896298629 | 10517.9746111377 | 8549.79171553043 | 9049.14942687379 | 10463.8169847517 | 8776.89446347622 | 11010.8811679516 | 8994.00469583678 | 10346.4687517214 | 10369.9443811791 | 10580.3921263591 | 10854.6024595596 | 10694.4857497406 | 8785.09536797419 | 10681.2461955481 | 10949.9552976767 | 11058.3552827549 | 8990.86831649871 | 9211.32132619332 | 10864.194972311 | 10615.6297330977 | 10420.0728643961 | 10680.4798875878 | 8878.08614985882 | 11429.8719432087 | 8589.56072239884 | 8804.11225489333 | 8792.7913459909 | 10239.6632802595 | 10479.265155451 | 11323.7645597499 |
| 1998 | 14022.9099824563 | 11145.868812069 | 7897.83922194255 | 6271.26935433171 | 10603.6979582187 | 10537.1362789804 | 8534.00269553125 | 9010.203268039 | 10463.9485745588 | 8747.08191423331 | 10988.8735671694 | 9007.9766117225 | 10341.7909392714 | 10399.8129953802 | 10611.6844229377 | 10851.0059726169 | 10778.8944947932 | 8757.82511985609 | 10727.8260139542 | 10944.1952309593 | 11021.8966237949 | 8990.25558591713 | 9204.60915223346 | 10879.7233813233 | 10623.4246733769 | 10443.197365463 | 10622.102764398 | 8796.18356177641 | 11452.8140210022 | 8573.04741101278 | 8804.2944295701 | 8796.89457261013 | 10233.8729530637 | 10474.2016568447 | 11341.6633752484 |
| 1999 | 14086.8368970586 | 11151.2983045329 | 7914.34626770853 | 6263.23645984549 | 10599.8609522685 | 10552.7856086982 | 8521.73787828988 | 8974.68983971312 | 10463.5930797258 | 8722.12558523729 | 10974.1499902758 | 9014.77026526871 | 10337.5274886591 | 10426.5451627005 | 10637.0770088105 | 10851.9441212923 | 10852.4708255658 | 8732.40923563972 | 10764.4851068929 | 10942.0005733987 | 10985.8008418551 | 8984.10613997692 | 9196.70393078529 | 10891.5091124645 | 10630.9659123753 | 10466.2083505541 | 10571.290271672 | 8723.76773024509 | 11474.1331827573 | 8556.73570412538 | 8807.60258299982 | 8799.86893310964 | 10226.9825478647 | 10467.0199831955 | 11356.2433104527 |
| 2000 | 14048.1296371707 | 11113.9349460565 | 7917.1917159029 | 6267.0402377144 | 10595.38634163 | 10563.2550350493 | 8517.61286196091 | 8955.24916764775 | 10460.4422865547 | 8708.47626489218 | 10953.3693251435 | 9026.01113891638 | 10334.8976826147 | 10438.0800320812 | 10650.1933109203 | 10854.4994465531 | 10884.8886470093 | 8718.76224872641 | 10776.3809967862 | 10946.8845366904 | 10963.9007083331 | 8982.40253494397 | 9195.88962850411 | 10897.680251925 | 10636.0400027785 | 10472.7157465698 | 10546.1778242218 | 8682.19919819505 | 11485.0695288006 | 8546.3704489135 | 8808.88104178144 | 8798.06544901715 | 10224.000262996 | 10461.924285863 | 11361.2058729468 |
| 2001 | 14089.3532161692 | 11097.9267161395 | 7912.89648401292 | 6267.49606259403 | 10598.3816483815 | 10574.3040909658 | 8520.35247366626 | 8953.25513592468 | 10456.8428207553 | 8702.65772124287 | 10939.0475670534 | 9025.95101780494 | 10336.4865331555 | 10421.8708258484 | 10657.0601189268 | 10868.9621315651 | 10894.1148803425 | 8720.52680336413 | 10770.4072214266 | 10959.6336622803 | 10959.0572345087 | 8984.93147095382 | 9208.18427599835 | 10889.2658291669 | 10633.5949908696 | 10462.6105997228 | 10518.9849703745 | 8665.55658360767 | 11477.8839925246 | 8546.65176637636 | 8811.1667568547 | 8799.99554120547 | 10223.6685724392 | 10453.8000729941 | 11356.0102598961 |
| 2002 | 14020.8110513627 | 11128.7450668906 | 7896.27991092951 | 6293.23271203361 | 10609.9243546079 | 10597.5616429797 | 8526.23435500313 | 8952.9255448134 | 10453.6835704952 | 8710.18156770246 | 10918.8381986765 | 9015.09033513987 | 10342.2896312305 | 10392.5739796661 | 10663.9246646524 | 10898.8641221298 | 10904.660529265 | 8727.51808934943 | 10757.7882200791 | 10982.2175514344 | 10969.6983907342 | 8983.79411409192 | 9223.79815925154 | 10871.5850633679 | 10634.0335151209 | 10436.6112996468 | 10469.7330527562 | 8650.1788927369 | 11459.2669317332 | 8553.55177655971 | 8805.15675252758 | 8796.66232441891 | 10224.1436999353 | 10444.7765827326 | 11346.1861576844 |
| 2003 | 14034.7887217879 | 11105.3878545894 | 7850.63965095888 | 6288.42338164584 | 10622.9881050266 | 10619.0224432631 | 8532.55763293227 | 8953.67592690267 | 10450.9150807513 | 8720.99579395525 | 10894.2582747694 | 9001.73700500689 | 10350.1942958397 | 10358.5841887377 | 10670.3923334672 | 10931.2218287758 | 10913.8366593717 | 8735.99506756007 | 10741.8540767845 | 11010.1841592772 | 10986.0050726187 | 8988.25940449477 | 9243.22266450998 | 10848.1285734475 | 10633.9897476003 | 10401.7386468128 | 10411.5008557215 | 8638.4747003219 | 11438.382854145 | 8564.25548046093 | 8798.42550923004 | 8793.71872143769 | 10234.6954579132 | 10435.2229653134 | 11333.9220068941 |
| 2004 | 14024.5620452189 | 11146.0341260154 | 7851.42110155647 | 6316.36909688166 | 10627.9362846713 | 10640.8930286222 | 8541.25538989249 | 8948.42596749242 | 10447.409439709 | 8727.81666152353 | 10872.0445191748 | 8985.53163966422 | 10354.334714157 | 10333.6180327097 | 10675.7307893757 | 10952.8725749135 | 10920.0368491374 | 8739.35778212177 | 10724.2727257142 | 11032.5844726421 | 10990.1539382285 | 8989.57546251544 | 9257.47431114363 | 10830.3931552875 | 10632.0884387053 | 10374.3701560392 | 10367.1838533681 | 8629.80246648673 | 11419.3667428704 | 8567.24299609423 | 8791.20593101076 | 8789.80145049041 | 10243.9273026886 | 10431.1925389166 | 11318.8079185046 |
| 2005 | 14014.0345302987 | 11092.6140649617 | 7849.5335567167 | 6308.47334275157 | 10628.0799765176 | 10660.9178704815 | 8547.29290781721 | 8940.47794964766 | 10447.1210974857 | 8733.83476933977 | 10855.7552710381 | 8972.45468349447 | 10357.3853448875 | 10319.5802525216 | 10681.2424441037 | 10961.5934891355 | 10923.0179958129 | 8739.2444218588 | 10710.0039889362 | 11043.5107333631 | 10982.8482522316 | 8993.98981215119 | 9261.57591190042 | 10821.4860417407 | 10626.7354543422 | 10356.0204315681 | 10351.151556619 | 8625.56349532386 | 11415.5884108852 | 8567.57332178523 | 8787.20600547338 | 8773.68322203758 | 10263.2360015504 | 10421.1642744919 | 11309.1725567718 |
| 2006 | 14004.5609430269 | 11166.6108229375 | 7811.74404205042 | 6334.75623285689 | 10606.9777480667 | 10675.2513206721 | 8551.24556387609 | 8925.69585150514 | 10446.5039845294 | 8735.47291577534 | 10848.4881461005 | 8960.57855524312 | 10355.5069810074 | 10323.1968311621 | 10682.8360757264 | 10958.3609169279 | 10914.8610668835 | 8734.10889669152 | 10692.0267016772 | 11052.0312362807 | 10957.1765760173 | 8996.8067559961 | 9245.55933260404 | 10820.8788150303 | 10623.2039211197 | 10342.6924704511 | 10365.9152265597 | 8617.82099166715 | 11419.6087925813 | 8555.87812222873 | 8788.70371238473 | 8746.17168216925 | 10301.2271657638 | 10422.3888465409 | 11288.4281072636 |
| 2007 | 13986.069667572 | 11177.8576772445 | 7776.35111621205 | 6320.21068794939 | 10571.314655471 | 10701.7438573469 | 8553.96998599702 | 8909.66059757329 | 10443.1732518777 | 8740.70884453563 | 10846.7561865969 | 8945.21804895854 | 10351.5689390289 | 10346.6905448445 | 10676.5503340433 | 10958.9902065552 | 10904.5947605361 | 8731.16616795984 | 10664.2810598135 | 11062.2296853645 | 10925.693322515 | 8986.75425625416 | 9215.23188117057 | 10811.8307554529 | 10617.1081900177 | 10326.2347991027 | 10394.6733465533 | 8601.39211455717 | 11423.0236276279 | 8538.0669681356 | 8794.91955440258 | 8712.36585352205 | 10356.5083366253 | 10425.3685754475 | 11249.371134908 |
| 2008 | 13907.8527674796 | 11183.0179063647 | 7753.88693646204 | 6318.4926802116 | 10530.5344192529 | 10720.7583456982 | 8555.88108371538 | 8893.5103922698 | 10440.7521585768 | 8749.44231195241 | 10840.1257283789 | 8926.21929566351 | 10346.8867724536 | 10376.1826261049 | 10668.8379031489 | 10959.8714320864 | 10885.6143327141 | 8725.21316944147 | 10633.3904623104 | 11082.7343142686 | 10892.0907597109 | 8969.13965529102 | 9178.71924158178 | 10802.8664249754 | 10608.9128437157 | 10310.2488483883 | 10419.8900380197 | 8581.80411874057 | 11422.1397980032 | 8519.3632908567 | 8799.94927209294 | 8675.01342449487 | 10416.7234132789 | 10434.2005173363 | 11206.0308872904 |
| 2009 | 13924.970260699 | 11206.2728699233 | 7775.5803830091 | 6308.80314560522 | 10497.6579867236 | 10741.8262804065 | 8559.13717502286 | 8873.77730055338 | 10434.2389685367 | 8759.76731070371 | 10835.8580580162 | 8909.69374986662 | 10343.730123242 | 10405.9560530662 | 10660.0012236387 | 10962.1192818571 | 10872.8705416314 | 8719.79221159105 | 10605.942075877 | 11094.2418342687 | 10863.9572737526 | 8961.92491508542 | 9139.81503173346 | 10797.4386017759 | 10602.7540401052 | 10294.3689131393 | 10441.9014278559 | 8566.26939818924 | 11417.7100545231 | 8506.65852871567 | 8805.36147728189 | 8643.94898857245 | 10467.2050506787 | 10440.1723916126 | 11168.1034518375 |
| 2010 | 13891.0418250865 | 11186.3321720249 | 7738.85975532322 | 6289.13527836824 | 10482.5038374739 | 10752.1945249453 | 8564.87591376368 | 8868.94338351926 | 10429.7787554236 | 8773.46931151842 | 10828.9422292003 | 8899.00482292959 | 10343.9411695408 | 10425.3016108188 | 10651.1220646787 | 10965.4259972895 | 10864.9180539573 | 8714.75292972264 | 10588.0100234539 | 11093.3518145598 | 10844.0464931274 | 8959.43455542511 | 9124.52135797686 | 10797.9522490995 | 10599.9892673828 | 10286.3897208079 | 10448.5474570606 | 8557.01078156343 | 11408.4112043212 | 8500.20238228726 | 8802.77365693499 | 8625.96293646312 | 10496.8734931924 | 10442.1593733929 | 11154.5148102024 |
| 2011 | 13835.8940785011 | 11169.7262513213 | 7732.66981429982 | 6297.83626350744 | 10492.8983394804 | 10753.0048095676 | 8570.19982123226 | 8870.43353206313 | 10426.4516080242 | 8792.91785005113 | 10821.3450826627 | 8899.75073552944 | 10348.6021208328 | 10430.9565530067 | 10640.6098016108 | 10974.798859797 | 10839.7034253646 | 8712.86919101528 | 10569.4633649077 | 11086.9486663532 | 10832.1888030765 | 8966.04951280397 | 9124.94331946306 | 10799.1610954586 | 10605.7653640874 | 10303.5189306985 | 10431.5640699873 | 8546.85141505569 | 11364.1005391275 | 8501.2618628104 | 8799.87257893027 | 8617.68428294555 | 10524.6867189144 | 10448.8502557906 | 11158.7856740747 |
| 2012 | 13825.0946642325 | 11176.2952145379 | 7714.9873300496 | 6319.44437866311 | 10513.0864121201 | 10743.7304126375 | 8577.68988687253 | 8873.86365581942 | 10424.0703905401 | 8816.47005595334 | 10818.5899875248 | 8898.68097255132 | 10352.3549237722 | 10430.9262317885 | 10620.5109985041 | 10993.7215165274 | 10782.899353673 | 8708.27412914574 | 10540.6106442292 | 11069.9000268638 | 10815.8995812222 | 8973.96168286714 | 9130.52982373659 | 10794.8650401624 | 10619.183973014 | 10339.1661530119 | 10395.3777596002 | 8522.595718486 | 11272.009522009 | 8508.85391422668 | 8788.42709629129 | 8611.95217902039 | 10557.9749728586 | 10460.1216007454 | 11168.0053064634 |
| 2013 | 13776.038902551 | 11167.8663084807 | 7706.7951895042 | 6328.00506358927 | 10538.2768632365 | 10730.9283317444 | 8587.96025718781 | 8879.63716516239 | 10425.652099982 | 8842.02841940815 | 10817.1449116331 | 8903.26253547029 | 10355.8845908339 | 10423.7156580706 | 10594.2682941858 | 11018.6857743853 | 10711.4404765275 | 8704.89987022115 | 10510.421965466 | 11048.4386065069 | 10798.4854000846 | 8985.14958074279 | 9139.03101331371 | 10790.9043349535 | 10635.153815425 | 10381.4217746824 | 10359.6808842353 | 8491.87670557201 | 11164.21156259 | 8516.47172722682 | 8780.30254832837 | 8605.05207176483 | 10594.9885482459 | 10473.024609089 | 11178.0547138157 |
| 2014 | 13776.5901441558 | 11168.8834247178 | 7675.44044795194 | 6323.8582194912 | 10564.1585286808 | 10715.3410916451 | 8597.48608128926 | 8887.51475105949 | 10426.5654875246 | 8863.08401282509 | 10810.4655216967 | 8905.82604918474 | 10358.1885665942 | 10415.6894101184 | 10570.9464836064 | 11035.3260003317 | 10652.0133768147 | 8702.37840980459 | 10484.1337327306 | 11031.7620762055 | 10783.1388912154 | 8991.62600244981 | 9143.60041193937 | 10784.8166409996 | 10649.8568173405 | 10420.5098447562 | 10327.5668136678 | 8464.9750398773 | 11073.9452528564 | 8526.17553058137 | 8772.07826833 | 8599.25796590985 | 10624.2401317197 | 10480.0220394244 | 11185.3295894189 |
| 2015 | 13758.2637957749 | 11182.6054420226 | 7660.8583199922 | 6345.57362693921 | 10576.8325751665 | 10709.5832354724 | 8605.64489654026 | 8897.8672843952 | 10424.8926394558 | 8885.29031555617 | 10805.003234473 | 8906.30324831239 | 10363.5838110912 | 10410.1655354698 | 10559.6057943347 | 11049.5852744086 | 10630.1546587086 | 8704.77458197953 | 10471.616915646 | 11033.5503349233 | 10781.1029868703 | 8995.70835303309 | 9147.40531652567 | 10779.7468660472 | 10660.8166458142 | 10439.0371990532 | 10316.4969199337 | 8451.98484978436 | 11031.0876922763 | 8528.24629902422 | 8773.41261848073 | 8601.08936005516 | 10641.2600662129 | 10480.2239532057 | 11187.7318742144 |
| 2016 | 13769.2574685969 | 11222.5068894384 | 7668.94838331796 | 6357.83934373919 | 10572.1519533406 | 10703.4379605106 | 8613.43336751003 | 8898.5454709257 | 10414.2588164705 | 8895.82141130459 | 10785.1838602379 | 8909.35545160089 | 10363.8863891814 | 10397.6024459894 | 10556.8808842466 | 11026.4963799796 | 10642.810975309 | 8692.11807796646 | 10457.4886063429 | 11068.9511910216 | 10802.2381834708 | 9001.45268659497 | 9118.9093226154 | 10769.8835636197 | 10669.7623584369 | 10444.3300838125 | 10364.7635714051 | 8435.66446747807 | 11055.0580723636 | 8526.95574629696 | 8771.06728433303 | 8605.35461797432 | 10645.1262783669 | 10466.3256824767 | 11179.7233466873 |
| 2017 | 13718.8179655639 | 11223.509128962 | 7639.80990088957 | 6348.87955087915 | 10569.6725983508 | 10702.5206696894 | 8624.41628863063 | 8908.23146409467 | 10407.4847764539 | 8902.34693492179 | 10771.4397117495 | 8912.56615406802 | 10366.8678558812 | 10391.1544754467 | 10560.1477219378 | 10999.5707620014 | 10661.0907651448 | 8688.71058754265 | 10450.9078275188 | 11110.9905232535 | 10830.7606565218 | 9007.94662967178 | 9087.21318955062 | 10767.8643167606 | 10678.9549390314 | 10445.6639730687 | 10412.1695733248 | 8423.25717898695 | 11087.707681544 | 8524.98482304411 | 8769.01885501324 | 8614.55951370905 | 10656.9488460896 | 10453.7435221376 | 11171.8439910777 |
| 2018 | 13723.474057235 | 11224.9207835512 | 7656.12909174755 | 6366.88485800233 | 10569.6436975238 | 10714.9893617889 | 8635.11277365875 | 8938.73135130958 | 10445.9368446468 | 8926.28138195409 | 10785.9227851735 | 8923.94210138082 | 10382.1501188665 | 10434.0552772563 | 10571.8224525472 | 11006.25066423 | 10705.4796340314 | 8712.66064687648 | 10474.9276843976 | 11114.4684685568 | 10835.0747822351 | 8997.27767795155 | 9062.78518413117 | 10796.2323528476 | 10685.5067037077 | 10451.0753434489 | 10523.4108812267 | 8446.20705163158 | 11098.1210285875 | 8519.8243496549 | 8763.83962675307 | 8624.25763519965 | 10666.494097505 | 10474.7494695785 | 11195.1847541282 |
| 2019 | 13816.0195747335 | 11233.4998426855 | 7717.63257649739 | 6372.33289323917 | 10568.0573629468 | 10744.1514557426 | 8650.07998424214 | 8989.02565707966 | 10531.8719793091 | 8962.329564144 | 10835.0098248713 | 8940.88916263488 | 10411.1113373545 | 10519.894034808 | 10601.1131021522 | 11031.1709650581 | 10782.1385816214 | 8755.86285099407 | 10521.8298414533 | 11104.2741889443 | 10830.5573512469 | 8965.96607628735 | 9013.11141874264 | 10857.7379462648 | 10696.6100746807 | 10458.3474848418 | 10753.7420602786 | 8499.84492152755 | 11108.7143972444 | 8505.07396211614 | 8750.87175517345 | 8637.15907371554 | 10677.3014652575 | 10524.0772353824 | 11256.9159587479 |
### Chart: Age-standardized YLL rate in females
| Category | z Bulg up | z Bulg low | z Ice up | z Ice low | Austria | Belgium | Bulgaria | Croatia | Cyprus | Czechia | Denmark | Estonia | European Union | Finland | France | Germany | Greece | Hungary | Iceland | Ireland | Italy | Latvia | Lithuania | Luxembourg | Malta | Netherlands | Norway | Poland | Portugal | Romania | Slovakia | Slovenia | Spain | Sweden | United Kingdom |
|---|---|---|---|---|---|---|---|---|---|---|---|---|---|---|---|---|---|---|---|---|---|---|---|---|---|---|---|---|---|---|---|---|---|---|---|
| 1990 | 16153.104631978 | 14700.7094873242 | 8485.2560122668 | 7684.20837433876 | 9483.96329170215 | 9035.11065580523 | 15432.2488248533 | 12096.479152955 | 12465.0622184766 | 12843.5951085613 | 10577.997785768 | 13161.0211326798 | 9930.24378231313 | 8709.2250926545 | 7188.24998024729 | 9929.29312523577 | 8823.9249043906 | 15001.9957742666 | 8079.28167578672 | 10702.5138023604 | 8356.49116193657 | 13388.4348190635 | 12103.4848971273 | 9698.49474583202 | 9525.14450726414 | 8608.45280955945 | 8386.13843150484 | 13089.9977597084 | 10405.4179564312 | 14933.3383987703 | 12525.636852826 | 10635.4842087854 | 8101.17724217081 | 7895.48911135734 | 10113.0201190565 |
| 1991 | 16026.5553931763 | 14597.4230706502 | 8415.46744826703 | 7638.54872952695 | 9358.24812200764 | 8830.66626361658 | 15327.8883058689 | 11774.3890376672 | 12233.6743922215 | 12584.8630341429 | 10446.710089693 | 13523.0340435037 | 9786.86799727281 | 8503.40874693617 | 7052.40790373724 | 9731.37571134445 | 8684.56899166609 | 14912.9557885932 | 8025.96404968019 | 10397.9213139376 | 8237.64342778262 | 13291.8808256144 | 12610.8728203859 | 9495.74626322658 | 9308.47891431504 | 8579.51316790521 | 8235.29617240412 | 13298.4939562484 | 10324.2827752612 | 14684.6197908314 | 12463.8956556854 | 10395.9175893413 | 7933.73501542045 | 7808.21140191465 | 9872.56638588815 |
| 1992 | 16204.1234281645 | 14786.1169932086 | 8391.75002656956 | 7549.94794353543 | 9241.20769950755 | 8735.28480732363 | 15488.053642553 | 11585.0959557742 | 12149.1951043483 | 12100.2722266794 | 10466.8803722056 | 13044.2822990433 | 9587.53706537871 | 8402.05798657988 | 6893.50635546289 | 9447.53176700872 | 8673.32573617461 | 15184.2939998716 | 7967.1973352884 | 10259.537592646 | 8041.19120342843 | 13516.499951243 | 12395.9559601619 | 9282.74709576609 | 9161.15411226446 | 8493.35406403281 | 8127.73821932925 | 12963.7007881474 | 9958.54139587013 | 14866.9360247189 | 11916.3066878813 | 10130.3807301531 | 7654.53568420707 | 7665.67727074464 | 9658.4562156146 |
| 1993 | 16344.7088098322 | 14920.7139410386 | 8324.07990021934 | 7512.85024650239 | 9096.22332849892 | 8572.80535404168 | 15640.2461257355 | 11352.2787467189 | 12045.7554529998 | 11753.9710314628 | 10486.1923295113 | 13802.6516199283 | 9448.03422056863 | 8225.52203544081 | 6788.23100170528 | 9318.09463852827 | 8478.23252029973 | 15251.4688779755 | 7895.09139005096 | 10035.1655901295 | 7907.33637376609 | 14712.9951962135 | 13287.5292997811 | 9058.93672357599 | 8897.36141828958 | 8516.24651806848 | 8019.63392122456 | 12496.723125099 | 9857.39446175735 | 14836.714133656 | 11714.2050273383 | 9881.93308953406 | 7469.84277102621 | 7521.38665652319 | 9537.84699543709 |
| 1994 | 16728.1823604791 | 15314.9611553019 | 8223.95594047266 | 7403.97600399037 | 8852.24922788932 | 8438.05556357102 | 16086.1876040932 | 11207.9123991933 | 11731.721827931 | 11399.8953216987 | 10470.5955610981 | 14583.9781323745 | 9262.89155903274 | 7869.64630899606 | 6596.39130420587 | 9073.79926243716 | 8345.50402446119 | 14699.6682393488 | 7807.11651475356 | 9838.9344297116 | 7738.93015110955 | 15622.7471053319 | 13591.6450947247 | 8761.57648054311 | 8680.85780688896 | 8399.58270848949 | 7974.3355002737 | 12468.9448056848 | 9375.3172605676 | 14801.672788553 | 11337.269306966 | 9611.29959370684 | 7275.65829852418 | 7231.59770031891 | 9264.76484880459 |
| 1995 | 16816.095464132 | 15378.2778503129 | 8112.89847375196 | 7360.17817988298 | 8609.42994738423 | 8222.37272195403 | 16098.2915982955 | 11087.9913961755 | 11351.4833704655 | 11543.7036210623 | 10465.0585327988 | 13636.6314398115 | 9113.35202579327 | 7741.98226448539 | 6503.49672951975 | 8869.83426307218 | 8246.18001479324 | 14455.5281959318 | 7722.49876139606 | 9809.92272734915 | 7555.05338535296 | 15044.4542778129 | 13278.6467623554 | 8555.69187966918 | 8467.99024760494 | 8291.66590716064 | 7693.87924562743 | 12185.7952908096 | 9213.08770775379 | 14962.2051421351 | 11385.919651123 | 9336.17001267638 | 7096.8488730074 | 7132.19243511736 | 9148.82005007087 |
| 1996 | 17020.0119615102 | 15553.9091033831 | 7969.51079528696 | 7199.73666831871 | 8462.47093576733 | 7978.17497661929 | 16347.4957137138 | 11201.3975052018 | 10797.5529973009 | 10717.840942381 | 10225.8781599952 | 12398.7896332281 | 8926.30520200911 | 7548.96955233094 | 6427.79165302275 | 8673.12239482827 | 8115.98418064299 | 13656.1293372903 | 7582.94631102482 | 9541.95303018237 | 7391.26530314089 | 13281.5282877944 | 12383.3887070184 | 8441.25836735018 | 8220.40390702781 | 8262.57612837401 | 7634.71849176716 | 11868.7968086392 | 9031.08192106463 | 15325.3281456154 | 10950.8963848886 | 9253.67006772074 | 6949.30660264867 | 7058.25424421478 | 8975.2981982861 |
| 1997 | 17664.9462481274 | 16153.074132844 | 7839.88640245247 | 7081.13421458894 | 8147.38395645307 | 7843.46956743944 | 16948.6276254247 | 11205.1699208716 | 10420.4795827328 | 10716.8301790715 | 10061.9919638386 | 12127.1049947102 | 8759.34157639973 | 7542.38369085735 | 6332.90279127909 | 8438.87472585176 | 7958.67518261333 | 13605.1210055692 | 7455.67056209834 | 9479.03731582545 | 7229.46441502562 | 12891.1749740539 | 11566.7299162887 | 8269.44380922804 | 8008.79313452942 | 8169.24595326142 | 7543.841347954 | 11602.5842157861 | 8858.96385252952 | 15006.6341166766 | 11402.6112611102 | 9050.64422357012 | 6762.95554576291 | 6931.45947322216 | 8790.3574430107 |
| 1998 | 16839.9956543551 | 15405.2462545269 | 7641.31611870546 | 6885.38315190192 | 7992.11463846271 | 7747.64734708893 | 16106.2353533907 | 10954.8070204758 | 10242.8235273061 | 10182.0326249916 | 9741.22841513461 | 12563.274092193 | 8589.46020703811 | 7363.50363680424 | 6345.88109456314 | 8221.10324688902 | 7920.57854053085 | 13563.8333730695 | 7266.65807335612 | 9272.66126150976 | 7154.55369226565 | 13302.443561885 | 11461.5939552427 | 8109.28057930462 | 7861.39393485885 | 8086.02217323997 | 7474.73653633457 | 11219.8632223593 | 8639.01137697566 | 14516.9704224339 | 11119.7934945606 | 9001.7733507516 | 6636.92883906638 | 6891.29580639925 | 8673.39391953803 |
| 1999 | 16489.0010172275 | 15086.6142552433 | 7405.54829184008 | 6658.77784237852 | 7847.5757794989 | 7537.85230415609 | 15814.3410369573 | 10620.5226192118 | 10025.0340937023 | 9909.36940624986 | 9816.68272753791 | 12026.4374123053 | 8420.60513640888 | 7230.40533617757 | 6289.13381583913 | 8017.64273819188 | 7814.65246244431 | 13443.7319225341 | 7042.86036429304 | 9199.4723424258 | 6935.50109544111 | 12636.7731484216 | 10971.3985220097 | 7977.80610043718 | 7849.97887704692 | 8130.54869142858 | 7427.2222402823 | 11068.687083706 | 8430.89053110394 | 13898.0360248893 | 11026.7789662184 | 8905.21273151974 | 6531.49630315784 | 6846.90100663074 | 8576.19150612179 |
| 2000 | 16311.428934707 | 14939.3912877879 | 7234.31201664411 | 6447.9798177521 | 7630.5208514517 | 7397.87164063023 | 15634.9938911438 | 10165.0106238225 | 9410.94291359151 | 9825.75070438986 | 9408.67220776815 | 12057.14567723 | 8191.37983570676 | 7177.45995047983 | 6192.42523606978 | 7809.14926770078 | 7613.34680416891 | 12801.2235202342 | 6844.59511368428 | 8961.42903141864 | 6738.26019146262 | 12090.8875598925 | 10607.0383296787 | 7872.98729239844 | 7762.20352123283 | 8054.5388264087 | 7320.40677674595 | 10610.7253580977 | 8149.42368820618 | 13392.0609691823 | 10520.1223167684 | 8461.33280446035 | 6310.11715829583 | 6785.92440799775 | 8374.13307922116 |
| 2001 | 15962.226441212 | 14602.0253265771 | 7066.75092193516 | 6223.30030574504 | 7386.78783145888 | 7222.1637133135 | 15269.0091858727 | 9844.12000763813 | 8944.75122899273 | 9678.7212157439 | 9300.52793670474 | 12012.8836310592 | 8016.2925131869 | 6974.02241876964 | 6139.32813593688 | 7642.3026999579 | 7468.68722971863 | 12322.3428897025 | 6651.83277809498 | 8585.82107452938 | 6546.36405725037 | 12590.388928027 | 10821.4486916918 | 7682.62971308956 | 7586.18995584099 | 7976.33826603433 | 7203.0516967672 | 10228.3772029616 | 7897.19145330034 | 13359.3732880397 | 10579.0883314853 | 8358.19236511993 | 6141.40620018309 | 6693.05036667169 | 8172.78828113769 |
| 2002 | 15659.2884329491 | 14352.7866535981 | 6919.04386244001 | 6021.32110148961 | 7247.95062825376 | 7133.79468179832 | 15003.0007684662 | 9776.8965238109 | 8691.15020475663 | 9406.65998095234 | 9157.3080948343 | 11313.1519377538 | 7891.19737970998 | 6898.39589921423 | 6083.99665673447 | 7565.99588257261 | 7404.22281973786 | 12133.6102538201 | 6474.0075354766705 | 8197.62075076526 | 6433.23415708543 | 12174.8249314624 | 10535.1432434257 | 7447.44774283073 | 7505.00908264511 | 7910.45709301921 | 7126.48835572709 | 9878.64857040583 | 7696.66154149882 | 13409.9778359932 | 10225.4187573935 | 7912.4963562185 | 6041.60162252204 | 6603.70139398477 | 8090.03074908697 |
| 2003 | 15163.3174343251 | 13917.0234622377 | 6781.42080989259 | 5827.47857450993 | 7186.46503546783 | 7014.84846679259 | 14578.514853803 | 9753.90325692057 | 8519.99005900273 | 9526.09623345437 | 8859.71505983547 | 11002.5428091407 | 7772.45402742783 | 6783.4870101394 | 6001.85540646286 | 7448.09841388486 | 7269.22948506706 | 12156.6254057726 | 6304.46268811495 | 7993.30142004379 | 6347.65771157104 | 12265.3063221887 | 10566.4478382633 | 7176.07034280219 | 7406.96541768833 | 7714.1504680022 | 6905.89160025882 | 9648.97805741445 | 7474.06776867013 | 13156.3316744917 | 10203.3160616496 | 7568.36848069496 | 5992.62100775486 | 6486.80369962754 | 7990.12088117164 |
| 2004 | 15033.4984796377 | 13726.547622835 | 6615.76890966446 | 5645.08309618904 | 6897.53925490407 | 6770.45813912682 | 14398.1457836899 | 9444.79561450226 | 8300.70038396381 | 8966.40976351696 | 8571.93459470805 | 10432.3644642096 | 7509.69260922181 | 6756.51851027063 | 5766.7563133924 | 7175.92068804884 | 7142.17215313396 | 11796.0227739109 | 6112.5057012275 | 7721.69405246628 | 6077.40904600689 | 11925.7184628806 | 10376.0421526262 | 6986.47094532665 | 7132.63393480244 | 7448.88441846746 | 6733.82652488841 | 9479.5687249104 | 7062.1195443164 | 12560.0121578287 | 10121.921612778 | 7690.72865186745 | 5793.11538833774 | 6417.60772670977 | 7734.66369746369 |
| 2005 | 14879.0124304292 | 13638.2487004548 | 6402.17400570946 | 5453.38037822929 | 6755.62175875884 | 6644.76484469119 | 14263.8479453775 | 9250.11410340586 | 8103.37450592115 | 8924.99243013638 | 8307.02290244778 | 10317.2974336548 | 7365.20816157223 | 6613.86227215549 | 5633.63168437435 | 7002.03693863386 | 6958.35549891167 | 11743.0521013742 | 5916.59519867971 | 7463.93594599917 | 5938.15088494379 | 11882.8811874004 | 10830.6436440152 | 6870.36777001524 | 6751.03794968597 | 7245.45462459994 | 6530.17813027189 | 9356.10428771253 | 6833.7783561238 | 12454.9624789925 | 10018.5879992638 | 7312.23607018061 | 5676.15163904222 | 6216.10789103817 | 7567.91199869256 |
| 2006 | 14873.4255304717 | 13557.885464441 | 6242.15282990042 | 5287.94040302501 | 6527.86888920811 | 6476.24361405796 | 14206.4245261774 | 9010.93302619427 | 7943.80960711168 | 8504.03662796074 | 8129.70469659831 | 10011.8769315039 | 7189.18375957246 | 6373.51764347259 | 5531.36246230668 | 6835.98468372672 | 6810.87786874999 | 11294.4118225651 | 5720.72706660854 | 7320.74718761137 | 5786.0309022748 | 12517.504051616 | 11117.5084779924 | 6741.66707460532 | 6619.24881849981 | 7053.28276369661 | 6405.13549569886 | 9237.25665657659 | 6478.57589449185 | 11947.3793116952 | 9863.21042040328 | 6776.09034650053 | 5500.96142275022 | 6158.8209139072 | 7418.19333810817 |
| 2007 | 14472.3522709853 | 13267.9211850435 | 5958.52484543008 | 5067.99293141143 | 6412.53113334461 | 6449.10353752947 | 13845.1900247156 | 9132.07523601512 | 7690.88227513151 | 8242.9131406104 | 8027.93789726739 | 9782.43817507571 | 7072.39300611388 | 6299.90363416232 | 5421.56178425577 | 6740.52660182958 | 6819.01132629491 | 11410.6165400497 | 5486.86286043028 | 7045.64613115783 | 5704.75354020035 | 12010.3828069841 | 11248.9640280278 | 6584.67416170527 | 6471.43085679672 | 6848.46066495854 | 6358.76902156274 | 9137.2321692771 | 6361.53253723863 | 11436.1653050825 | 10009.6908296706 | 6785.12749380639 | 5413.50253872903 | 6051.56926546833 | 7294.78237650354 |
| 2008 | 14031.4245764076 | 12809.5322080668 | 5688.73100402847 | 4889.207174634 | 6321.34906700826 | 6345.33124703651 | 13423.8722859458 | 9071.85583800891 | 7427.40218485675 | 8165.68941217143 | 7826.43628370262 | 9260.56494117161 | 6952.06819198672 | 6205.60319432079 | 5400.40976197423 | 6681.9559365052 | 6599.49641642407 | 10881.1212421315 | 5273.45737618248 | 6952.60754225638 | 5623.28469824808 | 11142.532162871 | 11109.2740442763 | 6422.16206845988 | 6334.48023010765 | 6738.83254727177 | 6203.93755664977 | 8931.10838318661 | 6203.34331406778 | 11208.3524428362 | 9498.5830734 | 6482.54527276811 | 5329.54692010628 | 5928.45515603398 | 7193.62996341278 |
| 2009 | 13763.8601118649 | 12538.1730083232 | 5478.17862334914 | 4775.17017369327 | 6298.2699054671 | 6306.64238460184 | 13136.8879860909 | 8646.32774852131 | 7002.55714940966 | 7941.29149355368 | 7646.19497265559 | 8715.95567906605 | 6827.16579951333 | 6128.23486616077 | 5345.65164762343 | 6595.51076900518 | 6465.14616557286 | 10741.6553275734 | 5100.94640934005 | 6776.17352129743 | 5552.2470799277 | 10826.6346723099 | 9949.13177173562 | 6320.47477741957 | 6206.06708763237 | 6584.48845618862 | 6114.99044688669 | 8816.54352643589 | 6089.10541829938 | 11135.6859740888 | 9558.92046660207 | 6384.2177297198 | 5182.00442137095 | 5852.8032869945 | 6974.71805208538 |
| 2010 | 13739.7409988325 | 12526.4830856441 | 5326.53678916337 | 4670.35685767302 | 6167.04422499953 | 6207.52030840691 | 13123.763580104 | 8684.02688823406 | 6777.19730628293 | 7736.70339655417 | 7435.54108655292 | 8077.5854713364 | 6650.72514970947 | 6029.96933904377 | 5243.38812519378 | 6454.53624573565 | 6369.64355218773 | 10433.1131813874 | 4967.60853015538 | 6501.92958798725 | 5411.7075758509 | 10423.0683742755 | 9676.6790802669 | 6234.41003252608 | 5972.49318390845 | 6485.21974280247 | 5983.83655213862 | 8340.88360632257 | 5872.57428302904 | 10845.212808235 | 8997.5700135274 | 6241.65321050942 | 5027.62937094229 | 5739.49402632069 | 6833.2781034317 |
| 2011 | 13433.3881261661 | 12214.0478830884 | 5210.4526317715 | 4534.58370282812 | 6104.29687059192 | 6127.6226354967 | 12820.1475297154 | 8420.54121269973 | 6650.08064966343 | 7638.01681953176 | 7145.19600533986 | 8095.52046002836 | 6530.5705934142 | 5903.05666955598 | 5151.53594597348 | 6368.88098505912 | 6283.75600650849 | 10291.6901688303 | 4847.59782910189 | 6483.20705485888 | 5357.50696741402 | 10117.8520660306 | 9540.4373658349 | 6085.42024902011 | 5906.46682977959 | 6448.06121067684 | 5900.54477631653 | 8171.4719562349 | 5714.67454115908 | 10254.6098298618 | 8947.05444837623 | 6173.49811788443 | 4977.32460313786 | 5680.29761424867 | 6695.85465346589 |
| 2012 | 12958.4931627124 | 11777.1654743561 | 5097.78829083568 | 4440.45840291657 | 6003.17124405776 | 6048.15468995975 | 12371.2575139569 | 8066.81491970463 | 6496.24200861776 | 7503.65672355957 | 6940.53879202608 | 7852.52526603637 | 6436.31152161635 | 5860.09940836186 | 5086.49567820629 | 6267.7387068403 | 6231.61527694116 | 10068.1533333457 | 4748.95788986237 | 6353.52848508578 | 5291.53734190924 | 9563.65869669181 | 9312.76480567893 | 5919.42999994779 | 5807.69191894608 | 6369.66131924451 | 5788.36003839718 | 8139.59228210417 | 5551.1840495783 | 10154.2126367421 | 8686.58983148124 | 6094.13420892941 | 4889.17890477201 | 5617.94976915004 | 6634.03233103876 |
| 2013 | 12615.116100675 | 11487.0850589584 | 5058.49567761277 | 4372.50403263168 | 5912.63347804175 | 5985.42752820226 | 12029.1704860702 | 7890.15242652327 | 6385.10380947576 | 7332.04713057025 | 6720.60740968423 | 7744.78523177554 | 6324.70535490247 | 5718.36524034987 | 4996.97076872775 | 6276.69444993433 | 6053.55125887582 | 9792.29906073696 | 4688.50374050679 | 6231.24436144055 | 5175.92000458937 | 9767.69360194402 | 9267.53336995568 | 5852.85865896804 | 5671.99256562035 | 6239.41311113455 | 5697.95841995545 | 7941.99020198782 | 5390.41326389184 | 9726.68011469507 | 8522.77052198502 | 5837.89411014263 | 4781.91352511394 | 5537.91739625164 | 6580.26636794081 |
| 2014 | 12991.602680409 | 11821.1213696175 | 4983.46500938342 | 4309.72814010511 | 5855.42170877539 | 5782.77013172789 | 12418.5788996018 | 7834.64220357149 | 6400.90838318744 | 7024.83315025783 | 6541.34737622156 | 7539.52698738541 | 6214.35053901365 | 5586.08651327165 | 4901.70475834282 | 6135.65321261701 | 5883.22674246526 | 9644.94843262558 | 4633.13390070418 | 6067.80702763989 | 5072.52910134885 | 9280.71484478555 | 8845.25666616965 | 5667.10450688523 | 5587.34306945029 | 6142.62201323392 | 5484.22985506545 | 7708.53104990204 | 5248.9914885294 | 9836.8295476637 | 8265.23942968019 | 5443.25365685413 | 4721.16396540595 | 5444.01001351856 | 6517.59202514618 |
| 2015 | 12840.1179239544 | 11694.6757421946 | 4910.25130943517 | 4238.94975483025 | 5746.0334071425 | 5787.40730044126 | 12265.29359676 | 8070.46329245175 | 6333.41184627349 | 6993.14349798219 | 6463.72860634736 | 7339.00589420762 | 6210.27056304936 | 5453.13327384916 | 4891.39870880978 | 6192.64910536833 | 5848.51457322479 | 9833.78931409542 | 4567.88881534742 | 6040.88375909333 | 5111.30539287941 | 9007.03699660668 | 8920.45302431622 | 5488.00731730147 | 5578.35754676211 | 6119.97085184698 | 5358.48161419517 | 7639.98541479297 | 5164.06677362128 | 9647.88231235753 | 8386.28552179594 | 5487.73388919541 | 4725.90524192642 | 5347.00518482158 | 6528.87128934385 |
| 2016 | 12732.4801939015 | 11465.0017299018 | 4855.65511558553 | 4116.87502171775 | 5664.88444868739 | 5626.69641603512 | 12088.2731316733 | 7381.58063701457 | 6215.43963646277 | 6949.6886023777 | 6448.45681107098 | 7635.57103143129 | 6116.83190017814 | 5400.91663904985 | 4759.97772436152 | 6184.23583397714 | 5891.10487681885 | 9279.17861904975 | 4472.18675594483 | 5888.79850629254 | 4973.76478618653 | 8951.19891375218 | 8648.98455540617 | 5246.2490894833 | 5547.7543547125 | 6165.80882486319 | 5330.83302986167 | 7469.4913190025 | 5127.45253226202 | 9494.40542959402 | 8180.54973623992 | 5331.21874239962 | 4637.54181991334 | 5334.84129828945 | 6528.44013416425 |
| 2017 | 13719.5523243335 | 10561.9710581233 | 4767.85685483849 | 4021.12190315287 | 5511.19086044 | 5591.25327964949 | 12015.1758009817 | 7270.84631047995 | 6193.41755045735 | 6767.34443739472 | 6429.41623467299 | 7622.76440781435 | 6064.76437353802 | 5385.09145852276 | 4731.51667373886 | 6101.00114674182 | 5845.76245149629 | 9383.61813912383 | 4370.12638120152 | 5840.58129253686 | 4936.69376397776 | 8919.53553465105 | 8339.7509932952 | 5191.71679979437 | 5457.50840874888 | 6113.43351428022 | 5216.83798708028 | 7391.90248827922 | 5085.78881394552 | 9479.48927613951 | 8109.88912368789 | 5309.23656275453 | 4610.36561863245 | 5284.13731075231 | 6480.74037401256 |
| 2018 | 14473.1970545924 | 10121.084854273 | 4753.81376415382 | 3976.24053098195 | 5471.24070012545 | 5575.77867797869 | 12180.0932084694 | 7203.62082599139 | 6118.24851559237 | 6753.12472400873 | 6392.28352615653 | 7553.00966573236 | 6061.87674783675 | 5351.51295868466 | 4721.79209558144 | 6065.42587157955 | 5968.08126201196 | 9405.21012938964 | 4330.61385328112 | 5839.5427016734 | 4952.81177980549 | 8872.17856003767 | 8245.20055068808 | 5142.88998906526 | 5381.25138435553 | 6109.9683421568 | 5212.09889945325 | 7370.14276060249 | 5098.59304611507 | 9429.96733732675 | 8071.3385381278 | 5288.21096357547 | 4631.23206799866 | 5268.2431171602 | 6520.42155069789 |
| 2019 | 14975.2327757793 | 9860.96816933949 | 4743.78644088231 | 3908.99216401526 | 5441.14766596112 | 5570.16651929186 | 12171.9223761704 | 7171.53824276237 | 6056.99126708772 | 6679.52496377329 | 6359.31219589207 | 7473.16337347473 | 6038.94846899731 | 5324.77931350427 | 4721.21622784261 | 6045.78846259322 | 5994.83767105621 | 9232.9164727357 | 4293.85235710424 | 5791.75018541203 | 4940.47153261594 | 8837.50764588141 | 8195.24553222142 | 5106.36749568267 | 5303.03235299491 | 6086.47897098204 | 5139.80252550058 | 7320.73648449957 | 5081.60683629024 | 9415.22163367967 | 8017.72164124392 | 5272.63248786362 | 4623.93780068947 | 5182.4305063424 | 6508.64325264627 |
