# Supplementary material for: Inequalities in the burden of non-communicable diseases across European countries: a systematic analysis of the Global Burden of Disease 2019 study
Source: Int J Equity Health. 2023 Jul 28;22:140. doi: 10.1186/s12939-023-01958-8 (PMC10375608; doi:10.1186/s12939-023-01958-8)
Supplement: Supplementary file 2 — Additional file 2: For females, the UK had the highest annual rate of change, followed by Estonia and Finland. The countries with the lowest annual rates of change for females were Cyprus, Romania and Italy. In the EEA, the lowest annual rate of change for females was reported for CVDs at -0.54, followed by DDs at -0.26 and CRDs at -0.16. For males, the highest annual rates of change in DALYs were in Estonia, followed by the UK, and the lowest in Italy, Spain and Portugal. For males in the EEA, the lowest annual disease-specific rate of change was -0.55 for CVDs, followed by CRDs -0.39 and then DDs -0.32. Figure: Age-standardized level 2 NCDs DALY annual rate of change by EEA Member States, 1990–2019. Legend: CVDs: cardiovascular diseases. [file 12939_2023_1958_MOESM2_ESM.pptx]

## Slide 1
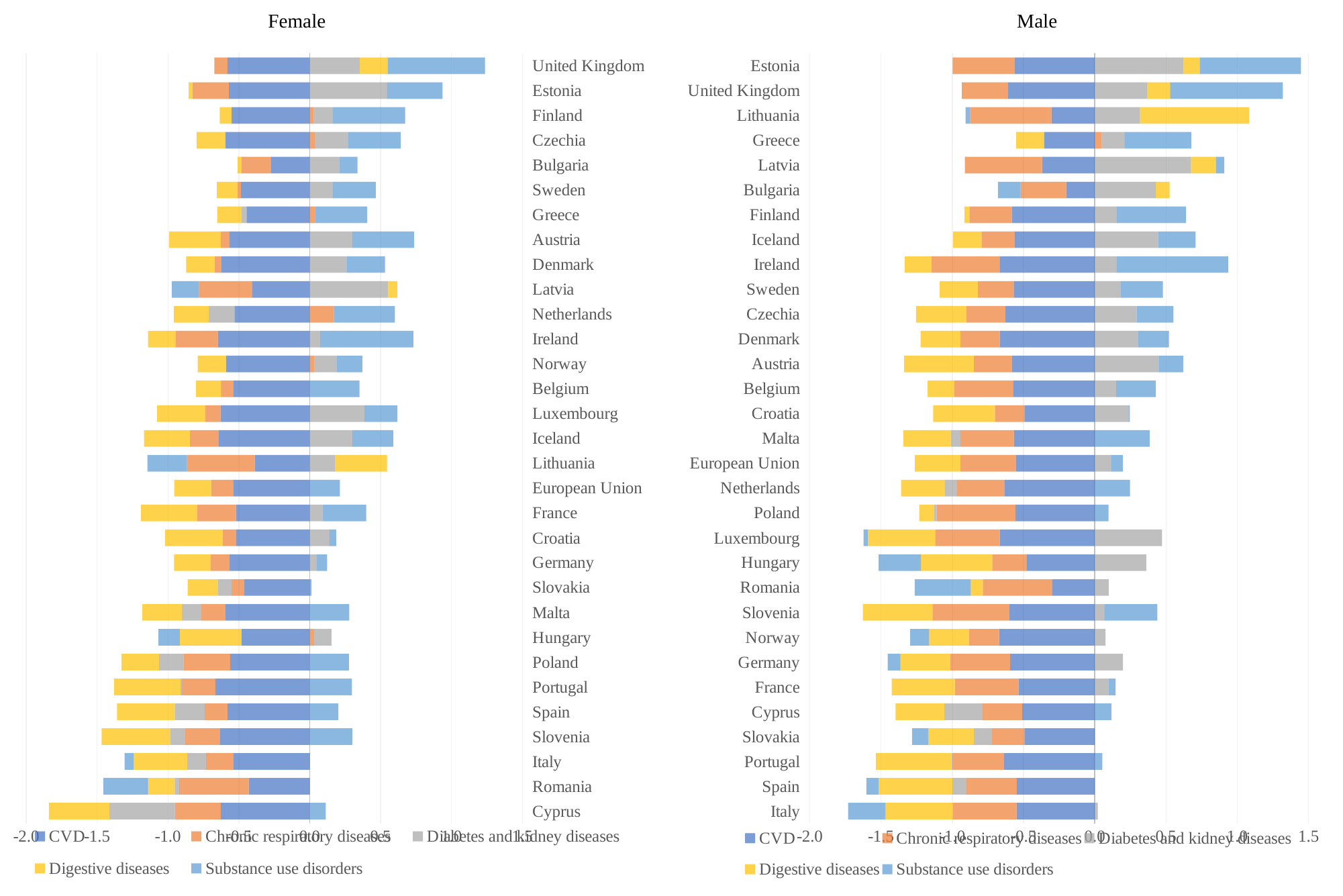

Female
Male
### Chart
| Category | CVD | Chronic respiratory diseases | Diabetes and kidney diseases | Digestive diseases | Substance use disorders |
|---|---|---|---|---|---|
| Italy | -0.54891001649951 | -0.448433035531289 | 0.0214613431475732 | -0.471360544325357 | -0.262414095955543 |
| Spain | -0.547188302925773 | -0.352263621788101 | -0.0997998854422651 | -0.518702769438358 | -0.0845716762122651 |
| Portugal | -0.635662318192451 | -0.36571728588441 | -0.00435744838645166 | -0.529621092922477 | 0.0522772229664038 |
| Slovakia | -0.492984443086878 | -0.227900299855709 | -0.12841348073552 | -0.318183687263815 | -0.11506122445173 |
| Cyprus | -0.509865469258974 | -0.276778564024011 | -0.269623829061348 | -0.341174191647301 | 0.116437151873195 |
| France | -0.534816457698248 | -0.447428306127432 | 0.0962611813778599 | -0.443345520475074 | 0.0498673150144222 |
| Germany | -0.594111221839585 | -0.421609262611672 | 0.198058289275711 | -0.348001294142564 | -0.0899949560151804 |
| Norway | -0.671646947691743 | -0.212294177580271 | 0.0757763492416292 | -0.278547756865792 | -0.132676483172172 |
| Slovenia | -0.597974627459837 | -0.541057329569986 | 0.0695841133067173 | -0.487932801180671 | 0.369854510323835 |
| Romania | -0.300421935108714 | -0.485018533795137 | 0.097877530447922 | -0.0853714449070285 | -0.393326456895645 |
| Hungary | -0.47755655653169 | -0.242173561192463 | 0.361055453188508 | -0.499983797159457 | -0.297296173402802 |
| Luxembourg | -0.667214962024434 | -0.453275767585697 | 0.472053357258717 | -0.47290164639626 | -0.0293823076056136 |
| Poland | -0.55579808262373 | -0.553820829557366 | -0.0190800292834409 | -0.1032094766861 | 0.0967356484026916 |
| Netherlands | -0.635133909613401 | -0.33224875871505 | -0.0846319452434373 | -0.307094199446633 | 0.247386284497628 |
| European Union | -0.551219908457951 | -0.391002464085211 | 0.116448186069104 | -0.320045201755557 | 0.0807203429280192 |
| Malta | -0.567376222433599 | -0.376373739600535 | -0.0669651722241138 | -0.333429326077407 | 0.384375937537058 |
| Croatia | -0.494001557646438 | -0.20705466448627 | 0.236469499890156 | -0.43273896491683 | 0.0092691833252863 |
| Belgium | -0.571282020140669 | -0.414094579442734 | 0.150141376758208 | -0.187358022918227 | 0.278319693968064 |
| Austria | -0.579434004462172 | -0.268963002981743 | 0.448868667931761 | -0.489279197626038 | 0.171926086253626 |
| Denmark | -0.667120651225474 | -0.276890633287789 | 0.306547953742093 | -0.278576539354761 | 0.213555907927364 |
| Czechia | -0.626161384502127 | -0.275457684105582 | 0.294066316978097 | -0.352545705674325 | 0.257691218408203 |
| Sweden | -0.568766569874841 | -0.25089510349568 | 0.182984426090869 | -0.268875598290048 | 0.295118194234913 |
| Ireland | -0.666134428198559 | -0.480449871492943 | 0.151922554233847 | -0.186817681207363 | 0.78498996824399 |
| Iceland | -0.560615962007779 | -0.234246897767141 | 0.444929706317991 | -0.199649790829844 | 0.262366872778959 |
| Finland | -0.581431585925485 | -0.29914402564427 | 0.154994766524772 | -0.0338068482310704 | 0.485098516253909 |
| Bulgaria | -0.200358331303366 | -0.32027544935594 | 0.428605637850594 | 0.0961204924965216 | -0.159158865003939 |
| Latvia | -0.370189150042221 | -0.541832120165068 | 0.675883151108403 | 0.173781861881043 | 0.059115834104842 |
| Greece | -0.354456846870567 | 0.0491270748104718 | 0.161290941421894 | -0.197103684219505 | 0.466238932058889 |
| Lithuania | -0.303720260516436 | -0.571889851459739 | 0.318080513148319 | 0.76335391310419 | -0.031147373935586 |
| United Kingdom | -0.607631772980569 | -0.32560172953413 | 0.369251760300024 | 0.159291873176173 | 0.790938246924559 |
| Estonia | -0.560294356106016 | -0.43757394888979 | 0.620675387324865 | 0.117165511817726 | 0.709195105700494 |
### Chart
| Category | CVD | Chronic respiratory diseases | Diabetes and kidney diseases | Digestive diseases | Substance use disorders |
|---|---|---|---|---|---|
| Cyprus | -0.628046335959626 | -0.32300205226464 | -0.462112895364316 | -0.426875938736711 | 0.112429820675438 |
| Romania | -0.430258563993824 | -0.489541826409311 | -0.0318650956239937 | -0.188639973040795 | -0.315543802830841 |
| Italy | -0.538329163884926 | -0.192798779053989 | -0.132144327762045 | -0.380064335331556 | -0.0628200467373871 |
| Slovenia | -0.635107624755709 | -0.242328157836234 | -0.10657849703798 | -0.484470229716432 | 0.300020194046166 |
| Spain | -0.579541104169246 | -0.161301100606805 | -0.211196509965869 | -0.406932278076439 | 0.202429614176807 |
| Portugal | -0.666102622259331 | -0.235283727868258 | -0.0110033041215978 | -0.468584557131957 | 0.297365736107381 |
| Poland | -0.562294172067933 | -0.328287187701217 | -0.17282128987006 | -0.263538942912123 | 0.276595305691005 |
| Hungary | -0.48251181816596 | 0.0297394952915054 | 0.123945620514347 | -0.433802689087853 | -0.152263672088398 |
| Malta | -0.596744192592908 | -0.167585418861745 | -0.137253413518377 | -0.279498826119818 | 0.278835847102097 |
| Slovakia | -0.461773719563644 | -0.0909633240865365 | -0.0971769212663806 | -0.211460664217712 | 0.0128875368818041 |
| Germany | -0.565651068739893 | -0.132359260743801 | 0.0468545578051525 | -0.258962556538669 | 0.0749098412601704 |
| Croatia | -0.517684306814421 | -0.0952394600277303 | 0.139299296659879 | -0.407829658455841 | 0.0475862147164023 |
| France | -0.519732134843681 | -0.275451941106695 | 0.0904012671231342 | -0.395344846993159 | 0.307647988393462 |
| European Union | -0.538059449587218 | -0.156132681056446 | -0.00124215726824789 | -0.260330222836171 | 0.21215073585761 |
| Lithuania | -0.38729858679925 | -0.478496726939294 | 0.180648549806146 | 0.364553047768061 | -0.279828536556228 |
| Iceland | -0.644096564113595 | -0.199949315737409 | 0.300700301181416 | -0.325275194898185 | 0.287753504939476 |
| Luxembourg | -0.628999368230534 | -0.10743301413247 | 0.386628760803063 | -0.340192574055153 | 0.231488318858396 |
| Belgium | -0.538161869367976 | -0.0848467757846782 | -0.00490647059233475 | -0.174532950130503 | 0.350891784490823 |
| Norway | -0.591226984212168 | 0.0338927318824423 | 0.158925226852692 | -0.198723234551259 | 0.178721373033511 |
| Ireland | -0.647516034545075 | -0.300173416809704 | 0.0736610183248749 | -0.19299321401848 | 0.657562208342403 |
| Netherlands | -0.530563532763189 | 0.171018308395886 | -0.181928446912812 | -0.245390958597403 | 0.429783180535351 |
| Latvia | -0.407669803725594 | -0.374373354303726 | 0.553610575809565 | 0.0638924729941717 | -0.19180619890832 |
| Denmark | -0.625704661065184 | -0.0457373512604482 | 0.260470313231061 | -0.200312188797363 | 0.270780692010411 |
| Austria | -0.564898320993776 | -0.0632156635714407 | 0.300666976640128 | -0.364502663171317 | 0.434630028964112 |
| Greece | -0.445576478299878 | 0.0373860675437955 | -0.0381691998456036 | -0.169721488985188 | 0.367542656131494 |
| Sweden | -0.486447907526607 | -0.0238594499899742 | 0.164526955322835 | -0.144688998530632 | 0.301490920251818 |
| Bulgaria | -0.27233652313954 | -0.209146477647079 | 0.209682642752426 | -0.0268276666876387 | 0.12767961962147 |
| Czechia | -0.595313140984804 | 0.0351177264419052 | 0.234273248327565 | -0.203370386237075 | 0.373143851086036 |
| Finland | -0.551125732584279 | 0.0284723580276009 | 0.132342176494486 | -0.0835179364666667 | 0.512596594678855 |
| Estonia | -0.569856355651661 | -0.257625278403056 | 0.546387025339877 | -0.0268822224604976 | 0.390170135056321 |
| United Kingdom | -0.580541087551596 | -0.0928677411567177 | 0.352567826583153 | 0.198216776198028 | 0.685431572871984 |
