# Supplementary material for: Inequalities in the burden of non-communicable diseases across European countries: a systematic analysis of the Global Burden of Disease 2019 study
Source: Int J Equity Health. 2023 Jul 28;22:140. doi: 10.1186/s12939-023-01958-8 (PMC10375608; doi:10.1186/s12939-023-01958-8)
Supplement: Supplementary file 3 — Additional file 3: Figure: Slope Index of Inequality of age-standardized level 1 and 2 NCDs DALYs rates, 1990–2019. Legend: CVDs: cardiovascular diseases; CI: Confidence Interval; NCDs: non-communicable diseases; Coef.: Coefficient. [file 12939_2023_1958_MOESM3_ESM.docx]

| Cause | 1990 | | | 1991 | | | | 1992 | | | | 1993 | | | | 1994 | | | | | 1995 | | | |
| --- | --- | --- | --- | --- | --- | --- | --- | --- | --- | --- | --- | --- | --- | --- | --- | --- | --- | --- | --- | --- | --- | --- | --- | --- |
|  | **Coef.** | **95% CI** | | | **Coef.** | **95% CI** | | | **Coef.** | **95% CI** | | | **Coef.** | **95% CI** | | | **Coef.** | **95% CI** | | | **Coef.** | **95% CI** | | |
| CVDs | 0.852 | 0.708 | 0.997 | | 0.884 | 0.725 | 1.043 | | 0.909 | 0.734 | 1.084 | | 0.947 | 0.751 | 1.142 | | 0.997 | 0.776 | 1.245 | 0.982 | | 0.750 | 1.214 |  |
| Chronic respiratory diseases | 0.078 | 0.071 | 0.086 | | 0.081 | 0.074 | 0.087 | | 0.080 | 0.073 | 0.087 | | 0.077 | 0.069 | 0.085 | | 0.076 | 0.068 | 0.084 | 0.077 | | 0.068 | 0.085 |  |
| Diabetes and kidney diseases | 0.078 | 0.061 | 0.096 | | 0.081 | 0.063 | 0.099 | | 0.080 | 0.060 | 0.099 | | 0.078 | 0.058 | 0.099 | | 0.080 | 0.060 | 0.101 | 0.078 | | 0.058 | 0.098 |  |
| Digestive diseases | 0.110 | 0.089 | 0.131 | | 0.114 | 0.091 | 0.136 | | 0.115 | 0.088 | 0.143 | | 0.111 | 0.080 | 0.142 | | 0.108 | 0.076 | 0.139 | 0.107 | | 0.076 | 0.139 |  |
| Mental disorders | 0.087 | 0.071 | 0.104 | | 0.086 | 0.070 | 0.103 | | 0.083 | 0.067 | 0.099 | | 0.083 | 0.066 | 0.099 | | 0.082 | 0.066 | 0.098 | 0.082 | | 0.066 | 0.098 |  |
| Musculoskeletal disorders | 0.100 | 0.092 | 0.108 | | 0.102 | 0.093 | 0.110 | | 0.101 | 0.092 | 0.109 | | 0.099 | 0.090 | 0.108 | | 0.099 | 0.091 | 0.107 | 0.099 | | 0.091 | 0.107 |  |
| Neoplasms | 0.161 | 0.136 | 0.185 | | 0.162 | 0.136 | 0.187 | | 0.159 | 0.130 | 0.187 | | 0.168 | 0.140 | 0.195 | | 0.178 | 0.148 | 0.208 | 0.173 | | 0.143 | 0.203 |  |
| Neurological disorders | 0.021 | 0.019 | 0.023 | | 0.021 | 0.019 | 0.023 | | 0.021 | 0.019 | 0.023 | | 0.022 | 0.020 | 0.023 | | 0.021 | 0.019 | 0.023 | 0.022 | | 0.020 | 0.024 |  |
| NCDs | 0.851 | 0.730 | 0.972 | | 0.869 | 0.741 | 0.997 | | 0.888 | 0.748 | 1.029 | | 0.951 | 0.781 | 1.121 | | 0.998 | 0.803 | 1.220 | 0.986 | | 0.795 | 1.178 |  |
| Other NCDs | 0.045 | 0.038 | 0.051 | | 0.048 | 0.040 | 0.056 | | 0.052 | 0.042 | 0.062 | | 0.054 | 0.043 | 0.064 | | 0.058 | 0.048 | 0.068 | 0.056 | | 0.046 | 0.066 |  |
| Sense organ diseases | 0.035 | 0.032 | 0.039 | | 0.036 | 0.032 | 0.040 | | 0.035 | 0.032 | 0.039 | | 0.035 | 0.032 | 0.039 | | 0.035 | 0.032 | 0.039 | 0.035 | | 0.031 | 0.039 |  |
| Skin and subcutaneous diseases | 0.038 | 0.031 | 0.045 | | 0.039 | 0.032 | 0.046 | | 0.039 | 0.032 | 0.046 | | 0.039 | 0.032 | 0.046 | | 0.039 | 0.032 | 0.046 | 0.039 | | 0.032 | 0.046 |  |
| Substance use disorders | 0.051 | 0.040 | 0.063 | | 0.054 | 0.041 | 0.067 | | 0.056 | 0.042 | 0.070 | | 0.060 | 0.043 | 0.078 | | 0.065 | 0.045 | 0.086 | 0.076 | | 0.054 | 0.098 |  |
| Cause | **1996** | | | **1997** | | | | **1998** | | | | **1999** | | | | **2000** | | | | | **2001** | | | |
|  | **Coef.** | **95% CI** | | | **Coef.** | **95% CI** | | | **Coef.** | **95% CI** | | | **Coef.** | **95% CI** | | | **Coef.** | **95% CI** | | | **Coef.** | **95% CI** | | |
| CVDs | 0.909 | 0.677 | 1.140 | | 0.911 | 0.662 | 1.160 | | 0.892 | 0.639 | 1.146 | | 0.847 | 0.608 | 1.086 | | 0.784 | 0.549 | 1.019 | 0.809 | | 0.569 | 1.050 |  |
| Chronic respiratory diseases | 0.077 | 0.069 | 0.086 | | 0.074 | 0.065 | 0.083 | | 0.072 | 0.063 | 0.081 | | 0.073 | 0.064 | 0.082 | | 0.072 | 0.062 | 0.081 | 0.074 | | 0.064 | 0.084 |  |
| Diabetes and kidney diseases | 0.076 | 0.057 | 0.095 | | 0.073 | 0.055 | 0.091 | | 0.069 | 0.052 | 0.087 | | 0.068 | 0.051 | 0.085 | | 0.067 | 0.051 | 0.083 | 0.064 | | 0.049 | 0.079 |  |
| Digestive diseases | 0.108 | 0.079 | 0.136 | | 0.107 | 0.078 | 0.135 | | 0.106 | 0.078 | 0.134 | | 0.104 | 0.077 | 0.131 | | 0.101 | 0.077 | 0.125 | 0.100 | | 0.079 | 0.122 |  |
| Mental disorders | 0.083 | 0.067 | 0.099 | | 0.085 | 0.069 | 0.101 | | 0.087 | 0.072 | 0.103 | | 0.092 | 0.076 | 0.108 | | 0.092 | 0.075 | 0.108 | 0.092 | | 0.075 | 0.108 |  |
| Musculoskeletal disorders | 0.099 | 0.091 | 0.108 | | 0.098 | 0.089 | 0.107 | | 0.099 | 0.090 | 0.108 | | 0.100 | 0.091 | 0.109 | | 0.100 | 0.091 | 0.109 | 0.100 | | 0.090 | 0.110 |  |
| Neoplasms | 0.155 | 0.127 | 0.183 | | 0.157 | 0.128 | 0.186 | | 0.157 | 0.125 | 0.188 | | 0.157 | 0.122 | 0.191 | | 0.147 | 0.114 | 0.180 | 0.151 | | 0.121 | 0.182 |  |
| Neurological disorders | 0.023 | 0.021 | 0.025 | | 0.024 | 0.021 | 0.026 | | 0.026 | 0.023 | 0.028 | | 0.027 | 0.025 | 0.030 | | 0.028 | 0.026 | 0.031 | 0.029 | | 0.026 | 0.032 |  |
| NCDs | 0.917 | 0.739 | 1.096 | | 0.917 | 0.732 | 1.103 | | 0.911 | 0.723 | 1.100 | | 0.859 | 0.692 | 1.025 | | 0.815 | 0.657 | 0.972 | 0.833 | | 0.664 | 1.001 |  |
| Other NCDs | 0.054 | 0.045 | 0.063 | | 0.053 | 0.043 | 0.063 | | 0.049 | 0.039 | 0.059 | | 0.046 | 0.037 | 0.055 | | 0.048 | 0.039 | 0.056 | 0.050 | | 0.042 | 0.058 |  |
| Sense organ diseases | 0.035 | 0.031 | 0.039 | | 0.034 | 0.031 | 0.038 | | 0.033 | 0.030 | 0.037 | | 0.033 | 0.030 | 0.036 | | 0.033 | 0.030 | 0.036 | 0.033 | | 0.029 | 0.036 |  |
| Skin and subcutaneous diseases | 0.039 | 0.032 | 0.046 | | 0.039 | 0.032 | 0.046 | | 0.039 | 0.031 | 0.046 | | 0.039 | 0.032 | 0.047 | | 0.039 | 0.032 | 0.047 | 0.040 | | 0.032 | 0.047 |  |
| Substance use disorders | 0.063 | 0.045 | 0.081 | | 0.062 | 0.046 | 0.078 | | 0.064 | 0.048 | 0.079 | | 0.064 | 0.049 | 0.079 | | 0.064 | 0.049 | 0.080 | 0.069 | | 0.052 | 0.086 |  |
| Cause | **2002** | | | **2003** | | | | **2004** | | | | **2005** | | | | **2006** | | | | | **2007** | | | |
|  | **Coef.** | **95% CI** | | | **Coef.** | **95% CI** | | | **Coef.** | **95% CI** | | | **Coef.** | **95% CI** | | | **Coef.** | **95% CI** | | | **Coef.** | **95% CI** | | |
| CVDs | 0.794 | 0.555 | 1.034 | | 0.757 | 0.527 | 0.987 | | 0.747 | 0.519 | 0.975 | | 0.772 | 0.538 | 1.005 | | 0.797 | 0.564 | 1.030 | 0.784 | | 0.569 | 1.000 |  |
| Chronic respiratory diseases | 0.073 | 0.064 | 0.082 | | 0.071 | 0.062 | 0.080 | | 0.068 | 0.060 | 0.077 | | 0.067 | 0.058 | 0.075 | | 0.067 | 0.058 | 0.075 | 0.067 | | 0.058 | 0.075 |  |
| Diabetes and kidney diseases | 0.063 | 0.048 | 0.077 | | 0.062 | 0.049 | 0.076 | | 0.062 | 0.049 | 0.075 | | 0.062 | 0.050 | 0.075 | | 0.061 | 0.048 | 0.074 | 0.062 | | 0.050 | 0.074 |  |
| Digestive diseases | 0.098 | 0.077 | 0.119 | | 0.098 | 0.078 | 0.119 | | 0.096 | 0.076 | 0.115 | | 0.099 | 0.080 | 0.119 | | 0.102 | 0.082 | 0.123 | 0.106 | | 0.084 | 0.128 |  |
| Mental disorders | 0.091 | 0.073 | 0.108 | | 0.085 | 0.067 | 0.103 | | 0.081 | 0.062 | 0.101 | | 0.080 | 0.061 | 0.100 | | 0.080 | 0.061 | 0.099 | 0.083 | | 0.064 | 0.102 |  |
| Musculoskeletal disorders | 0.099 | 0.090 | 0.109 | | 0.099 | 0.090 | 0.108 | | 0.102 | 0.094 | 0.111 | | 0.101 | 0.094 | 0.109 | | 0.101 | 0.093 | 0.108 | 0.099 | | 0.091 | 0.106 |  |
| Neoplasms | 0.148 | 0.116 | 0.179 | | 0.150 | 0.118 | 0.183 | | 0.146 | 0.114 | 0.178 | | 0.155 | 0.123 | 0.187 | | 0.157 | 0.126 | 0.187 | 0.161 | | 0.130 | 0.192 |  |
| Neurological disorders | 0.030 | 0.027 | 0.032 | | 0.030 | 0.027 | 0.033 | | 0.030 | 0.028 | 0.033 | | 0.030 | 0.028 | 0.033 | | 0.030 | 0.027 | 0.033 | 0.030 | | 0.028 | 0.033 |  |
| NCDs | 0.838 | 0.671 | 1.005 | | 0.820 | 0.658 | 0.982 | | 0.818 | 0.652 | 0.983 | | 0.849 | 0.671 | 1.027 | | 0.869 | 0.678 | 1.060 | 0.871 | | 0.679 | 1.063 |  |
| Other NCDs | 0.050 | 0.043 | 0.058 | | 0.050 | 0.043 | 0.057 | | 0.049 | 0.041 | 0.056 | | 0.047 | 0.040 | 0.055 | | 0.047 | 0.039 | 0.055 | 0.046 | | 0.039 | 0.054 |  |
| Sense organ diseases | 0.033 | 0.029 | 0.036 | | 0.032 | 0.029 | 0.036 | | 0.031 | 0.027 | 0.034 | | 0.030 | 0.027 | 0.034 | | 0.030 | 0.027 | 0.034 | 0.030 | | 0.027 | 0.034 |  |
| Skin and subcutaneous diseases | 0.041 | 0.033 | 0.048 | | 0.041 | 0.034 | 0.048 | | 0.041 | 0.034 | 0.048 | | 0.041 | 0.034 | 0.048 | | 0.042 | 0.034 | 0.049 | 0.041 | | 0.034 | 0.049 |  |
| Substance use disorders | 0.068 | 0.052 | 0.084 | | 0.068 | 0.052 | 0.000 | | 0.069 | 0.052 | 0.087 | | 0.074 | 0.056 | 0.091 | | 0.074 | 0.057 | 0.091 | 0.076 | | 0.058 | 0.094 |  |
| Cause | **2008** | | | **2009** | | | | **2010** | | | | **2011** | | | | **2012** | | | | | **2013** | | | |
|  | **Coef.** | **95% CI** | | | **Coef.** | **95% CI** | | | **Coef.** | **95% CI** | | | **Coef.** | **95% CI** | | | **Coef.** | **95% CI** | | | **Coef.** | **95% CI** | | |
| CVDs | 0.726 | 0.526 | 0.925 | | 0.697 | 0.506 | 0.889 | | 0.671 | 0.485 | 0.857 | | 0.651 | 0.471 | 0.831 | | 0.617 | 0.444 | 0.789 | 0.596 | | 0.429 | 0.763 |  |
| Chronic respiratory diseases | 0.063 | 0.055 | 0.072 | | 0.064 | 0.056 | 0.072 | | 0.065 | 0.057 | 0.073 | | 0.064 | 0.056 | 0.072 | | 0.063 | 0.056 | 0.071 | 0.063 | | 0.055 | 0.070 |  |
| Diabetes and kidney diseases | 0.062 | 0.051 | 0.073 | | 0.060 | 0.051 | 0.070 | | 0.060 | 0.050 | 0.070 | | 0.059 | 0.050 | 0.068 | | 0.058 | 0.050 | 0.067 | 0.057 | | 0.048 | 0.066 |  |
| Digestive diseases | 0.105 | 0.084 | 0.125 | | 0.100 | 0.081 | 0.118 | | 0.097 | 0.078 | 0.115 | | 0.093 | 0.077 | 0.109 | | 0.091 | 0.075 | 0.107 | 0.088 | | 0.073 | 0.104 |  |
| Mental disorders | 0.086 | 0.066 | 0.105 | | 0.087 | 0.068 | 0.106 | | 0.086 | 0.066 | 0.106 | | 0.086 | 0.066 | 0.106 | | 0.086 | 0.066 | 0.106 | 0.083 | | 0.062 | 0.104 |  |
| Musculoskeletal disorders | 0.098 | 0.090 | 0.105 | | 0.097 | 0.088 | 0.106 | | 0.097 | 0.088 | 0.105 | | 0.096 | 0.088 | 0.105 | | 0.095 | 0.086 | 0.104 | 0.093 | | 0.083 | 0.102 |  |
| Neoplasms | 0.159 | 0.133 | 0.186 | | 0.156 | 0.130 | 0.182 | | 0.153 | 0.129 | 0.177 | | 0.148 | 0.124 | 0.172 | | 0.146 | 0.123 | 0.169 | 0.139 | | 0.118 | 0.160 |  |
| Neurological disorders | 0.030 | 0.027 | 0.033 | | 0.031 | 0.028 | 0.033 | | 0.031 | 0.028 | 0.033 | | 0.031 | 0.028 | 0.034 | | 0.032 | 0.029 | 0.034 | 0.032 | | 0.030 | 0.034 |  |
| NCDs | 0.817 | 0.648 | 0.986 | | 0.746 | 0.590 | 0.903 | | 0.716 | 0.565 | 0.866 | | 0.680 | 0.544 | 0.816 | | 0.680 | 0.553 | 0.807 | 0.654 | | 0.527 | 0.780 |  |
| Other NCDs | 0.046 | 0.040 | 0.053 | | 0.046 | 0.040 | 0.052 | | 0.046 | 0.040 | 0.051 | | 0.046 | 0.040 | 0.052 | | 0.045 | 0.039 | 0.051 | 0.046 | | 0.040 | 0.052 |  |
| Sense organ diseases | 0.031 | 0.028 | 0.034 | | 0.031 | 0.028 | 0.034 | | 0.031 | 0.028 | 0.034 | | 0.031 | 0.028 | 0.034 | | 0.031 | 0.028 | 0.034 | 0.031 | | 0.028 | 0.034 |  |
| Skin and subcutaneous diseases | 0.041 | 0.034 | 0.049 | | 0.042 | 0.034 | 0.049 | | 0.041 | 0.034 | 0.049 | | 0.041 | 0.034 | 0.048 | | 0.041 | 0.034 | 0.048 | 0.040 | | 0.033 | 0.048 |  |
| Substance use disorders | 0.075 | 0.058 | 0.092 | | 0.074 | 0.058 | 0.091 | | 0.075 | 0.058 | 0.091 | | 0.075 | 0.058 | 0.092 | | 0.074 | 0.056 | 0.093 | 0.075 | | 0.058 | 0.093 |  |
| Cause | **2014** | | | **2015** | | | | **2016** | | | | **2017** | | | | **2018** | | | | | **2019** | | | |
|  | **Coef.** | **95% CI** | | | **Coef.** | **95% CI** | | | **Coef.** | **95% CI** | | | **Coef.** | **95% CI** | | | **Coef.** | **95% CI** | | | **Coef.** | **95% CI** | | |
| CVDs | 0.575 | 0.411 | 0.739 | | 0.564 | 0.410 | 0.718 | | 0.556 | 0.403 | 0.709 | | 0.544 | 0.395 | 0.694 | | 0.537 | 0.387 | 0.687 | 0.531 | | 0.381 | 0.681 |  |
| Chronic respiratory diseases | 0.062 | 0.054 | 0.069 | | 0.062 | 0.055 | 0.069 | | 0.062 | 0.055 | 0.069 | | 0.061 | 0.054 | 0.068 | | 0.062 | 0.055 | 0.069 | 0.061 | | 0.054 | 0.068 |  |
| Diabetes and kidney diseases | 0.057 | 0.048 | 0.066 | | 0.058 | 0.050 | 0.067 | | 0.057 | 0.048 | 0.066 | | 0.055 | 0.046 | 0.065 | | 0.056 | 0.047 | 0.065 | 0.057 | | 0.048 | 0.066 |  |
| Digestive diseases | 0.088 | 0.073 | 0.104 | | 0.089 | 0.074 | 0.104 | | 0.087 | 0.072 | 0.102 | | 0.084 | 0.070 | 0.098 | | 0.083 | 0.070 | 0.097 | 0.083 | | 0.070 | 0.096 |  |
| Mental disorders | 0.083 | 0.063 | 0.103 | | 0.084 | 0.063 | 0.104 | | 0.084 | 0.064 | 0.105 | | 0.084 | 0.064 | 0.104 | | 0.083 | 0.063 | 0.102 | 0.086 | | 0.068 | 0.104 |  |
| Musculoskeletal disorders | 0.087 | 0.076 | 0.098 | | 0.085 | 0.074 | 0.097 | | 0.090 | 0.081 | 0.100 | | 0.090 | 0.081 | 0.100 | | 0.090 | 0.080 | 0.099 | 0.089 | | 0.079 | 0.098 |  |
| Neoplasms | 0.142 | 0.118 | 0.166 | | 0.143 | 0.119 | 0.167 | | 0.134 | 0.111 | 0.156 | | 0.132 | 0.110 | 0.155 | | 0.134 | 0.112 | 0.156 | 0.132 | | 0.111 | 0.153 |  |
| Neurological disorders | 0.032 | 0.030 | 0.035 | | 0.033 | 0.031 | 0.035 | | 0.032 | 0.030 | 0.034 | | 0.031 | 0.029 | 0.034 | | 0.031 | 0.028 | 0.033 | 0.030 | | 0.028 | 0.032 |  |
| NCDs | 0.655 | 0.523 | 0.788 | | 0.628 | 0.503 | 0.752 | | 0.616 | 0.490 | 0.742 | | 0.595 | 0.472 | 0.718 | | 0.594 | 0.469 | 0.719 | 0.592 | | 0.470 | 0.715 |  |
| Other NCDs | 0.045 | 0.039 | 0.052 | | 0.047 | 0.041 | 0.053 | | 0.049 | 0.044 | 0.055 | | 0.047 | 0.041 | 0.054 | | 0.047 | 0.041 | 0.054 | 0.047 | | 0.041 | 0.053 |  |
| Sense organ diseases | 0.031 | 0.028 | 0.034 | | 0.032 | 0.029 | 0.035 | | 0.032 | 0.029 | 0.035 | | 0.032 | 0.029 | 0.035 | | 0.032 | 0.029 | 0.035 | 0.032 | | 0.028 | 0.035 |  |
| Skin and subcutaneous diseases | 0.039 | 0.032 | 0.047 | | 0.039 | 0.032 | 0.046 | | 0.039 | 0.032 | 0.046 | | 0.039 | 0.032 | 0.045 | | 0.039 | 0.031 | 0.046 | 0.039 | | 0.031 | 0.047 |  |
| Substance use disorders | 0.079 | 0.061 | 0.000 | | 0.078 | 0.062 | 0.094 | | 0.080 | 0.063 | 0.096 | | 0.078 | 0.060 | 0.095 | | 0.080 | 0.062 | 0.098 | 0.078 | | 0.060 | 0.096 |  |
